# AI-based prediction of depression symptomatology in first-episode psychosis patients: insights from the EUFEST and RAISE-ETP clinical trials

**DOI:** 10.1017/S0033291725100950

**Published:** 2025-07-30

**Authors:** Sergio Mena, Fiona Coutts, Jana von Trott, Esin Ucur, Clara Vetter, René R. Kahn, W. Wolfgang Fleischhacker, John M. Kane, Oliver D. Howes, Rachel Upthegrove, Paris A. Lalousis, Nikolaos Koutsouleris

**Affiliations:** 1 Department of Psychosis Studies, Institute of Psychiatry, Psychology and Neuroscience, King’s College London, London, UK; 2 Department of Psychiatry and Psychotherapy, Ludwig-Maximilians-University, Munich, Germany; 3 Department of Psychiatry, Icahn School of Medicine at Mount Sinai, New York, NY, USA; 4 Department of Psychiatry, University Medical Center Utrecht, Utrecht, the Netherlands; 5 Department of Biological Psychiatry, Medical University Innsbruck, Innsbruck, Austria; 6 The Zucker Hillside Hospital, Psychiatry Research, Northwell Health, Glen Oaks, NY, USA; 7 Department of Psychiatry University of Oxford, Oxford, UK; 8 Institute for Mental Health, School of Psychology, University of Birmingham, Birmingham, UK; 9 Max Planck Institute of Psychiatry, Munich, Germany; 10 German Center for Mental Health (DZPG), Munich-Augsburg, Germany

**Keywords:** depression, first-episode psychosis, schizophrenia, artificial intelligence, predictive modelling, machine learning, support vector machines, clinical prognosis, biomarkers, precision psychiatry

## Abstract

**Background:**

Depressive symptoms are highly prevalent in first-episode psychosis (FEP) and worsen clinical outcomes. It is currently difficult to determine which patients will have persistent depressive symptoms based on a clinical assessment. We aimed to determine whether depressive symptoms and post-psychotic depressive episodes can be predicted from baseline clinical data, quality of life, and blood-based biomarkers, and to assess the geographical generalizability of these models.

**Methods:**

Two FEP trials were analyzed: European First-Episode Schizophrenia Trial (EUFEST) (*n* = 498; 2002–2006) and Recovery After an Initial Schizophrenia Episode Early Treatment Program (RAISE-ETP) (*n* = 404; 2010–2012). Participants included those aged 15–40 years, meeting Diagnostic and Statistical Manual of Mental Disorders IV criteria for schizophrenia spectrum disorders. We developed support vector regressors and classifiers to predict changes in depressive symptoms at 6 and 12 months and depressive episodes within the first 6 months. These models were trained in one sample and externally validated in another for geographical generalizability.

**Results:**

A total of 320 EUFEST and 234 RAISE-ETP participants were included (mean [SD] age: 25.93 [5.60] years, 56.56% male; 23.90 [5.27] years, 73.50% male). Models predicted changes in depressive symptoms at 6 months with balanced accuracy (BAC) of 66.26% (RAISE-ETP) and 75.09% (EUFEST), and at 12 months with BAC of 67.88% (RAISE-ETP) and 77.61% (EUFEST). Depressive episodes were predicted with BAC of 66.67% (RAISE-ETP) and 69.01% (EUFEST), showing fair external predictive performance.

**Conclusions:**

Predictive models using clinical data, quality of life, and biomarkers accurately forecast depressive events in FEP, demonstrating generalization across populations.

## Introduction

Depressive syndromes frequently predate, parallel, or follow the first-episode psychosis (FEP) (Upthegrove et al., 2010). Depressive comorbidity in psychotic disorder rates vary depending on criteria and illness stage, and are reported to range from 26% up to 80% (Birchwood, Iqbal, Chadwick, & Trower, 2000; Conley, Ascher-Svanum, Zhu, Faries, & Kinon, 2007; Cotton et al., 2012; Upthegrove et al., 2010). These symptoms may stem from neurobiological processes inherent to psychosis (Alexandros Lalousis et al., 2023; Häfner et al., 2005), as a result of the dysphoric effects of antipsychotic medication (Voruganti & Awad, 2004), or as a psychological response to the debilitating and life-changing impact of psychosis (Upthegrove, Marwaha, & Birchwood, 2017). As a result of all these factors, ~80% of psychosis patients experience a major depressive episode over the course of their illness (Upthegrove et al., 2010). These rates far exceed the lifetime prevalence of major depressive episodes in the general population, reported to be from 14% up to 24% (Bromet et al., 2011; Davis et al., 2020).

Studying depressive symptoms in patients with psychosis is crucial due to the association between comorbid depression and poor clinical outcomes and quality of life of individuals at risk for psychosis (Solmi et al., 2023) and FEP patients (Conley et al., 2007). Suicidal behavior remains a critical and life-threatening concern among patients with psychotic disorders, with those who have comorbid depression being at the highest risk (McGinty & Upthegrove, 2020). As a result, clinical guidelines recommend monitoring coexisting conditions in psychosis, including depression (National Institute for Health and Care Excellence, 2014).

Although there is no clear consensus on treatment practices, recent evidence suggests that coadministering antidepressants with antipsychotics can enhance the remission of depressive symptoms in psychosis, potentially improving clinical outcomes without substantially increasing the burden of side effects (Barnes et al., 2019; Gregory, Mallikarjun, & Upthegrove, 2017). Current ongoing clinical trials are testing whether the addition of an antidepressant to antipsychotic medication following FEP is clinically useful in the prevention of post-psychotic depression (PPD) (Palmer et al., 2023). At the same time, evidence suggests that some FEP patients with comorbid depression will remit from depressive symptoms with an antipsychotic treatment alone (Phahladira et al., 2021). There is also historical, often conflicting, evidence that adjunctive antidepressants may worsen psychotic symptoms, suggesting that some FEP patients with comorbid depression will remit (Kramer et al., 1989; Lehman, Lieberman, Dixon, & Association, 2004; Preda, MacLean, Mazure, & Bowers, 2001). As a result, there is no consensus on the optimal strategy in the triage and management of depressive episodes before, during, and after the FEP, as well as in later psychotic stages (Barnes et al., 2019).

In this context, artificial intelligence offers a promising avenue to help address this challenge by identifying FEP patients at risk of experiencing recurrent depressive episodes following initiation of antipsychotic medication, and who may, therefore, benefit from adjunctive treatments. Despite significant advances in the application of artificial intelligence to prognostication in psychosis (Coutts, Koutsouleris, & McGuire, 2023; Saboori Amleshi et al., 2025), existing tools primarily focus on the prediction of remission defined using the core positive symptoms of psychosis (Coutts et al., 2023), and other outcome measures, such as functioning (Koutsouleris et al., 2016, 2018), and quality of life (Beaudoin, Hudon, Giguère, Potvin, & Dumais, 2022). Prognostication of depressive episodes following FEP is critical, as patients who experience these episodes tend to have a poorer recovery and a higher risk of psychotic relapse (Guerrero-Jiménez, Carrillo de Albornoz Calahorro, Girela-Serrano, Bodoano Sánchez, & Gutiérrez-Rojas, 2022; Jääskeläinen et al., 2018). Furthermore, the high comorbidity between psychosis and depression, along with numerous transdiagnostic findings that bridge these disorders across various biopsychosocial levels (Alexandros Lalousis et al., 2023), underscores the clinical importance of expanding the application of AI to study and predict depressive symptoms in psychosis. To our knowledge, no large-scale multivariate pattern recognition algorithms have been trained to predict comorbid depressive episodes and symptom trajectories in FEP and externally validated in independent cohorts.

The current work fills this gap by utilizing two large, multisite, and longitudinal clinical trials of minimally antipsychotic-treated FEP patients to predict depressive symptom trajectories, including the presence of post-psychotic depressive episode. These trials included the European First-Episode Schizophrenia Trial (EUFEST; ISRCTN68736636) and the Recovery After an Initial Schizophrenia Episode Early Treatment Program (RAISE-ETP; NCT01321177). The datasets comprised sociodemographic, clinical, treatment-related, quality-of-life, blood samples, and neurocognitive measures. Our objective was to predict future depression symptomatology in FEP patients by analyzing baseline variables using machine learning, to assess the prognostic specificity of these models for depressive symptoms and episodes, rather than negative symptoms, and to study the underlying patient and treatment characteristics that predict good versus poor depression outcomes.

## Methods

The Supplementary Methods section extensively details the methods of this study. Here, we provide a summary of the fundamental steps.

### Study design and data processing

Data from the EUFEST and RAISE-ETP trials were used. The study designs are detailed in previous publications (Fleischhacker, Keet, & Kahn, 2005; Kane et al., 2015b). Briefly, written informed consent was obtained from all participants. EUFEST recruited 498 patients (ages 18–40 years) meeting the Diagnostic and Statistical Manual of Mental Disorders, 4th Edition criteria for schizophrenia-spectrum disorders between 2002 and 2006 from 50 centers in 14 European countries and Israel. Participants received 1 year of treatment with antipsychotics, and outcomes included retention, psychotic symptoms assessed with the Positive and Negative Syndrome Scale (PANSS), depressive symptoms assessed using the Calgary Depression Scale for Schizophrenia (CDSS), side effects, and blood-based biomarkers like insulin, glucose, high-density lipoprotein, low-density lipoprotein, and triglycerides. RAISE-ETP recruited 404 patients (ages 15–40 years) from 34 sites across 21 US states between 2010 and 2012. Patients were allocated to either usual community care or the NAVIGATE program, with primary outcomes including quality of life, psychotic symptoms (PANSS), depressive symptoms (CDSS), and analogous blood-based biomarkers. A detailed record of the data domains and their corresponding collection dates in both studies is provided in Supplementary Tables S1 and S2. The trials complied with the ethical standards of the relevant national and institutional committees on human experimentation and with the Helsinki Declaration of 1975, as revised in 2013. All procedures involving patients were approved by the local ethics committees of the participating centers. Since this study is a secondary analysis of previously collected and anonymized data, additional ethical approval was not required.

In EUFEST, we restricted the study to 320 patients for whom outcomes at the 4-week, 6-month, and 12-month visits were available. In RAISE-ETP, we restricted the study to 234 patients for whom outcomes at the 6- and 12-month visits were available. In both cases, patients were also excluded when ≥20% of the baseline variables were missing. Included and excluded patients showed no significant differences in baseline characteristics for either sample (see Supplementary Tables S5 and S6). A full description of the data extraction is provided in Supplementary Methods. A preregistration of the study can be found on the Open Science Framework platform (osf.io/r95b2/).

### Statistical analyses

All group-level statistical analyses were performed using the SciPy library (1.10.0) with Python 3.11. The reported *p*-values are two-sided. First, univariate statistical methods were used to assess the differences between samples and classification groups. Two-sample *t*-tests were used to compare continuous variables, while the *χ* ^2^ test was used for categorical variables. All *p*-values were corrected for multiple comparisons using the Benjamini–Hochberg false discovery rate correction. For statistical analysis of effects in time series, we used mixed-design analysis of variance for comparisons involving a single between-subjects factor and mixed-effects linear regression models when considering multiple between-subjects factors. Statistical significance was defined at a threshold of *α* = 0.05.

### Machine learning analyses

Model development and validation were guided using the Transparent Reporting of a Multivariate Prediction Model for Individual Prognosis or Diagnosis guidelines. We used the MATLAB-based (2023b, MathWorks Inc.) machine learning software NeuroMiner version 1.2 preprocess features (scaling, pruning, and imputation of missing values), train, cross-validate, interpret, externally validate, and test the specificity of our models. Linear support vector machine (SVM) classifiers and regressors were trained and tested in a repeated, nested cross-validation framework with five outer and inner folds and permutations (see Supplementary Methods). In addition to these cross-validation experiments where patients were pooled across sites, we performed leave-site-out cross-validation analyses to test the geographic transportability of our models across the sites of each trial (see Supplementary Table S9).

Regression models were trained to predict the absolute change in CDSS scores between baseline and follow-up. Classifiers were trained using two different sets of binary labels:

1. A label indicating a positive or negative change (irrespective of change of magnitude) in CDSS from baseline to the 6- and 12-month follow-up (±ΔCDSS, improvers vs. deteriorators).
2. A classification of patients based on whether they experienced a post-psychotic depressive episode (±PPD) from baseline up to 6 months, with a depressive episode defined according to the CDSS guidelines (CDSS total ≥ 7).

For all regression models, the mean squared error was used as an optimization metric. For ±ΔCDSS classifiers, balanced accuracy (BAC) was used as a metric. To address the high imbalance in sample sizes between the ±PPD groups, we utilized an enhanced BAC metric (see Supplementary Methods).

**Figure 1.**
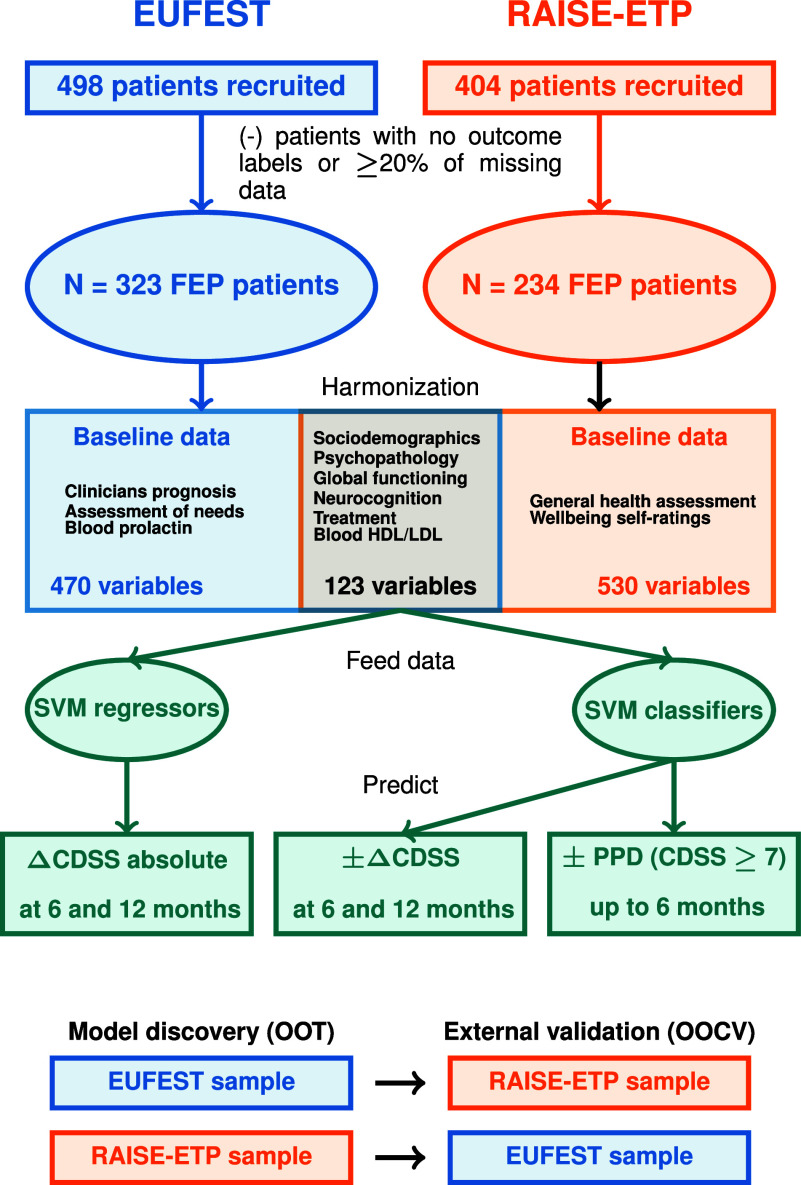
Schematic design of the data processing and analysis design of the study. *Note*: Schematic design of data processing and analysis design. Please refer to the Supplementary Methods for a detailed explanation. Abbreviations: CDSS, Calgary Depression Scale for Schizophrenia; EUFEST, European First-Episode Schizophrenia Trial; FEP, first-episode psychosis; HDL, high-density lipoprotein; LDL, low-density lipoprotein; OOCV, out of cross-validation; OOT, out of training; PPD, post-psychotic depression; RAISE-ETP, Recovery After an Initial Schizophrenia Episode Early Treatment Program; SVM, support vector machine.

Initially, models were trained independently with all the available data on the EUFEST or RAISE-ETP samples to predict ΔCDSS (regression) and ±ΔCDSS (classification) at 6- and 12-month follow-ups, as well as PPD (classification). These models were not externally validated due to the lack of a replication sample with identical variables. To overcome this limitation, we trained and validated the respective new models using only the harmonized variables available in both samples. Specifically, we trained on either EUFEST or RAISE-ETP patients as a discovery sample, reported the out-of-training (OOT) performance, and then validated the respective models in the external cohort using out-of-cross-validation (OOCV). A summary of the data processing and design of the analysis is given in Figure 1. Label permutation analysis (1,000 permutations) at discovery and external validation was conducted to assess the statistical significance of the models using previously described methods (Golland & Fischl, 2003). In addition, models were trained and validated using a condensed set of predictive individual variables with a selection probability over 50% to see if a streamlined tool maintains performance while improving clinical scalability. Models were also retrained without blood-based variables and with only biological variables to evaluate their contribution to predictions.

### Post hoc analyses

To evaluate model prediction consistency, we assessed the correlation of SVM decision scores and classification agreement between OOT and OOCV predictions for the same patients. Receiver operator characteristic (ROC) curves and their area under the curve were used to visually compare model performances. We evaluated the models’ ability to distinguish between negative and depressive symptoms by testing models trained on depressive symptom labels to predict negative symptom labels. We also tested whether PPD models predict positive symptom non-remission to assess whether the pattern underlying depressive episodes also generalizes to non-remission outcomes. This model specificity analysis included significance testing using 1,000 label permutations (see Supplementary Table S11). Model calibration at the OOT and OOCV levels was assessed using calibration curve analysis and measuring the expected calibration error (ECE; see Supplementary Figure S11). In addition, the potential net benefit of utilizing model predictions for treatment decisions, compared to treating all patients or no patients at different decision thresholds, was assessed using decision-curve analysis (see Supplementary Figure S12). To identify robust features, we calculated selection probabilities for models trained on EUFEST and RAISE-ETP samples, and visually compared features selected across both datasets. To evaluate the performance difference of models in the RAISE-ETP patients relative to EUFEST patients, we examined potential differences between both samples using inferential statistics (see Supplementary Methods).

## Results

### Group descriptives and comparisons

Patients across both clinical trials differed significantly in several sociodemographic and psychopathological variables (Supplementary Table S7); RAISE-ETP patients were younger (mean [SD] age: 23.90 [5.27] vs. 25.93 [5.60]), predominantly male (73.50% vs. 56.56%), had a higher body mass index (BMI; 27.06 [6.85] vs. 22.04 [3.17]), lower PANSS positive (18.84 [5.22] vs. 23.34 [6.16]), PANSS general (20.09 [5.30] vs. 44.53 ([10.77]) and CDSS scores (4.16 [3.79] vs. 5.2 [4.85]), and higher rate of antidepressant prescriptions (33.33% vs. 2.18%).

A summary of baseline sociodemographic and clinical statistics of patients grouped by the three classification labels (± ΔCDSS at 6 and 12 months, and ± PPD) is given in Table 1. In RAISE-ETP, +ΔCDSS patients at 6 months had a significantly higher BMI. In addition, +PPD patients were significantly more likely to be female. In EUFEST, +PPD patients had higher PANSS general symptoms and a different diagnostic distribution, with a higher rate of schizoaffective disorder. CDSS scores at baseline significantly differed in patients grouped by all classification labels in both EUFEST and RAISE-ETP samples.

**Table 1.** Differences in baseline variables across all classification labels in patients from the EUFEST sample and the RAISE-ETP sample

Variables	Dataset	+ΔCDSS 6 months	−ΔCDSS 6 months	*t*/*χ* ^2^ (FDR-corrected *p-*value)	+ΔCDSS 12 months	−ΔCDSS 12 months	*t*/*χ* ^2^ (FDR-corrected *p-*value)	+PPD	−PPD	*t*/*χ* ^2^ (FDR-corrected *p*-value)
Sociodemographics										
Sample, *n*	EUFEST	105 (32.81%)	215 (67.18%)	–	92 (28.75%)	228 (71.25%)	–	79 (24.68%)	241 (75.31%)	–
	RAISE-ETP	116 (49.57%)	118 (50.43%)	–	92 (39.32%)	142 (60.68%)	–	45 (19.23%)	189 (80.77%)	–
Age in years, mean (SD)	EUFEST	25.53 (5.59)	26.12 (5.61)	*t*_319_ = −0.98 (0.938)	25.61 (5.52)	26.05 (5.64)	*t*_319_ = −0.64 (0.883)	27.36 (5.90)	25.46 (5.43)	*t*_319_ = 2.54 (0.049)
	RAISE-ETP	24.21 (5.53)	23.58 (5.01)	*t*_233_ = 0.91 (0.572)	23.35 (4.91)	24.25 (5.49)	*t*_233_ = −1.30 (0.588)	24.86 (5.66)	23.67 (5.17)	*t*_233_ = 1.29 (0.296)
Sex (male), *n*	EUFEST	60 (57.14%)	121 (56.28%)	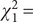 0.001 (1.000)	52 (56.52%)	129 (56.57%)	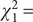 0.000 (1.000)	49 (62.03%)	132 (54.77%)	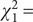 1.00 (0.463)
	RAISE-ETP	78 (67.24%)	94 (43.72%)	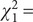 4.02 (0.144)	64 (69.56%)	108 (76.05%)	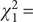 0.90 (0.702)	19 (42.22%)	153 (80.95%)	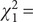 26.04 (<.001)
Employed, *n*	EUFEST	48 (44.44%)	103 (47.91%)	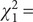 0.06 (1.000)	41 (44.57%)	110 (48.25%)	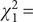 0.22 (0.925)	29 (36.71%)	122 (50.62%)	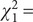 4.08 (0.116)
	RAISE-ETP	17 (14.66%)	14 (11.86%)	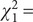 0.19 (0.801)	8 (8.70%)	23 (16.20%)	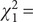 2.12 (0.582)	6 (13.33%)	25 (13.23%)	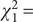 0.000 (1.000)
BMI, mean (SD)	EUFEST	22.23 (3.10)	21.94 (3.20)	*t*_319_ = 0.75 (0.938)	22.25 (3.22)	21.96 (3.15)	*t*_319_ = 0.73 (0.883)	22.55 (3.09)	21.87 (3.17)	*t*_319_ = 1.69 (0.149)
	RAISE-ETP	28.23 (7.70)	25.89 (5.68)	*t*_233_ = 2.61 (0.038)	27.43 (7.18)	26.81 (6.64)	*t*_233_ = 0.66 (0.820)	29.50 (8.62)	26.49 (6.26)	*t*_233_ = 2.16 (0.079)
*Race, n*										
White, *n*	EUFEST	100 (95.24%)	207 (96.28%)	EUFEST: 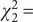 2.23 (0.526) RAISE: 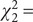 0.37 (1.000)	88 (95.65%)	219 (96.05%)	EUFEST: 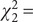 1.27 (0.736) RAISE: 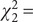 4.41 (0.588)	73 (92.41%)	234 (97.10%)	EUFEST: 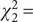 6.60 (0.149) RAISE: 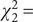 8.96 (0.080)
	RAISE-ETP	69 (55.93%)	66 (55.93%)		50 (54.35%)	85 (59.86%)		23 (51.11%)	112 (59.26%)	
Black, *n*	EUFEST	2 (1.90%)	5 (2.33%)		3 (3.26%)	4 (1.75%)		2 (2.53%)	5 (2.07%)	
	RAISE-ETP	37 (31.90%)	42 (35.59%)		30 (32.61%)	49 (34.51%)		22 (48.89%)	57 (30.16%)	
Asian, *n*	EUFEST	0 (0%)	1 (0.47%)		0 (0%)	1 (0.44%)		1 (1.27%)	0 (0%)	
	RAISE-ETP	2 (1.72%)	2 (1.69%)		3 (3.26%)	1 (0.70%)		0 (0%)	4 (2.12%)	
Psychopathology										
PANSS positive score, mean (SD)	EUFEST	23.21 (6.64)	23.40 (5.93)	*t*_319_ = −0.25 (1.000)	23.86 (6.23)	23.12 (6.14)	*t*_319_ = 0.95 (0.883)	24.33 (5.77)	23.01 (6.27)	*t*_319_ = 1.72 (0.149)
	RAISE-ETP	18.54 (4.91)	19.12 (5.52)	*t*_233_ = −0.86 (0.572)	18.47 (4.96)	19.07 (5.39)	*t*_233_ = −0.89 (0.702)	18.84 (4.53)	18.83 (5.39)	*t*_233_ = 0.01 (1.000)
PANSS negative score, mean (SD)	EUFEST	20.06 (8.00)	21.78 (7.56)	*t*_319_ = −1.83 (0.219)	20.41 (8.11)	21.54 (7.57)	*t*_319_ = − 1.15 (0.805)	22.60 (8.22)	20.75 (7.53)	*t*_319_ = 1.78 (0.149)
	RAISE-ETP	19.49 (5.14)	20.69 (5.40)	*t*_233_ =−1.73 (0.224)	20.04 (5.30)	20.12 (5.31)	*t*_233_ = −0.12 (1.000)	18.80 (4.43)	20.40 (5.45)	*t*_233_ = −2.07 (0.081)
PANSS general score, mean (SD)	EUFEST	40.94 (10.21)	46.28 (10.62)	*t*_319_ = −4.33 (<0.001)	41.02 (9.42)	45.94 (10.97)	*t*_319_ = −4.03 (<0.001)	48.42 (12.99)	43.26 (9.63)	*t*_319_ = 3.25 (0.008)
	RAISE-ETP	35.84 (6.76)	38.63 (7.61)	*t*_233_ = −2.965 (0.017)	36.32 (7.61)	37.84 (7.10)	*t*_233_ = −1.51 (0.582)	38.78 (7.50)	36.88 (7.26)	*t*_233_ = 1.53 (0.230)
CGI, mean (SD)	EUFEST	4.81 (0.83)	4.87 (0.72)	*t*_319_ = −0.63 (0.938)	4.89 (0.80)	4.83 (0.74)	*t*_319_ = 0.596 (0.883)	4.89 (0.83)	4.83 (0.74)	*t*_319_ = 0.46 (0.74)
	RAISE-ETP	4.00 (0.81)	4.11 (0.84)	*t*_233_ = −1.01 (0.553)	4.07 (0.85)	4.06 (0.81)	*t*_233_ = 0.02 (1.000)	4.20 (0.76)	4.03 (0.84)	*t*_233_ = 1.31 (0.296)
CDSS, mean (SD)	EUFEST	1.27 (2.00)	7.12 (4.67)	*t*_319_ = −15.62 (<0.001)	0.98 (2.16)	6.90 (4.58)	*t*_319_ = −15.68 (<0.001)	9.18 (5.46)	3.89 (3.81)	*t*_319_ = 8.00 (<0.001)
	RAISE-ETP	2.48 (3.09)	5.82 (3.69)	*t*_233_ = −7.50 (<0.001)	2.04 (2.68)	5.54 (3.77)	*t*_233_ = −8.28 (<0.001)	5.95 (4.05)	3.74 (3.61)	*t*_233_ = 3.36 (0.010)
Substance use										
Alcohol use, *n*	EUFEST	35 (33.33%)	76 (35.35%)	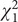 = 0.05 (1.000)	29 (31.52%)	81 (35.53%)	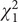 = 0.39 (0.883)	30 (37.97%)	81 (33.61%)	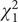 = 0.33 (0.698)
	RAISE-ETP	37 (31.89%)	26 (22.03%)	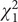 = 2.41 (0.275)	23 (25%)	40 (28.17%)	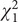 = 0.15 (0.863)	14 (31.11%)	49 (25.93%)	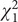 = 0.27 (0.806)
Cannabis use, *n*	EUFEST	24 (22.86%)	47 (21.86%)	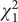 = 0.003 (1.000)	20 (21.74%)	51 (22.37%)	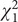 = 0.000 (1.000)	21 (26.58%)	50 (20.75%)	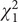 = 0.86 (0.471)
	RAISE-ETP	28 (24.14%)	25 (21.19%)	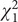 = >0.15 (0.801)	24 (26.09%)	29 (20.42%)	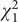 = 0.73 (0.70)	10 (22.22%)	43 (22.75%)	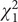 = 0.000 (1.000)
Other substance use, *n*	EUFEST	10 (9.52%)	17 (7.91%)	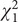 = 0.07 (1.000)	8 (8.70%)	19 (8.33%)	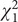 = 0.000 (1.000)	8 (10.13%)	19 (7.88%)	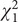 = 0.15 (0.743)
	RAISE-ETP	2 (1.74%)	3 (2.54%)	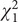 = 0.000 (1.000)	1 (1.09%)	4 (2.82%)	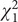 = 0.18 (0.863)	1 (2.22%)	4 (2.12%)	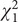 = 0.000 (1.000)
Diagnosis										
Diagnose, *n*										
Schizophrenia, *n*	EUFEST	56 (53.3%)	107 (49.77%)	EUFEST: 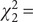 9.79 (0.082) RAISE-ETP: 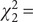 4.28 (0.466)	47 (51.09%)	116 (50.88%)	EUFEST: 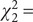 8.42 (0.152) RAISE-ETP: 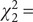 1.81 (0.863)	38 (48.10%)	125 (51.87%)	EUFEST: 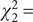 10.36 (0.050) RAISE-ETP: 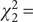 9.81 (0.071)
	RAISE-ETP	73 (62.93%)	61 (51.69%)		55 (59.78%)	79 (55.63%)		23 (51.11%)	111 (58.73%)	
Schizophreniform disorder, *n*	EUFEST	0 (0%)	0 (0%)		0 (0%)	0 (0%)		0 (0%)	0 (0%)	
	RAISE-ETP	16 (13.79%)	21 (17.80%)		15 (16.30%)	22 (15.49%)		3 (6.66%)	34 (17.98%)	
Schizoaffective disorder, *n*	EUFEST	1 (0.97%)	23 (10.85%)		1 (1.11%)	23 (15.19%)		12 (15.19%)	12 (5.08%)	
	RAISE-ETP	15 (12.93%)	25 (21.19%)		12 (13.04%)	28 (19.72%)		14 (31.11%)	26 (13.76%)	
Other psychotic disorder, *n*	EUFEST	46 (44.66%)	82 (38.68%)		42 (46.66%)	86 (38.22%)		29 (36.71%)	99 (41.95%)	
	RAISE	12 (10.34%)	11 (9.32%)		10 (10.87%)	13 (9.15%)		5 (11.11%)	18 (9.52%)	
Medication										
Antidepressant, *n*	EUFEST	2 (1.90%)	5 (2.33%)	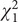 = 0.000 (1.000)	2 (2.17%)	5 (2.19%)	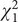 = 0.000 (1.000)	2 (2.53%)	5 (2.07%)	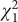 = 0.000 (1.000)
	RAISE	41 (35.34%)	37 (31.36%)	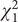 = 0.26 (0.801)	31 (33.70%)	47 (33.10%)	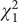 = 0.000 (1.000)	22 (48.89%)	56 (29.62%)	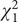 = 5.23 (0.071)

Abbreviations: BMI, body mass index (calculated as weight in kilograms divided by square of height in meters); CDSS, Calgary Depression Scale for Schizophrenia; CGI, clinical global impression; EUFEST, European First-Episode Schizophrenia Trial; PANSS, Positive and Negative Syndrome Scale; RAISE-ETP, Recovery After an Initial Schizophrenia Episode Early Treatment.

### Machine learning analyses

**Figure 2.**
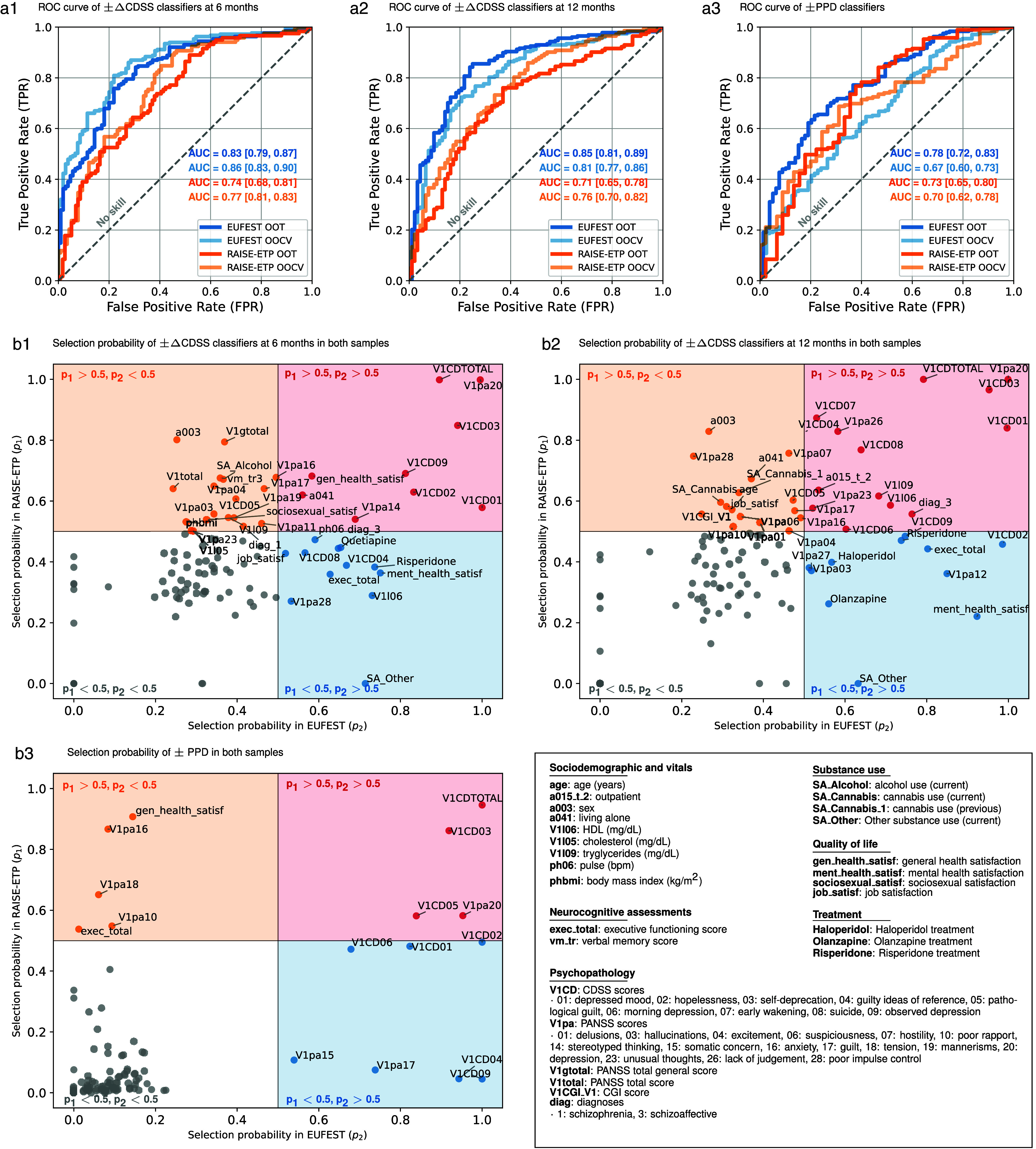
Predictive performance and predictive features of ±ΔCDSS and ±PPD classifiers. *Note*: The performance of classifiers trained using the EUFEST or RAISE-ETP samples was evaluated at the OOT and OOCV levels using ROC curve analysis. This analysis was conducted for models predicting the ±ΔCDSS label at 6 months (a1), at 12 months (a2), and the ±PPD label (a3). In addition, we assessed the robustness of the selected features by visualizing the selection probability of each harmonized feature when the model was trained with either cohort for the ±ΔCDSS label at 6 months (b1), at 12 months (b2), and the ±PPD label (b3). Abbreviations: AUC, area under the curve; CDSS, Calgary Depression Scale for Schizophrenia; CGI, clinical global impression; EUFEST, European First-Episode Schizophrenia Trial; OOCV, out of cross-validation; OOT, out of training; PANSS, Positive and Negative Syndrome Scale; PPD, post-psychotic depression; ROC, receiver operating characteristic; RAISE-ETP, Recovery After an Initial Schizophrenia Episode Early Treatment Program. Note: The EUFEST OOCV performance corresponds to models trained on RAISE-ETP and validated on EUFEST, whereas the EUFEST OOT performance reflects the performance of models trained and evaluated within the EUFEST cohort. Similarly, the RAISE-ETP OOCV performance pertains to models trained on EUFEST and validated on RAISE-ETP, while the RAISE-ETP OOT performance pertains to models trained and evaluated within the RAISE-ETP cohort.

Initially, we utilized all the available variables in the EUFEST and RAISE-ETP samples independently to train the linear SVMs (see Supplementary Results). Then, we reran our SVM regression and classification training pipelines with the harmonized 123 variables available across both samples. We trained the models using the EUFEST as a discovery sample and applied the models to RAISE-ETP as an OOCV sample, and vice versa. The performance metrics (OOT and OOCV) for the two regression and three classification labels are shown in Table 2. Label permutation testing (see Supplementary Methods) was used to test the significance of model predictions. EUFEST-trained classifiers predicted ±ΔCDSS at the 6-month follow-up with an OOT-BAC of 75.09% [2.31] (*P* < .001, ECE = 12%) and an OOCV-BAC of 66.26% (*P* < .001, ECE = 6%), at the 12-month follow-up with an OOT-BAC of 77.61% (*P* < .001, ECE = 14%) and an OOCV-BAC of 68.78% (*P* < .001, ECE = 11%), and ±PPD with an OOT-BAC of 69.01% (*P* < .001, ECE = 13%) and an OOCV-BAC of 59.15% (*P* < .001, ECE = 15%). RAISE-ETP-trained classifiers predicted ± ΔCDSS at the 6-month follow-up with an OOT-BAC of 66.26% (*P* < .001, ECE = 6%) and an OOCV-BAC of 76.35% (*P* < .001, ECE = 15%), at the 12-month follow-up with an OOT-BAC of 67.88% (*P* < .001, ECE = 11%) and an OOCV-BAC of 73.05% (*P* = 0.026, ECE = 13%), and ±PPD with OOT BAC of 67.67% (*P* < .001, ECE=12%) and an OOCV-BAC of 58.92% (*P* < .001, ECE = 12%). Thus, the discriminative performance of the ±ΔCDSS models was consistently higher in the EUFEST sample compared to the RAISE-ETP sample, independently of whether the EUFEST sample was used as a discovery or validation sample. This effect was not observed in models predicting PPD. In addition, the accuracy of models predicting ±ΔCDSS was higher at the 12-month follow-up. These higher performances are evident in the ROC curves shown in Figure 2a. Decision-curve analysis of models at the OOT and OOCV level (Supplementary Figure S12) shows that models predicting ±ΔCDSS provide a clear net benefit at higher threshold probabilities. With the ±PPD label, the net benefit is only evident when models are trained in the EUFEST cohort.

**Table 2.** Performance metrics of regressors and classifiers in discovery and out-of-sample validation

Sample	Label	Regressors		Classifiers											
		*r* (*p*-value)	*R* ^2^	TP	TN	FP	FN	Sens.	Spec.	BAC	PPV	NPV	PSI	AUC-ROC	Perm. *p*-value
EUFEST OOT	± ΔCDSS 6 months	0.82 (<.001)	66.92	82	155	60	23	78.10	72.09	75.09	57.75	87.08	44.83	0.83	<.001
	± ΔCDSS 12 months	0.89 (<.001)	78.45	73	173	55	19	79.35	75.88	77.61	57.03	90.10	35.90	0.76	<.001
	± PPD	–	–	52	174	67	27	65.82	72.20	69.01	43.70	86.57	30.26	0.78	<.001
EUFEST OOCV	± ΔCDSS 6 months	0.78 (<.001)	60.27	90	144	71	15	85.71	66.98	76.35	55.90	90.57	46.47	0.86	<.001
	± ΔCDSS 12 months	0.83 (<.001)	69.35	63	177	51	29	68.48	77.63	73.05	55.26	85.92	41.18	0.82	0.026
	± PPD	–	–	39	165	76	40	49.37	68.46	58.92	33.91	80.49	14.40	0.67	<.001
RAISE-ETP OOT	± ΔCDSS 6 months	0.54 (<.001)	29.28	79	76	42	37	68.10	64.41	66.26	65.29	67.26	32.55	0.74	<.001
	± ΔCDSS 12 months	0.58 (<.001)	33.06	64	94	48	28	69.57	66.20	67.88	57.14	77.04	34.19	0.71	<.001
	± PPD	–	–	29	134	55	16	64.44	70.90	67.67	34.52	89.33	23.86	0.73	<.001
RAISE-ETP OOCV	± ΔCDSS 6 months	0.49 (<.001)	24.37	79	76	42	37	68.10	64.41	66.26	65.29	67.26	32.55	0.77	<.001
	± ΔCDSS 12 months	0.60 (<.001)	35.65	65	95	47	27	70.65	66.90	68.78	58.03	77.87	35.90	0.76	<.001
	± PPD	–	–	18	148	41	27	40.0	78.31	59.15	30.51	84.57	15.08	0.70	<.001

Abbreviations: AUC-ROC, area under the receiver operating characteristic curve; BAC, balanced accuracy; EUFEST, European First-Episode Schizophrenia Trial; FN, false negative; FP, false positive; NPV, negative predictive value; OOCV, out of cross-validation; OOT, out of training; PPV, positive predictive value; PSI, Prognostic Summary Index; RAISE-ETP, Recovery After an Initial Schizophrenia Episode Early Treatment Program; sens, sensitivity; spec, specificity; TN, true negative; TP, true positive.

*Note*: The EUFEST OOCV performance corresponds to models trained on RAISE-ETP and validated on EUFEST, whereas the EUFEST OOT performance reflects the performance of models trained and evaluated within the EUFEST cohort. Similarly, the RAISE-ETP OOCV performance pertains to models trained on EUFEST and validated on RAISE-ETP, while the RAISE-ETP OOT performance pertains to models trained and evaluated within the RAISE-ETP cohort.

Significant predictors and effect direction were determined by the feature selection probability and the cross-validated ratio (*p* > 0.5 and CVR±3; see Supplementary Methods). Overall, severity of psychopathology (PANSS and CDSS scores), mental and physical health satisfaction, job satisfaction, living alone, blood cholesterol and triglycerides, pulse, substance use, neurocognitive performance (executive functioning), haloperidol treatment, and diagnosis of schizoaffective disorder were selected by the regression and classification models to predict depressive symptoms (Supplementary Figures S5–S8).

To test the specificity of our models for depressive outcomes, we assessed whether models trained on depressive symptoms also predicted changes in negative symptoms and positive symptom non-remissions (Supplementary Table S11). To assess the clinical applicability of these predictors, each model was retrained with a condensed variable set by using variables that were individual items (not total scores) and had a selection probability of 50% or higher. We statistically compared the predictions of models using the condensed set and all harmonized variables (Supplementary Table S18) and observed that BAC was consistent for both. In addition, we examined the consistency of feature selection independently across the two samples for models trained separately in each cohort (Figure 2b and Supplementary Figure S9). Severity of psychopathology scores (PANSS and CDSS scores), general health satisfaction, living alone, blood cholesterol, and triglycerides were consistently selected irrespective of the training sample. Finally, we evaluated the consistency of model predictions and assessed the contribution of biological variables and history of depression (in the EUFEST sample) to model predictions (see Supplementary Results).

### Misclassification analyses

**Figure 3.**
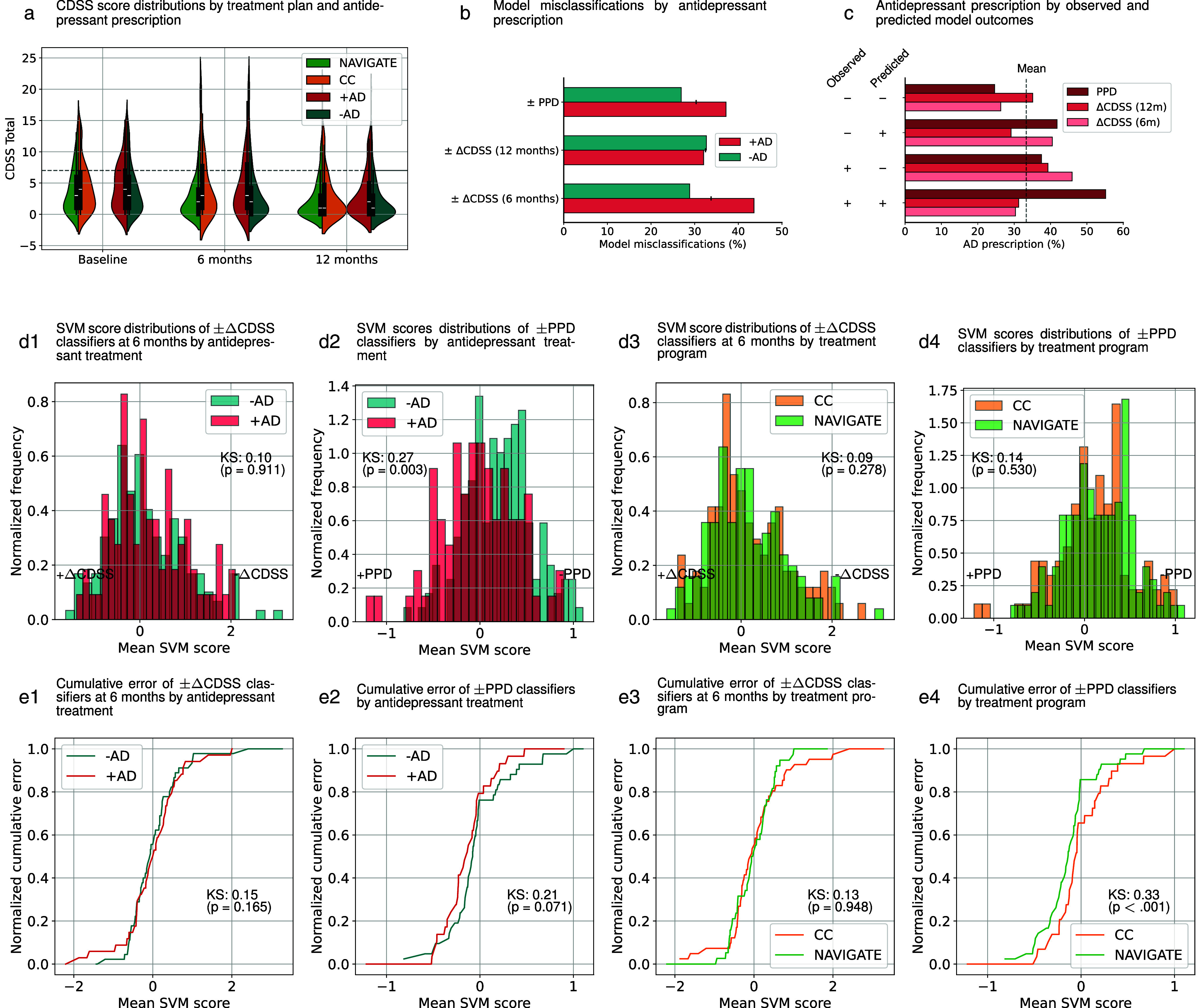
Comparison of CDSS scores, models’ decision scores, and misclassifications for patients in the NAVIGATE and community-based treatment, and by antidepressant prescription. *Note*: (a) In the RAISE-ETP sample, we compared the trajectories of CDSS scores for patients in the NAVIGATE treatment program (NAVIGATE), the community-based care (CC), without an antidepressant prescription at baseline (-AD), and with an antidepressant prescription at baseline (+AD). In addition, (b) we compared classifier misclassifications by antidepressant prescription and (c) the percentage of antidepressant prescriptions by type of misclassification. Furthermore, we compared the distributions of SVM decision scores of classifiers by antidepressant prescription and treatment plan for the ±ΔCDSS label at 6 months (d1 and d3), at 12 months (see Supplementary Figure S14), and the ±PPD label (d2 and d4). To study the type of misclassifications by treatment, we compared the cumulative misclassifications by antidepressant prescription and treatment plan for the ±ΔCDSS label at 6 months (e1 and e3), at 12 months (see Supplementary Figure S15), and the ±PPD label (e2 and e4). Abbreviations: CDSS, Calgary Depression Scale for Schizophrenia; KS, Kolmogorov–Smirnov statistic.

We studied the lower predictive performance of our models in the RAISE-ETP patients. To this end, we first studied the influence of treatment assignment and antidepressant prescription at baseline on depression courses in RAISE-ETP (see Supplementary Results). We then evaluated the interactions between treatment arm, antidepressive prescription, and model misclassification. We examined the trajectories of CDSS scores stratified by treatment program (CC and NAVIGATE), and antidepressant treatment (±AD; Figure 3a). We identified higher model misclassification in patients with a prescribed antidepressant for the ±ΔCDSS (6 months) label and trending toward significance for the ±PPD label (Figure 3b). For the ±PPD label, antidepressant prescription was higher for patients with a predicted and/or observed poor outcome (Figure 3c). For ±ΔCDSS (6 months) labels, patients with a predicted good but observed bad outcome, and vice versa, were more likely to be prescribed antidepressants. We calculated the SVM score distributions, cumulative distributions, and cumulative model errors for patients with and without antidepressant prescriptions and in either treatment program (Figure 3d,e and Supplementary Figures S14 and S15). Models largely did not discriminate between patients with different treatments, except for the ±PPD model, which was more likely to predict PPD in patients with a prescribed antidepressant. In addition, the cumulative error analysis of decision scores showed that for the ±PPD label, the NAVIGATE program had significantly more misclassifications in patients where a negative outcome was predicted (+PPD) but a positive outcome was observed (−PPD; Figure 3e4). A similar effect was seen in patients with a prescribed antidepressant (Figure 3e2), although in this case, the differences in error distributions trended toward significance.

## Discussion

Using robust cross-validation, we built linear SVM models to predict changes in depressive symptoms (±ΔCDSS) at 6 and 12 months and depressive episodes (±PPD) in the first 6 months in FEP patients. Models trained on EUFEST and RAISE-ETP data predicted depressive symptoms with high sensitivity and specificity, and depressive episodes with moderate specificity but low sensitivity. Decision-curve analysis showed that the clinical applicability of the models would be best optimized at higher thresholds, targeting patients predicted to have the poorest outcomes as defined by depressive symptoms. Key predictors included baseline symptoms, neurocognitive performance, quality of life, and blood markers (e.g. prolactin in EUFEST and alanine transferase in RAISE-ETP). A harmonized subset of variables (*n* = 123) was used to retrain models, achieving consistent predictive performance across datasets. Harmonized features, such as symptom severity, quality of life, and blood markers (triglycerides and cholesterol), were validated in both samples. Cross-validation between EUFEST and RAISE-ETP models showed robust geographic transportability of predictive signatures between multiple European countries with highly different healthcare systems and the United States. These signatures were specific for depressive symptoms (CDSS), and not PANSS-based negative symptoms or positive non-remission. Finally, we demonstrated that the models maintain their predictive performance when using a highly condensed set of top predictors.

These results have potential clinical implications. First, the findings validate recently developed risk prediction models showing the high prognostic value of baseline symptom severity and history of depression for the prediction of future depressive episodes, both in the context of comorbid psychosis (Banerjee et al., 2024; Carter et al., 2025) and in major depressive disorder independently (Song et al., 2023; Teutenberg et al., 2025). Second, the consistent model selection of cognitive deficits and poor quality of life metrics shows the relevance of the patients’ baseline functioning in predicting future depression-related outcomes. Cognitive deficits are a core feature of psychosis and significantly contribute to the impaired functioning of patients with psychosis, and although not extensively studied, it is known that affective symptoms in psychosis are often associated with more pronounced cognitive deficits (McCutcheon, Keefe, & McGuire, 2023). In addition, comorbid depression and general psychopathology have long been associated with lower quality of life and the subjective perception of poorer mental and general health (Watson et al., 2018), suggesting that a poorer perception of health and quality of life may be influenced by depressive mood in psychosis, and vice versa (Narvaez, Twamley, McKibbin, Heaton, & Patterson, 2008).

Some biological markers were also consistently selected in both samples (triglycerides and cholesterol blood levels) and independently (prolactin levels in EUFEST and BMI and alanine transaminase levels in RAISE-ETP), adding a key biological dimension to the models’ prediction of depressive symptoms. Excluding these markers increased models’ bias toward predicting the predominant group (patients in remission from depression), evidenced by the higher imbalance between sensitivity and specificity. This suggests that simple blood-based biological variables are necessary to ensure the unbiased and accurate detection of depressive symptom changes in FEP when combined with clinical and quality-of-life predictors. Patients with psychiatric disorders demonstrate immuno-metabolic dysfunction and metabolic comorbidity compared to the normal population (Han, 2022; Peng, Lin, Lee, & Chen, 2024). This association can arise due to antipsychotic side effects (Mondelli et al., 2013). However, previous studies have shown metabolic impairment in drug-naïve first-episode patients (Garrido-Torres et al., 2021), and predate illness and prodromal onset, potentially resulting from shared pro-inflammatory predisposition (Palmer et al., 2024). In the context of comorbid depression and psychosis, serum lipids and prolactin have also been found to be associated with affective symptoms (Gohar et al., 2019). In keeping with the previous literature, the results of this work indicate that cost-effective monitoring of abnormal lipid and metabolic hormonal levels may provide a valuable biomarker for predicting and managing depressive symptoms in FEP patients. In addition, trying to incorporate lifestyle and psychosocial interventions in first-episode patients might have an impact on the prevalence of depressive symptoms in psychosis (Schmitt et al., 2018).

Importantly, we observed that models predicting depressive symptom trajectories (ΔCDSS) performed better in EUFEST than in RAISE-ETP, both at the OOT and OOCV levels. Baseline comparisons revealed significant differences between the cohorts in sociodemographics, psychopathology, and antidepressant prescriptions. Notably, RAISE-ETP in the NAVIGATE program (Mueser et al., 2015) showed a sharper decline in depressive symptoms. The same effect was also observed in patients without an antidepressant prescription. Since antidepressant prescriptions were not randomized, unlike the treatment program, this effect might be an indication of bias reflecting clinicians’ tendency to prescribe antidepressants to patients they predict to be at higher risk of poor affective outcomes. Patients in the NAVIGATE program were generally prescribed fewer antidepressants, indicating a confounding effect between prescriptions and treatment plans. Interaction analyses revealed a trend, indicating that NAVIGATE was more effective in reducing depressive symptoms among patients prescribed antidepressants at baseline. Model misclassifications for the PPD label were notably higher at negative SVM decision scores for NAVIGATE-treated patients receiving antidepressants. Specifically, these errors involved predicting negative outcomes (+PPD) when positive outcomes (−PPD) were observed. This suggests that these interventions effectively reduce the likelihood of poor outcomes, thus improving treatment results for patients who received a poor prognosis by our models. The positive effect of psychosocial interventions on depressive symptoms in FEP was observed by the RAISE-ETP group (Kane et al., 2015a) and other trials (Hagen, Nordahl, & Gråwe, 2005; Ruggeri et al., 2015). In addition, growing evidence suggests that the coadministration of antidepressants is effective in the treatment of depressive symptoms in FEP (Barnes et al., 2019; Dondé, Vignaud, Poulet, Brunelin, & Haesebaert, 2018). Despite this evidence, these additional therapies are not standard recommendations in clinical practice (Dondé et al., 2018). Various international treatment guidelines recommend waiting for primary antipsychotics to take effect during the acute phase of the illness, since some FEP patients will remit from depressive symptoms with only antipsychotic medication. Recently published guidelines, including the British Association of Psychopharmacology, suggest that coadministration of antidepressants might be justified but should be considered after careful assessment of the risks of polypharmacy (Barnes et al., 2019). This work adds weight to the body of evidence supporting both integrative talking therapies and antidepressants for the treatment of depressive symptoms in FEP. Furthermore, it introduces a potential risk prediction tool that could identify those patients unlikely to remit with antipsychotic medication alone, complementing clinicians’ optimism bias in predicting poor mental health outcomes (Koutsouleris et al., 2021). Ultimately, by identifying patients who are at risk for prolonged depressive symptoms early on, this tool has the potential to improve treatment trajectories, reduce the burden of illness, and enhance patients’ quality of life by preventing periods of insufficient treatment and associated comorbidities.

### Limitations

This work has limitations. First, we restricted the analysis to patients in EUFEST and RAISE-ETP who attended the follow-ups used as outcome data. Although we found no baseline differences in included and excluded patients, this decreased the sample size of our analysis to 323 EUFEST patients and 234 RAISE-ETP patients. While we employed robust nested cross-validation procedures, the moderate sample size may still pose a risk of overfitting. In addition, patients who attended the follow-ups may represent a more engaged subgroup of patients, potentially introducing a selection bias. Furthermore, although multinational and multisite, the EUFEST and RAISE-ETP are largely Western samples, which may limit the generalizability of our findings to non-Western populations. While no significant differences in model misclassifications were observed across racial and ethnic groups in the more diverse RAISE-ETP sample (see Supplementary Table S17), further research is necessary to validate these results in broader, more representative populations. Additionally, we used CDSS scores to identify depressive episodes in psychosis, with the limitation that CDSS questionnaire is not a diagnostic interview; a score above 7 suggests moderate to severe depression within 2 weeks and has an 82% specificity and 85% sensitivity for predicting the presence of a major depressive episode in schizophrenia. This limitation should be noted, as the CDSS may not fully reflect the intricacies of a depression diagnosis.

## Conclusion

The results of this study demonstrated and independently validated that depressive symptoms and episodes are predictable from baseline information following psychosis onset. Notably, our study also demonstrated that certain concomitant treatments, such as the NAVIGATE treatment program and antidepressants, may be useful in the treatment of depressive symptoms in FEP. Given that integrative treatments require significant resources and a careful assessment of their benefit-risk ratios in the individual patient, the clinical algorithms presented here could serve as scalable stratification tools for the early identification of patients at the highest risk of impactful depressive symptoms and episodes following a conventional treatment for psychosis. Future research should focus on refining these models, validating them at different stages of psychosis, exploring their use with diverse data sources – such as electronic health records or genetic data – and investigating the causal mechanisms underlying depressive symptoms in psychosis.

## Supporting information

Mena et al. supplementary material 1 Mena et al. supplementary material

Mena et al. supplementary material 2 Mena et al. supplementary material

## Supplementary material

The supplementary material for this article can be found at http://doi.org/10.1017/S0033291725100950.
