# Supplementary material for: AI-based prediction of depression symptomatology in first-episode psychosis patients: insights from the EUFEST and RAISE-ETP clinical trials
Source: Psychol Med. 2025 Jul 30;55:e221. doi: 10.1017/S0033291725100950 (PMC12341035; doi:10.1017/S0033291725100950)
Supplement: Mena et al. supplementary material 1 — Mena et al. supplementary material [file S0033291725100950sup001.docx]

**Supporting Information for**

**AI-based prediction of depression symptomatology in first episode psychosis patients: insights from the EUFEST and RAISE-ETP clinical trials**

**Sergio Mena^1^; Fiona Coutts^1^; Jana von Trott^1,2^; Esin Ucur,^1^; Clara Vetter^2^; René R. Kahn^3,4^; W. Wolfgang Fleischhacker^5^; John M. Kane^6^; Oliver D. Howes^1^; Rachel Upthegrove^7,8^; Paris A. Lalousis^1,2*^; Nikolaos Koutsouleris^1,2,9,10*^**

^1^ Department of Psychosis Studies, Institute of Psychiatry, Psychology and Neuroscience, King’s College London, London, United Kingdom.

^2^ Department of Psychiatry and Psychotherapy, Ludwig-Maximilians-University, Munich, Germany.

^3^ Department of Psychiatry, Icahn School of Medicine at Mount Sinai, New York, NY, USA.

^4^ Department of Psychiatry, University Medical Center Utrecht, Utrecht, the Netherlands.

^5^ Department of Biological Psychiatry, Medical University Innsbruck, Anichstrasse 35, A-6020 Innsbruck, Austria.

^6^ The Zucker Hillside Hospital, Psychiatry Research, Northwell Health, Glen Oaks, NY, USA.

^7^ Department of Psychiatry, University of Oxford, United Kingdom.

^8^ Institute for Mental Health, School of Psychology, University of Birmingham, United Kingdom.

^9^ Max Planck Institute of Psychiatry, Munich, Germany.

^10^ German Center for Mental Health (DZPG), Berlin, Germany.

Corresponding author: Nikolaos Koutsouleris

Email: [nikolaos.koutsouleris@kcl.ac.uk](mailto:nikolaos.koutsouleris@kcl.ac.uk)

**This PDF file includes:**

**Supplementary Methods**

**Supplementary Results**

**Supplementary References**

**Tables**

Table S1. Data domains and collection times in the EUFEST sample.

Table S2. Data domains and collection times in the RAISE-ETP sample.

Table S3. Harmonization of variables across both datasets.

Table S4. Battery of harmonized variables and value ranges.

Table S5. Statistical comparisons of baseline variables between EUFEST patients included and excluded in the study.

Table S6. Statistical comparisons of baseline variables between RAISE-ETP patients included and excluded in the study.

Table S7. Statistical comparisons of variables between patients included in the study from EUFEST and RAISE-ETP samples.

Table S8. Performance metrics of regressors and classifiers using all available variables at baseline.

Table S9. Performance metrics of regressors and classifiers in discovery and out of sample validation using leave-site-out cross-validation.

Table S10. Performance metrics of regressors and classifiers in discovery and out of sample validation without psychopathology questionnaire total scores.

Table S11. Performance metrics of classifiers in the prediction of negative symptoms changes and remission from psychosis.

Table S12. Mixed-design ANOVA of depression scores by treatment group and visit in the RAISE-ETP sample.

Table S13. Mixed-design ANOVA of depression scores by antidepressant treatment and visit in the RAISE-ETP sample.

Table S14. Mixed-design ANOVA of depression scores by antidepressant type and visit for AD treated patients in RAISE-ETP sample.

Table S15. Mixed linear model regression of CDSS scores considering antidepressant prescription, treatment plan and long-acting injectable prescription.

Table S16. Mixed linear model regression of CDSS scores considering antidepressant prescription, treatment plan and long-acting injectable prescription for patients CDSS total ≥ 7 at baseline.

Table S17. Comparison of misclassifications across different ethnicities and races in RAISE-ETP patients.

Table S18. Comparison of models’ performance when trained with all harmonized variables and a condensed set of top predictors.

Table 19. Performance metrics of regressors and classifiers in discovery and out of sample validation using only biological variables.

Table S20. Performance metrics of regressors and classifiers in discovery and out of sample validation using all harmonized variables but excluding biological variables.

Table S21. Comparison of models’ performance when trained with all the available harmonized variables and when removing the biological variables.

**Figures**

Figure S1. Top 25 predictors of regressors trained with all available variables in the EUFEST sample.

Figure S2. Top 25 predictors of classifiers trained with all available variables in the EUFEST sample.

Figure S3. Top 25 predictors of regressors trained with all available variables in the RAISE-ETP sample.

Figure S4. Top 25 predictors of classifiers trained with all available variables in the RAISE-ETP sample.

Figure S5. Top 25 predictors of regressors trained with harmonized variables in the EUFEST sample.

Figure S6. Top 25 predictors of classifiers trained with harmonized variables in the EUFEST sample.

Figure S7. Top 25 predictors of regressors trained with harmonized variables in the RAISE-ETP sample.

Figure S8. Top 25 predictors of classifiers trained with harmonized variables in the RAISE-ETP sample

Figure S9. Selection probability of features across ΔCDSS regressors in both samples.

Figure S10. Correlation between SVM out-of-training and out-of-cross-validation scores for EUFEST and RAISE-ETP patients.

Figure S11. Reliability diagrams of the classifiers.

Figure S12. Decision curves of classifiers.

Figure S13. Calgary depression scores by treatment type in the RAISE-ETP sample.

Figure S14. Decision score distributions and cumulative error distributions by antidepressant use in RAISE-ETP patients

Figure S15. Decision score distributions and cumulative error distributions by treatment plan in RAISE-ETP patients.

**1 Supplementary Methods**

- 1. **Study design**

Data was extracted from the European First Episode Schizophrenia Trial (EUFEST) and Recovery After an Initial Schizophrenia Episode Early Treatment Program (RAISE-ETP) clinical trials. Each patient, participant, caregiver, or legal representative provided written informed consent. The study designs of the clinical trials are detailed in previous publications (1,2). We will briefly describe both studies here. The EUFEST trial recruited 498 patients between December 23, 2002, and January 14, 2006, aged 18-40, who met the DSM-IV criteria for schizophrenia, schizoaffective disorder, or schizophreniform disorder. These patients were sourced from 50 mental health centres across 14 European countries and Israel. Eligibility required limited prior use of antipsychotics (no more than two weeks in the past year or six weeks in total). Participants were randomly assigned to one year of treatment with either low-dose haloperidol (n = 103), amisulpride (n = 104), olanzapine (n = 105), quetiapine (n = 104), or ziprasidone (n = 82). The primary outcome of the trial was treatment retention, while secondary outcomes assessed various psychopathological dimensions, including the Calgary Depression Scale for Schizophrenia (CDSS), side effects, treatment adherence, quality of life, social needs, substance use, and cognitive function. Measurements were conducted at 4 weeks, 3 months, 6 months, and 12 months after baseline. Blood samples markers included fasting insulin and glucose, prolactin, high-density lipoprotein (HDL) and low-density lipoprotein (LDL) cholesterol, and triglycerides.

The RAISE-ETP, available from the National Institute of Mental Health Data Archives, was a cluster-randomized trial involving 34 sites across 21 U.S. states. Recruitment started on July 10 and ended on July 12, with 404 patients aged 15-40 enrolled, all meeting DSM-IV criteria for schizophrenia, schizoaffective disorder, schizophreniform disorder, psychotic disorder not otherwise specified, or brief psychotic disorder. Eligibility also required patients to have experienced only one psychotic episode and to have had limited prior antipsychotic use (no more than 6 months). Treatment sites were randomly assigned to either usual community care (n = 17) or the NAVIGATE treatment program (n = 17)(3), which comprised coordinated interventions such as personalized medication using a computer decision-support system, family psychoeducation, resilience-focused individual therapy, and supported education/employment. In the RAISE-ETP study, the primary outcomes measured included the Quality of Life Scale (QLS) and various dimensions of psychopathology, including depressive symptoms measured by the CDSS assessment. Additionally, the study monitored medication adherence, health and vital signs, substance use, side effects, patient self-ratings on well-being and recovery, neurocognitive performance, family assessments, and study compliance, alongside baseline demographics and study participation details. Blood samples taken at baseline included sodium, potassium, chloride, CO2, glucose, creatinine, urea nitrogen, total protein, albumin, total bilirubin, alkaline phosphatase, aspartate transferase (AST), alanine transaminase (ALT), calcium, HDL, LDL, triglycerides, hemoglobin A1c, and fasting insulin, and anion gap test. Detailed data domains measured and their corresponding collection dates for both trials can be found in **Table S1** and **Table S2**. Clinical and neurocognitive questionnaires were administered by experienced clinicians such as psychiatrists, psychologists or trained clinical researchers with expertise in mental health assessment.

- 1. **Pre-processing of variables**

In the EUFEST study, we restricted the study to 320 patients with available outcomes at the 4-week, 6-month, and 12-month visits. For the RAISE-ETP study, we included 234 patients with outcomes available at the 6-month and 12-month visits. In both studies, patients were excluded if 20% or more of the baseline variables were missing. No significant differences in baseline characteristics were observed between included and excluded patients in either sample (see **Table S5** and **Table S6**). The data extraction pipeline consisted of first extracting the baseline variables to form our prediction battery (the data used by the model to predict the patient outcomes), and then extract the outcome variables from future visits (the variables that the model is trained to predict). We extracted 470 baseline variables from the EUFEST dataset and 530 from the RAISE-ETP dataset, covering clinical, cognitive, functioning, and quality-of-life assessments. All variables were adapted for machine learning analyses; categorical variables were dummy-coded, “not known” and “not applicable” entries were converted into missing values and event-based variables (e.g., hospitalizations, interventions and medication) were converted to binary variables (e.g., absence or presence of hospitalizations before baseline). An exhaustive description of variables, their value range and transformations applied are given in the supplementary files below. Finally, we calculated relevant total scores of assessments (e.g., PANSS positive, negative and general scores) and added them to the prediction battery. Next, we selected variables available in both clinical trials and harmonized variables with a similarity of ≥70% using the Harmony online tool (4). For neurocognitive and quality-of-life assessments, we harmonized items pertaining to the same constructs or domains. IPython scripts used to extract the data from the raw files and process them into machine learning ready files are given in the supplementary files:

- **Data dictionary EUFEST.xlsx**: Description of all the EUFEST baseline predictors and the adaptations applied.
- **Data dictionary RAISE.xlsx**: Description of all the RAISE-ETP baseline predictors and the adaptations applied.
- **baseline_extraction_eufest.html**: extraction of baseline variables in the EUFEST cohort.
- **baseline_extraction_raiseetp.html**: extraction of baseline variables in the RAISE-ETP cohort.
- **prediction_set_extraction_eufest.html**: extraction of outcome variables in the EUFEST cohort.
- **prediction_set_extraction_raiseetp.html**: extraction of outcome variables in the RAISE-ETP cohort.

**1.3 Harmonization of variables**

To perform out-of-sample external validation of the model predictions, we selected variables present in both the EUFEST and RAISE-ETP samples, harmonizing those that covered analogous information domains. We used the Harmony online tool (4), and harmonized the variables with a similarity level of 70% or higher. Variables that required harmonization are covered in **Table S3**. The Calgary Depression Scale for Schizophrenia, the Positive and Negative Syndrome Scale, Clinical Global Impression, and patient sociodemographics listed in **Table S4** were identical across both clinical trials and were retained without modification. The only exception was the age of subjects and age at interview, both converted from months to years in RAISE-ETP. The body mass index of RAISE-ETP patients was converted from the imperial units system to the international units system. For diagnoses, we rearranged the items to cover schizophrenia diagnoses, schizophreniform diagnoses, schizoaffective diagnoses, and other diagnoses not covered in both clinical trials. For substance use scores, we selected item scores that asked the same domain. The only three overlapping domains were alcohol current use, cannabis current and cannabis previous use. The EUFEST trial covered a wider range of substances, while RAISE-ETP covered an “Other” substances variable, referring to any substance other than alcohol and cannabis. We performed the logistical OR operation in all the substances covered in EUFEST to obtain an “Other” substances variable.

For quality-of-life measures, we identified 7 items with high similarity from the Manchester Short Assessment of Quality of Life (MANSA) in EUFEST, and the Quality-of-Life survey (QoL) and 12-item health survey (SF-12) scales in RAISE-ETP. These scores covered the domains of job satisfaction, intimate relationships satisfaction, leisure and social activities, household satisfaction, sociosexual relationships, general health satisfaction and mental health satisfaction. MANSA, QoL and SF-12 have different ranges:

- MANSA scores range from 1 to 7: {1.0: 'Couldn't be worse', 2.0: 'Displeased', 3.0: 'Mostly dissatisfied', 4.0: 'Mixed', 5.0: 'Mostly satisfied', 6.0: 'Pleased', 7.0: 'Couldn't be better'}
- QoL scores range from 0 to 6: {0.0: 'almost complete failure', 2.0: 'significant failure', 4.0: 'below the person’s capacity', 6.0: 'fullfilment'}
- SF-12 scores range from 1 to 5: {1.0: 'excellent', 2.0: 'very good', 3.0: 'good', 4.0: 'fair', 5.0: 'poor'}

For each given score we calculated the min-max normalized the scores using the formula:

$$S_{standardized}=\frac{S-S_{min}}{S_{min}-S_{max}}$$

Considering that for the SF-12 scores, the minimum and the maximum values are inverted. For neurocognitive assessments, we identified 9 items that were identical or covered the same domains of neurocognition across the two different neurocognitive assessments:

- Verbal memory: the RAVLT is administered in both clinical trials, with 15 words and 5 trials. We included in the prediction battery the number of correct words for each trial and the total number of correct words.
- Information processing: the Wechsler Adult Intelligence Scale, Third Edition digital symbol coding test is administered in both trials, we included the symbol coding total score.
- Motor coordination: EUFEST applies the Purdue pegboard while RAISE applies a token motor task. We normalized the scores by calculating the Z scores following the equation below.
- Executive functioning: EUFEST uses the trail marking test, while RAISE uses the tower of London test. We normalize by using the z-score of the time difference between test B and test A (trail marking) and the total score of the tower of London test.

For these scores, we applied standardization across datasets using Z scores:

$$S_{z}=\frac{S- \mu}{\sigma}$$

Where S is the raw score, µ and σ is are the mean and standard deviation across all patients. Finally, antipsychotic medication was harmonized across both samples by generating a set of binary variables which determine whether an antipsychotic medication is prescribed at baseline visit. In EUFEST, antipsychotic medications were organized as main antipsychotic medication (one of the 5 in the study arms: haloperidol, olanzapine, quetiapine, amisulpride and ziprasidone), and then the registry of other antipsychotics given to patients. To harmonize them, we performed the logistic OR operation in the study arms and other antipsychotic variables that covered the same medication. Additionally, RAISE-ETP were organized by type of antipsychotic, and route of administration (oral and long-acting injectable). Since long acting injectables were not used in the EUFEST clinical trial, we performed the logistic OR operation in variables with the same medication but different routes of administration to obtain a combined variable of the specific medication.

**1.4 Machine learning analyses**

Machine learning pipelines were designed and run using the MATLAB multivariate pattern recognition software NeuroMiner version 1.2 (<https://github.com/neurominer-git>). The models are available upon request from the authors and can be accessed through NeuroMiner. Support vector machine (SVM) regressors and classifiers were trained to predict remission or relapse of depression symptoms in first episode psychosis patients at 6 months and 12 months follow-ups from data available at the first visit. Depression symptoms were measured using the Calgary Depression Scale for Schizophrenia. Regressor models were trained to predict the absolute total CDSS score change from baseline to follow-up (ΔCDSS_visit_). Classifiers were trained to predict two different labels. First, a positive or negative CDSS score change from baseline (±ΔCDSS_visit_), defined from the calculation of the absolute change in CDSS scores used in the regression models. Second, we designed a classification label that groups patients into those where a major depressive episode (MDE) is recorded at any time during the first 6 months after baseline, and those where it is not. MDEs were defined following Calgary guidelines (CDSS ≥ 7, 82% specificity and 85% sensitivity for predicting the presence of a major depressive episode)(5,6). The mathematical definition of these three labels is given below:

All SVM models trained in this study utilized the liblinear library (7). The classifier models were configured as L1-regularized L2-loss linear kernel support vector classifiers, incorporating class-size weighting of the model hyperplane. The slack parameter was optimized over the range [2^-4^, 2^4^] with an exponential step of 1. Regression models were configured as L2-regularized L1-loss linear kernel support vector regressors. The slack parameter was set to 1, while the epsilon parameter was optimized over the range [0.05, 0.3] with a step of 0.025. The tolerance (maximum gradient of the function used to compute the support vectors) was set to 0.01 in all cases. Optimal model selection was regularized using the CV_1_ test performance and aggregating top performing base learners (cross-parameter ensemble). Top models used for the ensemble were determined as models where performance was above a certain percentile. The optimal percentile was found by testing the best combination of models using the 75%, 80%, 85%, 90% and 95% percentiles for classification models, and 5%, 10%, 15%, 20%, and 25% percentiles for regression models. In all cases, preprocessing, training and validation steps were performed using a nested-pooled-cross-validation framework of 5 inner and outer permutations, and 5 inner and outer folds. At the outer cross-validation (CV_2_) level, we iteratively held back 1-fold as a validation sample, while the remaining data (4 outer folds) entered the inner cross-validation (CV_1_) cycle. In the inner CV (CV_1_) cycle, we iteratively held back 1 fold of the data as test sample, while the rest 4 folds are used as training set. The training set in the inner framework is used to train models, while the test set is used to identify which hyperparameter combinations perform better in cases not used directly for training. Finally, in the other fold, the fold that we left out as validation sample is used to validate the model selection in samples not seen by the hyperparameter optimization framework. The number of permutations indicates the number of times the samples are randomly shuffled, and the cross-validation process (inner or outer) is repeated. To assess the geographical generalizability of models, we repeated the model discovery process using a leave-site-out cross-validation (LSO-CV). Each of the independent sites (44 sites in EUFEST and 34 sites in RAISE-ETP) were iteratively held back in the outer CV level, while the remaining sites were used in the inner CV level. The same parameters (5 inner permutation and folds) were used in each inner CV cycle. This process was repeated 5 times at the outer CV level.

Within both cross-validation frameworks, variables were pre-processed before feeding them into the SVC or SVR algorithms. First, each variable was scaled independently from – 1 to 1. Variables with a variability ≤ 95% (meaning that 95% or more of patients have the same score) were removed as they were deemed non-informative. Following, missing values were imputed using a sequential k-nearest neighbor algorithm (n = 7 neighbors and using Euclidian as distance metric). Finally, features were scaled again from – 1 to 1. A greedy forward-search feature selection algorithm was used to select the best performing subset of features based on the CV1 training and testing data, with an early stopping of 10% and a stepping of 5% of the remaining variables in each iteration to avoid overfitting. To prevent information leakage between the training, test, and validation samples, NeuroMiner incorporates all these preprocessing steps within a nested cross-validation design. In this framework, CV_1_ training samples are used to compute preprocessing parameters (e.g., the maximum and minimum value for -1 to 1 scaling). These parameters are then used to preprocess the validation data. This crucial step ensures that the preprocessing parameters are derived exclusively from the training data, thereby preventing any information from the test or validation data from influencing the model during training.

Optimal models were selected using three performance metrics. For all regression models, the mean squared error (MSE) was used as optimization metric. For ±ΔCDSS classifiers, balanced accuracy (BAC) was used as metric. To address the high imbalance in sample sizes between the ±PPD groups, we utilized an enhanced balanced accuracy metric. The three metrics are defined mathematically as follows:

$$MSE=\frac{1}{n}\sum_{i=1}^{n} \left( \Delta CDSS_{observed}-\Delta CDSS_{predicted} \right)^{2}$$

$$BAC=\frac{1}{2} (\frac{TP}{TP+FN}+\frac{TN}{TN+FP})$$

$$EBAC=\frac{1}{100} (\frac{TP}{TP+FN}\cdot\frac{TN}{TN+FP})$$

Where:

- True positive (TP): the number of times the positive label (+ ΔCDSS or + PPD) is predicted correctly
- True negative (TN): the number of times the negative label (- ΔCDSS or - PPD) is predicted correctly
- False positive (FP): the number of times the positive label is predicted, but the negative label is observed
- False negative (FN): the number of times the negative label is predicted, but the positive label is observed

Additionally, we optimized the SVM classification threshold in the inner cycle of RN-CV or LSO-CV to account for residual class imbalances in our SVC model. For each inner fold, we implemented a wrapper-based, greedy forward-search feature selection algorithm to detect a combination of variables that improved the performance of the model. We designed the optimization criterion to be based on both training and test samples at the CV_1_ level. Due to the large number of variables, models trained with all the available data in each sample were trained using a wrapper designed to stop when 90% of the features were still in the pool (not selected), with a 5% step (5% of the total features are incorporated into the bag of variables at each cycle). Models trained with the harmonized variables (123 variables) stopped searching for features when 50% of the features were still in the pool, with a 10% step. The top selected features by the models were used to generate a feature mask (with either 10% or 50% of total features), and models were retrained across CV_1_ partitions with the optimized features to improve the generalizability of the models.

Permutation testing of the outcome labels was performed in all classifiers. The method was based on a previous publication(8), and involves the permutation of the outcome labels. We used 1000 randomized permutation of the labels. For each permutation, the models are retrained in the cross-validation framework using the respective feature/label subsets obtained from the observed-label analyses. For each permutation, the predictions from the random models are aggregated into a permuted ensemble prediction for each subject in the second cross-validation set (CV_2_). This process creates a null distribution of the performance metric for the prediction models when evaluated in the out-of-training (OOT) set. The significance of the observed test performance is determined by counting the number of times the permuted OOT BAC is greater than or equal to the observed performance metric, then dividing this count by the total number of permutations performed. The model's significance is assessed using a p threshold of 0.05.

Initially, models were trained independently with all the available data on the EUFEST or RAISE-ETP samples to predict ΔCDSS (regression) and ±ΔCDSS (classification) at 6- and 12-months follow-ups, as well as PPD (classification). These models were not externally validated due to the lack of a replication sample with identical variables. To overcome this limitation, we trained and validated respective new models using only the harmonized variables available in both samples. Specifically, we trained on either EUFEST or RAISE-ETP patients as discovery sample, reported the out-of-training (OOT) performance, and then validated the respective models in the external cohort using out-of-cross-validation (OOCV). This OOCV step involves applying the entire pipeline of operations developed during model discovery to the unseen sample and measuring the model's performance on this external cohort.

Stability and importance of predictive features were assessed using the probability of feature selection (proportion of inner cross-validation cycles where the absolute mean weight of features is higher than the standard error of the mean), and the overall cross-validation ratio (CVR, the sum of the median SVM weights across all inner folds divided by the standard deviation). The CVR measure, implemented in NeuroMiner 1.2, was inspired by the bootstrap ratio used in the Partial Least Squares literature (9). Similarly to the bootstrap ratio, the CVR of variable 𝑗 was defined as:

$$CVR_{j}=\frac{( \sum_{i=1}^{n=p_{\mathrm{CV}1}\cdot k_{\mathrm{CV}1} {\cdot r}_{\mathrm{CV}2}\cdot k_{\mathrm{CV}2}} \hat{w_{j}^{i}})/n}{\sigma_{\hat{w_{j}^{i}}}/\sqrt{n}}$$

Where 𝑛 is the size of the SVM ensemble, 𝑝_𝐶𝑉1_ is the number of CV_1_ permutations, 𝑘_𝐶𝑉1_ the number of CV_1_ folds, 𝑟_𝐶𝑉2_ the number of CV_2_ repetitions, 𝑘_𝐶𝑉2_ the number of CV2 folds, $\hat{w}_{j}^{i}$ the 𝑗^th^ element of the 𝑖^th^ normalized weight vector $\hat{w}^{i}=\frac{w^{i}}{\left\| w^{i} \right\|}$ in the SVM ensemble (7,10,11), and $\sigma_{\hat{w_{j}^{i}}}$ the standard deviation of $\hat{w}_{j}^{i}$. Akin to Z-scores, the CVR vectors or images were thresholded at CVR = ±3 to delineate stable pattern elements across the cross-validation experiment. The sign of the CVR score was also used as an indicator of the sign of the effect of the features in the predictions. On the other hand, the feature selection probability was calculated as follows:

1. For each inner cross-validation cycle (CV_1_), we compute the mean absolute weight of each feature (µ_j_) and the standard error of the mean (SEM_j_) for that feature.
2. We compare µ_j_ and SEM_j_:
   - If µ_j_ is greater than SEM_j_, the feature is considered selected for that CV_1_ cycle, and a binary value ($B_{j}$) value is set to 1.
   - Otherwise, the binary value is set to 0.
3. We sum the binary selection values across all CV_1_ cycles. Finally, we divide the sum by the number of partitions in the outer cross-validation cycle (CV_2_) to obtain the feature selection probability ($P_{j}$).

$$P_{j}=\frac{\sum_{i=1}^{n=p_{\mathrm{CV}2}\cdot k_{\mathrm{CV}2}} B_{j}^{i}}{p_{\mathrm{CV}2}\cdot k_{\mathrm{CV}2}}$$

A selection probability higher than 0.5 indicates that the feature is consistently given a significant importance by models in more than 50% of the inner cycles. This indicates that the feature is relevant to generate predictions across the folds. Finally, models were retrained and externally validated following the same procedure but using a condensed version of the most predictive variables, where only those with a selection probability higher than 50% were retained. In this case, the feature selection wrapper was disabled, since just a subsection of the previously selected features was being used. This approach assessed whether a streamlined tool with fewer, more relevant variables could maintain the model's performance.

**1.5 Post hoc analyses**

To assess the consistency and robustness of model predictions, we calculated the correlation of SVM decision scores and the labelling agreement between OOT and OOCV predictions of the same patients. For all classification labels, we plotted the OOT vs. OOCV SVM decision scores (**Figure S10**), calculated the Pearson’s correlation coefficient (r), the coefficient of determination (R^2^) and the class agreement of the predictions (percentage of agreement between OOT and OOCV labels). To evaluate the model's ability to differentiate between negative symptoms and depressive symptoms, we conducted an additional analysis. Specifically, we used models trained with depressive symptom labels to predict negative symptom labels. We then assessed the significance of these predictions using permutation testing with 1000 label permutations. To do this, we used analogous label definitions for the symptom trajectories using the negative subscale (N1 to N7) score of the Positive and Negative Syndrome Scale (PANSS) as follows:

Additionally, for the PPD model, we classified patients in those who had a score of 3 or higher on any symptom in the PANSS negative subscale assessment during the 6 months following the baseline visit (non-remission group) and those who did not (remission group):

Where represents any individual PANSS negative subscale symptom score. We repeated this analysis using instead the Marder negative symptom factor scores in the PANSS scale, including N1: blunted affect, N2: emotional withdrawal, N3: poor rapport, N4: social withdrawal, N6: lack of spontaneity in conversation, G7: motor retardation, G13: disturbance of volition and G16: active social avoidance (12,13), but otherwise following the same protocol.

In addition, we evaluated whether the PPD model predictive pattern could also predict positive symptom non-remission. We used a 50% decrease in the PANSS positive total at 6 months follow-up to define positive non-remission as follows:

Where $PANSS\_POS_{visit}$ and represent the PANSS positive total score at the follow up visit and baseline, respectively. A value of seven is subtracted from the denominator to account for the fact that PANSS absence of a symptom is represented as a 1, and the positive subscale has 7 symptoms. All results are shown in **Table S11**.

Model calibration at the OOT and OOCV level was assessed using calibration curve analysis and measuring the expected calibration error (ECE; see **Figure S11**). The ECE was calculated as the weighted average difference between the fraction of correctly predicted outcomes and predicted probabilities across binned probability ranges (14). Five bins were selected to calculate the reliability diagrams and the ECE.

The models’ prediction performances at the OOT and OOCV level and trained using either the EUFEST or RAISE-ETP sample were visually compared using receiver operating characteristic (ROC) curves and area under the curve (AUC-ROC). The relevance of features was assessed using CVR scores and feature selection probabilities (see above). To identify robust features consistently selected across independent model trainings, feature selection probabilities were calculated separately for models trained on the EUFEST and RAISE-ETP samples. These probabilities were then plotted on Cartesian axes, allowing for a visual comparison of the features consistently chosen in both datasets, only in one of the datasets, or in none (**Figure S2** and **Figure S9**).

To evaluate the performance difference of models in the RAISE-ETP patients relative to EUFEST patients, we examined potential differences between both samples using inferential statistics. First, we univariately compared baseline characteristics of EUFEST and RAISE-ETP patients (**Table S7**), including sociodemographics, psychopathology (including depression symptoms), substance use, diagnoses, and antidepressant prescriptions at baseline. We used mixed-design ANOVAs and mixed-effects linear regression analyses to compare depression treatment trajectories of RAISE-ETP patients with and without antidepressant prescription, in the NAVIGATE or community-based treatment and accounting for other mixed effects (long-acting injectables, LAI) and random effects (treatment sites). Results of mixed-design ANOVAs are shown in **Table S12** for antidepressant treatment and **Table S13** for treatment group. Results of mixed-effects linear regressions are shown in **Table S15.** We repeated this analysis only considering patients with a major depressive disorder at baseline (**Table S16**). To compare the efficacy of types of antidepressants prescribed, we performed an analogous mixed design ANOVA analysis and grouped patients based on type of antidepressant. We grouped patients prescribed with the following types of antidepressants (between-subjects factor):

- Selective serotonin reuptake inhibitors (SSRIs), including citalopram, escitalopram oxalate, fluoxetine, fluvoxamine, paroxetine and sertraline.
- Serotonin and norepinephrine reuptake inhibitors (SNRIs), including desvenlafaxine, duloxetine and venlafaxine.
- Tricyclic antidepressants (TCAs), including amitriptyline and doxepin.
- Norepinephrine–dopamine reuptake inhibitor (NDRIs), including bupropion.
- Serotonin antagonist and reuptake inhibitors (SARIs), including trazodone.

And compared the trajectory of depression symptoms across the three time points specified above (within subjects’ factor; **Table S14** and **Figure S13**).

Finally, to assess the impact of treatment arm and antidepressants on model predictions, we used Kolmogorov–Smirnov (KS) tests to compare SVM decision score distributions between these two factors. To analyze model misclassifications, we ranked patients by SVM decision scores, computed rolling misclassification rates to derive cumulative error functions. We then tested differences in error distributions across SVM decision scores between various patient groups using the KS test (**Figure S14** and **Figure S15**). The procedure involved the following steps:

1. For each model, we ranked RAISE-ETP patients based on their SVM decision scores.
2. We computed the rolling misclassification progressively from the lowest to the highest SVM dcision score.
3. We normalized the cumulative misclassification rates to a scale from 0 to 1.
4. We plotted the normalized cumulative misclassification rates against the SVM decision scores to derive the cumulative error function.

We finally tested the differences in the distribution of errors across SVM decision scores between patients with prescribed antidepressants and patients without, and patients in the NAVIGATE program and patients in community-based treatment using the KS test.

**2. Supplementary Results**

**2.1 Machine learning analyses with all available variables**

Initially, we utilized all the available variables in the EUFEST and RAISE-ETP samples independently to train the linear SVMs. The performance metrics are shown in **Table S8**. In EUFEST, classifiers predicted ± ΔCDSS at 6 months follow-up with BAC of 76.93% (sensitivity, 76.19%; specificity, 77.67%), at 12 months follow-up with a BAC of 82.52% (sensitivity, 84.78%; specificity, 80.26%), and ± PPD with BAC of 66.49% (sensitivity, 62.03%; specificity, 70.95%). Predictor variables differed across model type (regressors vs. classifiers) and prediction labels but covered equivalent domains. Significant predictors as determined by the probability of feature selection and the cross-validated ratio (CVR) were depressive symptoms at baseline, general psychopathology, neurocognitive performance (digit symbol substation test, trail making test, and verbal memory) and blood prolactin levels (**Figures S1** and **S2**). In RAISE-ETP, classifiers predicted ±ΔCDSS at 6 months follow-up with BAC of 68.83% (sensitivity, 66.10%; specificity, 71.55%), at 12 months follow-up with a BAC of 66.76% (sensitivity, 68.31%; specificity, 65.21%), and ± PPD with BAC of 66.98% (sensitivity, 57.78%; specificity, 76.19%). Significant predictors at baseline in this sample were depressive symptoms, general psychopathology scores, neurocognitive performance (digital sequencing), side effects (nausea), blood levels of alanine transferase (ALT), and in the ±PPD case, the treatment group (community care or NAVIGATE), as well as sex and fat percentage (**Figures S3** and **S4**).

**2.2 Consistency of model predictions**

We assessed the consistency of model predictions by calculating the correlation of the SVM decision scores and the labelling agreement between OOT and OOCV predictions of the same patients (**Figure S10**). SVM decision scores of EUFEST patients were highly correlated, yielding a Pearson’s r of 0.87 (P<.001, agreement=84.06%), 0.74 (P<.001, agreement=80.62%) and 0.57 (P<.001, agreement=73.75%) for the ±ΔCDSS at 6 months, 12 months, and the ±PPD label, respectively. Decision scores of RAISE-ETP patients followed the same trend, yielding a Pearson’s r of 0.87 (P<.001, agreement=76.07%), 0.72 (P<.001, agreement=81.20% agreement) and 0.68 (P<.001, agreement=75.64%) for the ±ΔCDSS at 6 months, 12 months, and the ±PPD label, respectively. In addition, we evaluated the models’ specificity, i.e. their ability to differentiate between negative and depressive symptoms and found that all classifiers performed poorly in predicting PANSS negative symptom labels (**Table S11**). Furthermore, we retrained models without total symptom scores (CDSS total and PANSS positive, negative, general and total scores), showing unchanged predictive performance (**Table S10**). Finally, we retrained the models using only biological variables and only non-biological variables, and then compared the model predictions obtained from training with all available harmonized variables against those obtained from training with non-biological variables alone. We found that biological variables do not significantly predict the ±ΔCDSS outcome, and marginally predict the ±PPD outcome (**Table S20**). However, when removing biological variables, ±ΔCDSS models are significantly more biased towards the predominant group, evidenced by larger differences between sensitivity and specificity and lower BAC, while ±PPD model predictions remain unchanged (**Table S21**).

**2.3 Effect of treatment and antidepressant use on depression in RAISE-ETP**

We studied the influence of treatment assignment and antidepressant prescription at baseline on depression courses in RAISE-ETP. Overall, patients had a higher rate of antidepressant prescriptions in RAISE compared to EUFEST (**Table S7**). Patients in the NAVIGATE treatment program were prescribed less antidepressants than patients in community care (41.18% vs. 27.27%, P = 0.035). For RAISE-ETP patients, we compared CDSS scores across different treatment programs and antidepressant use (**Figure 3A**). Fitting a mixed linear model (**Table S15**), we found significant main effects of treatment program (β = -1.47, P = 0.027) and antidepressant prescriptions (β = -1.58, P = 0.001) on CDSS scores across visits. A trending interaction effect between antidepressant prescriptions and treatment also indicated that the NAVIGATE treatment had a smaller effect on the CDSS scores of patients who were not prescribed antidepressants (β = 0.95, P = 0.130). We repeated this analysis only for patients with an indication of major depressive episode (**Table S16** and **Figure S13**) as defined as baseline CDSS≥7, and observed the same effects. Furthermore, symptomatic remission probabilities did not significantly differ between antidepressants prescribed (**Figure S13**).

**2.4 Effect of history of depressive episodes in ±PPD model predictions**

In the EUFEST sample, where history of major depressive episodes was available, we conducted a secondary analysis to examine whether the model’s decision scores for predicting future depressive episodes (±PPD) varied according to patients’ history of major depressive disorder (MDD). The results revealed a large effect size (Cohen's *d* = 0.75, *t* = 3.41, *p* < .001), with individuals with a history of MDD more likely to receive negative decision scores, indicating a prediction of future depressive symptoms (+PPD). This finding is consistent with prior research demonstrating that a history of depression is one of the strongest predictors of future depressive episodes, both in MDD (15) and in overlapping conditions such as psychotic depression (16).

**Supplementary References**

1. Fleischhacker WW, Keet IPM, Kahn RS. The European First Episode Schizophrenia Trial (EUFEST): Rationale and design of the trial. Schizophr Res [Internet]. 2005;78(2):147–56. Available from: https://www.sciencedirect.com/science/article/pii/S0920996405002379

2. Kane JM, Schooler NR, Marcy P, Correll CU, Brunette MF, Mueser KT, et al. The RAISE early treatment program for first-episode psychosis: background, rationale, and study design. J Clin Psychiatry. 2015;76(3):16590.

3. Mueser KT, Penn DL, Addington J, Brunette MF, Gingerich S, Glynn SM, et al. The NAVIGATE program for first-episode psychosis: rationale, overview, and description of psychosocial components. Psychiatric Services. 2015;66(7):680–90.

4. McElroy E, Moltrecht B, Scopel Hoffmann M, Wood T, Ploubidis G. Harmony - A global platform for contextual harmonisation, translation and cooperation in mental health research. 2022.

5. Addington D, Addington J, Maticka-Tyndale E, Joyce J. Reliability and validity of a depression rating scale for schizophrenics. Schizophr Res [Internet]. 1992;6(3):201–8. Available from: https://www.sciencedirect.com/science/article/pii/092099649290003N

6. Addington D, Addington J, Maticka-tyndale E. Assessing Depression in Schizophrenia: The Calgary Depression Scale. British Journal of Psychiatry [Internet]. 2018/08/06. 1993;163(S22):39–44. Available from: https://www.cambridge.org/core/product/B2C4A2B33FD63E4B690F455572642A63

7. Fan RE, Chang KW, Hsieh CJ, Wang XR, Lin CJ. LIBLINEAR: A library for large linear classification. the Journal of machine Learning research. 2008;9:1871–4.

8. Golland P, Fischl B. Permutation Tests for Classification: Towards Statistical Significance in Image-Based Studies. In: Taylor C, Noble JA, editors. Information Processing in Medical Imaging. Berlin, Heidelberg: Springer Berlin Heidelberg; 2003. p. 330–41.

9. Krishnan A, Williams LJ, McIntosh AR, Abdi H. Partial Least Squares (PLS) methods for neuroimaging: A tutorial and review. Neuroimage [Internet]. 2011;56(2):455–75. Available from: https://www.sciencedirect.com/science/article/pii/S1053811910010074

10. Chang CC, Lin CJ. LIBSVM: A library for support vector machines. ACM Trans Intell Syst Technol [Internet]. 2011 May;2(3). Available from: https://doi.org/10.1145/1961189.1961199

11. Polikar R. Ensemble based systems in decision making. IEEE Circuits and Systems Magazine. 2006;6(3):21–45.

12. Marder SR, Davis JM, Chouinard G. The effects of risperidone on the five dimensions of schizophrenia derived by factor analysis: combined results of the North American trials. J Clin Psychiatry. 1997;58(12):538–46.

13. Marder SR, Umbricht D. Negative symptoms in schizophrenia: Newly emerging measurements, pathways, and treatments. Schizophr Res [Internet]. 2023;258:71–7. Available from: https://www.sciencedirect.com/science/article/pii/S0920996423002359

14. Guo C, Pleiss G, Sun Y, Weinberger KQ. On calibration of modern neural networks. In: International conference on machine learning. PMLR; 2017. p. 1321–30.

15. Teutenberg L, Stein F, Thomas-Odenthal F, Usemann P, Brosch K, Winter N, et al. Machine learning-based prediction of illness course in major depression: The relevance of risk factors. J Affect Disord [Internet]. 2025;374:513–22. Available from: https://www.sciencedirect.com/science/article/pii/S0165032725000771

16. Carter E, Banerjee S, Alexopoulos GS, Bingham KS, Marino P, Meyers BS, et al. Prediction of remission of pharmacologically treated psychotic depression: A machine learning approach. J Affect Disord [Internet]. 2025;381:291–7. Available from: https://www.sciencedirect.com/science/article/pii/S0165032725005658

Table S1. Data domains and collection times in the EUFEST sample.

| Week | 0 | 2 | 4 (1 m) | 6 | 8 (2 m) | 12 (3 m) | 26 (6 m) | 39 (9 m) | 52 (12 m) |
| --- | --- | --- | --- | --- | --- | --- | --- | --- | --- |
| Demographic data | X |  |  |  |  |  |  |  |  |
| MINI-plus | X |  |  |  |  |  |  |  |  |
| Concomitant medication | X |  | X |  | X | X | X | X | X |
| Clinical global impression (CGI) | X | X | X | X | X | X | X | X | X |
| General assessment of functioning (GAF) scale | X |  | X |  | X | X | X | X | X |
| Positive and negative syndrome scale (PANSS) | X |  | X |  |  | X | X | X | X |
| Calgary depression scale for schizophrenia (CDSS) | X |  | X |  |  | X | X |  | X |
| Manchester short assessment of quality of life scale (MANSA) | X |  |  |  |  | X |  |  | X |
| St. Hans rating scale for extrapyramidal syndromes | X |  | X |  |  | X | X | X | X |
| Camberwell assessment of needs (CAN) | X |  |  |  |  |  | X |  | X |
| UKU side effect rating scale | X |  | X |  |  | X | X | X | X |
| Hayward scale (compliance) |  |  | X |  |  |  | X |  | X |
| Scale for the deficit syndrome (SDS) |  |  |  |  |  | X |  |  | X |
| Structured clinical interview for the symptom onset in schizophrenia (SOS) | X | | | | | | | | | |
| Drug attitude inventory (DAI) |  |  |  |  |  | X |  |  | X |
| Edinburgh handedness inventory (EHI) | X | | | | | | | | | |
| Electrocardiogram (ECG) | X |  |  |  |  |  |  |  | X |
| Substance abuse | X |  |  |  |  |  | X |  | X |
| Neurocognitive battery (RAVLT, TMT, Purdue pegboard and digit-symbol coding) | X |  |  |  |  |  | X |  |  |
| Weight/ waist | X |  |  |  |  | X | X | X | X |
| BP/pulse | X |  |  |  |  |  |  |  |  |
| Lab tests including prolactin | X |  |  |  |  |  | X |  | X |

Abbreviations: RAVLT, Rey auditory verbal learning test; TMT, trail making test.

Table S2. Data domains and collection times in the RAISE-ETP sample.

| Month | 0 | 3 | 6 | 12 | 18 | 24 |
| --- | --- | --- | --- | --- | --- | --- |
| Demographic data | X |  |  |  |  |  |
| Patient education/occupation/children  (PTEdOcCh) | X |  |  | X |  | X |
| Medical history (MEDX) | X |  |  |  |  |  |
| Positive and negative syndrome scale (PANSS) | X |  | X | X | X | X |
| Calgary depression scale for schizophrenia (CDSS) | X |  | X | X | X | X |
| Clinical global Impression (CGI) | X |  | X | X | X | X |
| Medications at consent (and monthly afterwards) | X |  |  |  |  |  |
| Oral antipsychotic medication adherence review (ADH) | X | X | X | X | X | X |
| Services utilization recording form (monthly and quarterly) |  |  |  |  |  |  |
| 12-Item health survey (SF-12) (monthly) | X | X | X | X | X | X |
| Vitals | X | X | X | X | X | X |
| Lab test including metabolic biomarkers | X | X | X | X | X | X |
| Substance use (monthly) | X | X | X | X | X | X |
| Movement side effects (EPSAIMS) | X | X | X | X |  | X |
| Side effects scale (SelfSE) | X | X | X | X | X | X |
| Well-being scale (WellBe) | X | X | X | X | X | X |
| Mental health recovery measure (MHRM) | X | X | X | X | X | X |
| Autonomy support scale (AutSup) | X | X | X | X | X | X |
| Brief evaluation of medication influences and beliefs scale (BEMIB) | X | X | X | X | X | X |
| Stigma scale | X | X | X | X | X | X |
| Patient self-rated globals | X | X | X | X | X | X |
| Neurocognitive testing (BACS) | X |  |  | X |  | X |
| Family assessment scale (FAMBURD) | X | X | X | X | X | X |
| Recovery outcomes assessment (FAMRECOV) | X | X | X | X | X | X |
| Intent to Attend | X | X | X | X | X | X |
| Quality of life scale (QOL) | X |  | X | X | X | X |

Abbreviations: BACS, brief assessment of cognition in schizophrenia; EPSAIMS, extrapyramidal symptoms and abnormal involuntary movement scale.

Table S3. Harmonization of variables across both datasets.

| Variable | Description | EUFEST item(s) | RAISE-ETP item(s) | Changes |
| --- | --- | --- | --- | --- |
| Quality of life | | | | |
| job_sat | Job satisfaction | V1MA02 | QOL13 | Standardized scores from 0 to 1 |
| rel_sat | Intimate relationship satisfaction | V1MA06 | QOL06 | Standardized scores from 0 to 1 |
| leis_sat | Leisure and social satisfaction | V1MA07 | QOL05 | Standardized scores from 0 to 1 |
| house_sat | Household satisfaction | V1MA12 | QOL02 | Standardized scores from 0 to 1 |
| sociosexual_sat | Sociosexual satisfaction | V1MA13 | QOL09 | Standardized scores from 0 to 1 |
| gen_health_sat | General health satisfaction | V1MA15 | SF01 | Standardized scores from 0 to 1 |
| ment_health_sat | Mental health satisfaction | V1MA16 | SRF019 | Standardized scores from 0 to 1 |
| Substance use | | | | |
| SA_Alcohol | Alcohol current use | V1SAL06 | subus25 | No |
| SA_Cannabis | Cannabis current use | V1SAL03 | ca824 | No |
| SA_Cannabis_1 | Cannabis previous use | V1SA06 | subus29 | No |
| SA_Other | Other substance current use | V1SAL08: cocaine use  V1SAL11: amphetamine use  V1SAL14: heroin use  V1SAL17: ecstasy use  V1SAL20: LSD use  V1SAL23: other substances use | subus30 | Logical OR operation in EUFEST variables |
| Neurocognitive assessments | | | | |
| vm_tr1 | Verbal memory correct answers trial 1 | V1NEU47 | bacvm1 | No |
| vm_tr2 | Verbal memory correct answers trial 2 | V1NEU50 | bacvm2 | No |
| vm_tr3 | Verbal memory correct answers trial 3 | V1NEU53 | bacvm3 | No |
| vm_tr4 | Verbal memory correct answers trial 4 | V1NEU56 | bacvm4 | No |
| vm_tr5 | Verbal memory correct answers trial 5 | V1NEU59 | bacvm5 | No |
| vm_total | Verbal memory total number of correct answers | V1NESUMCORR | bacs_vmttot | No |
| sc_total | Symbol coding score | V1NEU07 | bacs_sc_total | No |
| hand_total | Hand coordination score | V1NEU10: # pegs with dominant hand  V1NEU11: # pegs with non-dominant hand  V1NEU12: # pegs with both hands | bactmts | For EUFEST, we add the scores from dominant, non-dominant and both hands to obtain a total coordination score. Standardized scores calculating the Z score |
| exec_total | Executive functioning | V1NEU04MINUS01: trailmarking B – A time | bacs_tl_total: Tower of London total | Standardized scores calculating the Z score |
| Diagnose | | | | |
| diag_1 | Schizophrenia | code1: schizophrenia paranoid type  code2: schizophrenia catatonic type  code3: schizophrenia disorganized type  code 4: schizophrenia unspecified type | dxbase_1.0: schizophrenia | Logical OR operation in EUFEST variables |
| diag_2 | Schizophreniform disorder | code5: schizophreniform disorder | dxbase_4.0: schizophreniform provisional  dxbase_5.0: schizophreniform definite | Logical OR operation in RAISE-ETP variables |
| diag_3 | Schizoaffective disorder | code6: schizoaffective disorder | dxbase_2.0: schizoaffective bipolar dxbase_3.0: schizoaffective depressive | Logical OR operation in RAISE-ETP variables |
| diag_4 | Other psychosis diagnoses | code7: schizophrenia residual type | dxbase_6.0: brief psychotic episode dxbase_7.0: psychotic disorder not otherwise specified | Logical OR operation in RAISE-ETP variables |
| Medications | | | | |
| Risperidone | Risperidone treatment | OTHERANTIPSY_Risperidone | adhetpc1_4.0  adhetpc1_22.0 | No |
| Clozapine | Clozapine treatment | OTHERANTIPSY_Clozapine | adhetpc1_21.0 |  |
| Olanzapine | Olanzapine treatment | OTHERANTIPSY_Olanzapine  StudyArm_2 | adhetpc1_2.0 | Logical OR operation in EUFEST variables |
| Quetiapine | Quetiapine treatment | OTHERANTIPSY_Quetiapine  StudyArm_3 | adhetpc1_3.0 | Logical OR operation in EUFEST variables |
| Sulpiride | Sulpiride treatment | OTHERANTIPSY_Sulpiride | N/A | No |
| Haloperidol | Haloperidol treatment | OTHERANTIPSY_Haloperidol  StudyArm_1 | adhetpc1_13.0  adhetpc1_25.0 | Logical OR operation in EUFEST variables |
| Zuclopenthixol | Zuclopenthixol treatment | OTHERANTIPSY_Zuclopenthixol | N/A | No |
| Perphenazine | Perphenazine treatment | OTHERANTIPSY_Perphenazine | adhetpc1_14.0 | No |
| Chlorpromazine | Chlorpromazine treatment | OTHERANTIPSY_Chlorpromazine | adhetpc1_11.0 | No |
| Levomepromazine | Levomepromazine treatment | OTHERANTIPSY_Levomepromazine | N/A | No |
| Amisulpride | Amisulpride treatment | OTHERANTIPSY_Amisulpride  StudyArm_4 | N/A | Logical OR operation in EUFEST variables |
| Prothipendyl | Prothipendyl treatment | OTHERANTIPSY_Prothipendyl | N/A | No |
| Clotiapine | Clotiapine treatment | OTHERANTIPSY_Clotiapine | N/A | No |
| Gabapentin | Gabapentin treatment | OTHERANTIPSY_Gabapentin | N/A | No |
| Perazine | Perazine treatment | OTHERANTIPSY_Perazine | N/A | No |
| Flupentixol | Flupentixol treatment | OTHERANTIPSY_Flupentixol | N/A | No |
| Penfluridol | Penfluridol treatment | OTHERANTIPSY_Penfluridol | N/A | No |
| Pimozide | Pimozide treatment | OTHERANTIPSY_Pimozide | N/A | No |
| Promazine | Promazine treatment | OTHERANTIPSY_Promazine | N/A | No |
| Fluphenazine | Fluphenazine treatment | OTHERANTIPSY_Fluphenazine | adhetpc1_12.0  adhetpc1_24.0 | Logical OR operation in RAISE-ETP variables |
| Loxapine | Loxapine treatment | OTHERANTIPSY_Loxapine | adhetpc1_19.0 | Logical OR operation in EUFEST variables |
| Ziprasidone | Ziprasidone treatment | OTHERANTIPSY_Ziprasidone  StudyArm_5 | adhetpc1_5.0 | Logical OR operation in EUFEST variables |
| Chlorprothixene | Chlorprothixene treatment | OTHERANTIPSY_Chlorprothixene | N/A | No |
| Trifluoperazine | Trifluoperazine treatment | OTHERANTIPSY_Trifluoperazine | N/A | No |
| Cyamemazine | Cyamemazine treatment | OTHERANTIPSY_Cyamemazine | N/A | No |
| Pipamperone | Pipamperone treatment | OTHERANTIPSY_Pipamperone | N/A | No |
| Zotepine | Zotepine treatment | OTHERANTIPSY_Zotepine | N/A | No |
| Thioridazine | Thioridazine treatment | OTHERANTIPSY_Thioridazine | N/A | No |
| Prochlorperazine | Prochlorperazine treatment | OTHERANTIPSY_Prochlorperazine | N/A | No |
| Antidepressant | Antidepressant treatment | CONCOMMED_ANTIDEPRESSANTS | adhetpc1_30.0  adhetpc1_31.0  adhetpc1_32.0  adhetpc1_34.0  adhetpc1_35.0  adhetpc1_36.0  adhetpc1_37.0  adhetpc1_38.0  adhetpc1_39.0  adhetpc1_41.0  adhetpc1_42.0  adhetpc1_43.0  adhetpc1_44.0 | Logical OR operation in RAISE-ETP variables |

Variables included here are those where numerical transformation was applied to harmonize it across the EUFEST and RAISE-ETP datasets.

Table S4. Battery of harmonized variables and value ranges.

| Variable | Description | Instrument | Values |
| --- | --- | --- | --- |
| SA_Alcohol | Alcohol current use | Substance abuse questionnaire | {0.0: 'no', 1.0: 'yes'} |
| SA_Cannabis | Cannabis current use | Substance abuse questionnaire | {0.0: 'no', 1.0: 'yes'} |
| SA_Cannabis_1 | Cannabis previous use | Substance abuse questionnaire | {0.0: 'no', 1.0: 'yes'} |
| SA_Other | Other substances current use | Substance abuse questionnaire | {0.0: 'no', 1.0: 'yes'} |
| V1CD01 | CDSS score 01: 'Depressed mood' | Calgary Depression Scale for Schizophrenia | {0.0: 'absent', 1.0: 'mild', 2.0: 'moderate', 3.0: 'severe'} |
| V1CD02 | CDSS score 02: 'Hopelessness' | Calgary Depression Scale for Schizophrenia | {0.0: 'absent', 1.0: 'mild', 2.0: 'moderate', 3.0: 'severe'} |
| V1CD03 | CDSS score 03: 'Self-deprecation' | Calgary Depression Scale for Schizophrenia | {0.0: 'absent', 1.0: 'mild', 2.0: 'moderate', 3.0: 'severe'} |
| V1CD04 | CDSS score 04: 'Guilty ideas of reference' | Calgary Depression Scale for Schizophrenia | {0.0: 'absent', 1.0: 'mild', 2.0: 'moderate', 3.0: 'severe'} |
| V1CD05 | CDSS score 05: 'Pathological guilt' | Calgary Depression Scale for Schizophrenia | {0.0: 'absent', 1.0: 'mild', 2.0: 'moderate', 3.0: 'severe'} |
| V1CD06 | CDSS score 06: 'Morning depression' | Calgary Depression Scale for Schizophrenia | {0.0: 'absent', 1.0: 'mild', 2.0: 'moderate', 3.0: 'severe'} |
| V1CD07 | CDSS score 07: 'Early wakening' | Calgary Depression Scale for Schizophrenia | {0.0: 'absent', 1.0: 'mild', 2.0: 'moderate', 3.0: 'severe'} |
| V1CD08 | CDSS score 08: 'Suicide' | Calgary Depression Scale for Schizophrenia | {0.0: 'absent', 1.0: 'mild', 2.0: 'moderate', 3.0: 'severe'} |
| V1CD09 | CDSS score 09: 'Observed depression' | Calgary Depression Scale for Schizophrenia | {0.0: 'absent', 1.0: 'mild', 2.0: 'moderate', 3.0: 'severe'} |
| V1CDTOTAL | CDSS total score | Calgary Depression Scale for Schizophrenia | Total score of the above 0-27 |
| V1pa01 | PANSS P1: 'Delusions' | Positive and Negative Syndrome Scale | {1.0: 'absent', 2.0: 'minimal', 3.0: 'mild', 4.0: 'moderate', 5.0: 'moderate/severe', 6.0: 'severe', 7.0: 'extreem'} |
| V1pa02 | PANSS P2: 'Conceptual disorganization' | Positive and Negative Syndrome Scale | {1.0: 'absent', 2.0: 'minimal', 3.0: 'mild', 4.0: 'moderate', 5.0: 'moderate/severe', 6.0: 'severe', 7.0: 'extreem'} |
| V1pa03 | PANSS P3: Hallucinations' | Positive and Negative Syndrome Scale | {1.0: 'absent', 2.0: 'minimal', 3.0: 'mild', 4.0: 'moderate', 5.0: 'moderate/severe', 6.0: 'severe', 7.0: 'extreem'} |
| V1pa04 | PANSS P3: 'Excitement' | Positive and Negative Syndrome Scale | {1.0: 'absent', 2.0: 'minimal', 3.0: 'mild', 4.0: 'moderate', 5.0: 'moderate/severe', 6.0: 'severe', 7.0: 'extreem'} |
| V1pa05 | PANSS P4: 'Grandiosity' | Positive and Negative Syndrome Scale | {1.0: 'absent', 2.0: 'minimal', 3.0: 'mild', 4.0: 'moderate', 5.0: 'moderate/severe', 6.0: 'severe', 7.0: 'extreem'} |
| V1pa06 | PANSS P5: 'Suspiciousness/persecution' | Positive and Negative Syndrome Scale | {1.0: 'absent', 2.0: 'minimal', 3.0: 'mild', 4.0: 'moderate', 5.0: 'moderate/severe', 6.0: 'severe', 7.0: 'extreem'} |
| V1pa07 | PANSS P6: 'Hostility' | Positive and Negative Syndrome Scale | {1.0: 'absent', 2.0: 'minimal', 3.0: 'mild', 4.0: 'moderate', 5.0: 'moderate/severe', 6.0: 'severe', 7.0: 'extreem'} |
| V1pa08 | PANSS N1: 'Blunted effect' | Positive and Negative Syndrome Scale | {1.0: 'absent', 2.0: 'minimal', 3.0: 'mild', 4.0: 'moderate', 5.0: 'moderate/severe', 6.0: 'severe', 7.0: 'extreem'} |
| V1pa09 | PANSS N2: 'Emotional withdrawal' | Positive and Negative Syndrome Scale | {1.0: 'absent', 2.0: 'minimal', 3.0: 'mild', 4.0: 'moderate', 5.0: 'moderate/severe', 6.0: 'severe', 7.0: 'extreem'} |
| V1pa10 | PANSS N3: 'Poor rapport' | Positive and Negative Syndrome Scale | {1.0: 'absent', 2.0: 'minimal', 3.0: 'mild', 4.0: 'moderate', 5.0: 'moderate/severe', 6.0: 'severe', 7.0: 'extreem'} |
| V1pa11 | PANSS N4: 'Passive/apathetic social withdrawal' | Positive and Negative Syndrome Scale | {1.0: 'absent', 2.0: 'minimal', 3.0: 'mild', 4.0: 'moderate', 5.0: 'moderate/severe', 6.0: 'severe', 7.0: 'extreem'} |
| V1pa12 | PANSS N5: 'Difficulty in abstract thinking' | Positive and Negative Syndrome Scale | {1.0: 'absent', 2.0: 'minimal', 3.0: 'mild', 4.0: 'moderate', 5.0: 'moderate/severe', 6.0: 'severe', 7.0: 'extreem'} |
| V1pa13 | PANSS N6: 'Lack of spontaneity' | Positive and Negative Syndrome Scale | {1.0: 'absent', 2.0: 'minimal', 3.0: 'mild', 4.0: 'moderate', 5.0: 'moderate/severe', 6.0: 'severe', 7.0: 'extreem'} |
| V1pa14 | PANSS N7: 'Stereotyped thinking' | Positive and Negative Syndrome Scale | {1.0: 'absent', 2.0: 'minimal', 3.0: 'mild', 4.0: 'moderate', 5.0: 'moderate/severe', 6.0: 'severe', 7.0: 'extreem'} |
| V1pa15 | PANSS G1: 'Somatic concern' | Positive and Negative Syndrome Scale | {1.0: 'absent', 2.0: 'minimal', 3.0: 'mild', 4.0: 'moderate', 5.0: 'moderate/severe', 6.0: 'severe', 7.0: 'extreem'} |
| V1pa16 | PANSS G2: 'Anxiety' | Positive and Negative Syndrome Scale | {1.0: 'absent', 2.0: 'minimal', 3.0: 'mild', 4.0: 'moderate', 5.0: 'moderate/severe', 6.0: 'severe', 7.0: 'extreem'} |
| V1pa17 | PANSS G3: 'Guilt feelings' | Positive and Negative Syndrome Scale | {1.0: 'absent', 2.0: 'minimal', 3.0: 'mild', 4.0: 'moderate', 5.0: 'moderate/severe', 6.0: 'severe', 7.0: 'extreem'} |
| V1pa18 | PANSS G4: 'Tension' | Positive and Negative Syndrome Scale | {1.0: 'absent', 2.0: 'minimal', 3.0: 'mild', 4.0: 'moderate', 5.0: 'moderate/severe', 6.0: 'severe', 7.0: 'extreem'} |
| V1pa19 | PANSS G5: 'Mannerisms and posturing' | Positive and Negative Syndrome Scale | {1.0: 'absent', 2.0: 'minimal', 3.0: 'mild', 4.0: 'moderate', 5.0: 'moderate/severe', 6.0: 'severe', 7.0: 'extreem'} |
| V1pa20 | PANSS G6: 'Depression' | Positive and Negative Syndrome Scale | {1.0: 'absent', 2.0: 'minimal', 3.0: 'mild', 4.0: 'moderate', 5.0: 'moderate/severe', 6.0: 'severe', 7.0: 'extreem'} |
| V1pa21 | PANSS G7: 'Motor retardation' | Positive and Negative Syndrome Scale | {1.0: 'absent', 2.0: 'minimal', 3.0: 'mild', 4.0: 'moderate', 5.0: 'moderate/severe', 6.0: 'severe', 7.0: 'extreem'} |
| V1pa22 | PANSS G8: 'Uncooperativeness' | Positive and Negative Syndrome Scale | {1.0: 'absent', 2.0: 'minimal', 3.0: 'mild', 4.0: 'moderate', 5.0: 'moderate/severe', 6.0: 'severe', 7.0: 'extreem'} |
| V1pa23 | PANSS G9: 'Unusual thought content' | Positive and Negative Syndrome Scale | {1.0: 'absent', 2.0: 'minimal', 3.0: 'mild', 4.0: 'moderate', 5.0: 'moderate/severe', 6.0: 'severe', 7.0: 'extreem'} |
| V1pa24 | PANSS G10: 'Disorientation' | Positive and Negative Syndrome Scale | {1.0: 'absent', 2.0: 'minimal', 3.0: 'mild', 4.0: 'moderate', 5.0: 'moderate/severe', 6.0: 'severe', 7.0: 'extreem'} |
| V1pa25 | PANSS G11: 'Poor attention' | Positive and Negative Syndrome Scale | {1.0: 'absent', 2.0: 'minimal', 3.0: 'mild', 4.0: 'moderate', 5.0: 'moderate/severe', 6.0: 'severe', 7.0: 'extreem'} |
| V1pa26 | PANSS G12: 'Lack of judgment and insight' | Positive and Negative Syndrome Scale | {1.0: 'absent', 2.0: 'minimal', 3.0: 'mild', 4.0: 'moderate', 5.0: 'moderate/severe', 6.0: 'severe', 7.0: 'extreem'} |
| V1pa27 | PANSS G13: 'Disturbance of volition' | Positive and Negative Syndrome Scale | {1.0: 'absent', 2.0: 'minimal', 3.0: 'mild', 4.0: 'moderate', 5.0: 'moderate/severe', 6.0: 'severe', 7.0: 'extreem'} |
| V1pa28 | PANSS G14: 'Poor impulse control' | Positive and Negative Syndrome Scale | {1.0: 'absent', 2.0: 'minimal', 3.0: 'mild', 4.0: 'moderate', 5.0: 'moderate/severe', 6.0: 'severe', 7.0: 'extreem'} |
| V1pa29 | PANSS G15: 'Preoccupation' | Positive and Negative Syndrome Scale | {1.0: 'absent', 2.0: 'minimal', 3.0: 'mild', 4.0: 'moderate', 5.0: 'moderate/severe', 6.0: 'severe', 7.0: 'extreem'} |
| V1pa30 | PANSS G16: 'Active Social Avoidance' | Positive and Negative Syndrome Scale | {1.0: 'absent', 2.0: 'minimal', 3.0: 'mild', 4.0: 'moderate', 5.0: 'moderate/severe', 6.0: 'severe', 7.0: 'extreem'} |
| V1ptotal | PANSS Positive total | Positive and Negative Syndrome Scale | Total positive score, 7-49 |
| V1ntotal | PANSS Negative total | Positive and Negative Syndrome Scale | Total negative score, 7-49 |
| V1gtotal | PANSS General total | Positive and Negative Syndrome Scale | Total general score, 16-112 |
| V1total | PANSS total | Positive and Negative Syndrome Scale | Total score, 30-210 |
| V1CGI_V1 | CGI: 'Severity of illness' | Clinical Global Impression | {1.0: 'Normal, not at all ill', 2.0: 'Borderline mentally ill', 3.0: 'Mildly ill', 4.0: 'Moderately ill', 5.0: 'Markedly ill', 6.0: 'Severely ill', 7.0: 'Among the most extremely ill patients'} |
| age | Age | Sociodemographic battery | Age in years |
| a003 | Sex | Sociodemographic battery | {1.0: 'Male', 2.0: 'Female'} |
| a015_t_1 | Treatment: 'inpatient' | Sociodemographic battery | {0.0: 'no', 1.0: 'yes'} |
| a015_t_2 | Treatment: 'outpatient' | Sociodemographic battery | {0.0: 'no', 1.0: 'yes'} |
| a015_t_3 | Treatment: 'day care' | Sociodemographic battery | {0.0: 'no', 1.0: 'yes'} |
| a015_t_4 | Treatment: 'other setting' | Sociodemographic battery | {0.0: 'no', 1.0: 'yes'} |
| a022_1 | Race: 'caucasian' | Sociodemographic battery | {0.0: 'no', 1.0: 'yes'} |
| a022_2 | Race: 'black' | Sociodemographic battery | {0.0: 'no', 1.0: 'yes'} |
| a022_3 | Race: 'asian' | Sociodemographic battery | {0.0: 'no', 1.0: 'yes'} |
| a030 | Employed (current) | Sociodemographic battery | {0.0: 'no', 1.0: 'yes'} |
| a041 | Living alone | Sociodemographic battery | {0.0: 'no', 1.0: 'yes'} |
| V1l05 | Cholesterol | Blood tests | Conc. in mg/dL |
| V1l06 | HDL | Blood tests | Conc. in mg/dL |
| V1l07 | LDL | Blood tests | Conc. in mg/dL |
| V1l09 | Triglycerides | Blood tests | Conc. in mg/dL |
| ph05 | Blood pressure (diastole) | Vitals | Pressure in mmHg |
| ph04 | Blood pressure (systole) | Vitals | Pressure in mmHg |
| ph06 | Pulse rate | Vitals | Pulse rate in bpm |
| phbmi | Body mass index | Vitals | weight/height^2 in IS units |
| job_sat | Job satisfaction | Manchester Short Assessment of Quality of Life & Quality of Life Survey | Harmonized from 0 (poor) to 1 (good) |
| rel_satisf | Intimate relationship satisfaction | Manchester Short Assessment of Quality of Life & Quality of Life Survey | Harmonized from 0 (poor) to 1 (good) |
| Leis_satisf | Leisure and social satisfaction | Manchester Short Assessment of Quality of Life & Quality of Life Survey | Harmonized from 0 (poor) to 1 (good) |
| house_satisf | Household satisfaction | Manchester Short Assessment of Quality of Life & Quality of Life Survey | Harmonized from 0 (poor) to 1 (good) |
| sociosexual_satisf | Sociosexual satisfaction | Manchester Short Assessment of Quality of Life & Quality of Life Survey | Harmonized from 0 (poor) to 1 (good) |
| gen_health_satisf | General health satisfaction | Manchester Short Assessment of Quality of Life & 12-Item Health Survey | Harmonized from 0 (poor) to 1 (good) |
| Ment_health_satisf | Mental health satisfaction | Manchester Short Assessment of Quality of Life & Mental Health Recovery Measure | Harmonized from 0 (poor) to 1 (good) |
| vm_tr1 | Verbal memory # correct answers trial 1 | Rey Auditory Verbal Learning Test | Total score 0-15 |
| vm_tr2 | Verbal memory # correct answers trial 2 | Rey Auditory Verbal Learning Test | Total score 0-15 |
| vm_tr3 | Verbal memory # correct answers trial 3 | Rey Auditory Verbal Learning Test | Total score 0-15 |
| vm_tr4 | Verbal memory # correct answers trial 4 | Rey Auditory Verbal Learning Test | Total score 0-15 |
| vm_tr5 | Verbal memory # correct answers trial 5 | Rey Auditory Verbal Learning Test | Total score 0-15 |
| vm_total | Verbal memory # total correct answers | Rey Auditory Verbal Learning Test | Total score 0-75 |
| sc_total | Digital symbol coding | Wechsler Adult Intelligence Scale, Third Edition | Number of correct symbols |
| hand_total | Motor coordination | Purdue pegboard & token motor task | Z score of each respective test distribution |
| exec_total | Executive functioning | Trail marking & Tower of London test | Z score of each respective test distribution |
| Risperidone | Risperidone treatment | Medication records | {0.0: 'no', 1.0: 'yes'} |
| Clozapine | Clozapine treatment | Medication records | {0.0: 'no', 1.0: 'yes'} |
| Olanzapine | Olanzapine treatment | Medication records | {0.0: 'no', 1.0: 'yes'} |
| Quetiapine | Quetiapine treatment | Medication records | {0.0: 'no', 1.0: 'yes'} |
| Sulpiride | Sulpiride treatment | Medication records | {0.0: 'no', 1.0: 'yes'} |
| Haloperidol | Haloperidol treatment | Medication records | {0.0: 'no', 1.0: 'yes'} |
| Zuclopenthixol | Zuclopenthixol treatment | Medication records | {0.0: 'no', 1.0: 'yes'} |
| Perphenazine | Perphenazine treatment | Medication records | {0.0: 'no', 1.0: 'yes'} |
| Chlorpromazine | Chlorpromazine treatment | Medication records | {0.0: 'no', 1.0: 'yes'} |
| Levomepromazine | Levomepromazine treatment | Medication records | {0.0: 'no', 1.0: 'yes'} |
| Amisulpride | Amisulpride treatment | Medication records | {0.0: 'no', 1.0: 'yes'} |
| Prothipendyl | Prothipendyl treatment | Medication records | {0.0: 'no', 1.0: 'yes'} |
| Clotiapine | Clotiapine treatment | Medication records | {0.0: 'no', 1.0: 'yes'} |
| Gabapentin | Gabapentin treatment | Medication records | {0.0: 'no', 1.0: 'yes'} |
| Perazine | Perazine treatment | Medication records | {0.0: 'no', 1.0: 'yes'} |
| Flupentixol | Flupentixol treatment | Medication records | {0.0: 'no', 1.0: 'yes'} |
| Penfluridol | Penfluridol treatment | Medication records | {0.0: 'no', 1.0: 'yes'} |
| Pimozide | Pimozide treatment | Medication records | {0.0: 'no', 1.0: 'yes'} |
| Promazine | Promazine treatment | Medication records | {0.0: 'no', 1.0: 'yes'} |
| Fluphenazine | Fluphenazine treatment | Medication records | {0.0: 'no', 1.0: 'yes'} |
| Loxapine | Loxapine treatment | Medication records | {0.0: 'no', 1.0: 'yes'} |
| Ziprasidone | Ziprasidone treatment | Medication records | {0.0: 'no', 1.0: 'yes'} |
| Chlorprothixene | Chlorprothixene treatment | Medication records | {0.0: 'no', 1.0: 'yes'} |
| Trifluoperazine | Trifluoperazine treatment | Medication records | {0.0: 'no', 1.0: 'yes'} |
| Cyamemazine | Cyamemazine treatment | Medication records | {0.0: 'no', 1.0: 'yes'} |
| Pipamperone | Pipamperone treatment | Medication records | {0.0: 'no', 1.0: 'yes'} |
| Zotepine | Zotepine treatment | Medication records | {0.0: 'no', 1.0: 'yes'} |
| Thioridazine | Thioridazine treatment | Medication records | {0.0: 'no', 1.0: 'yes'} |
| Prochlorperazine | Prochlorperazine treatment | Medication records | {0.0: 'no', 1.0: 'yes'} |
| Antidepressant | Antidepressant treatment | Medication records | {0.0: 'no', 1.0: 'yes'} |
| diag_1 | Schizophrenia | Diagnosis | {0.0: 'no', 1.0: 'yes'} |
| diag_2 | Schizophreniform disorder | Diagnosis | {0.0: 'no', 1.0: 'yes'} |
| diag_3 | Schizoaffective disorder | Diagnosis | {0.0: 'no', 1.0: 'yes'} |
| diag_4 | Other psychotic disorder | Diagnosis | {0.0: 'no', 1.0: 'yes'} |

Abbreviations: CDSS, Calgary depression scale for schizophrenia; PANSS, positive and negative syndrome scale; CGI, clinical global impression;

Table S5. Statistical comparisons of baseline variables between EUFEST patients included and excluded in the study.

| **Variables** | **Included** | **Excluded** | **t/χ^2^ (FDR-corrected p value)** |
| --- | --- | --- | --- |
| **Sociodemographics** | | | |
| Sample, n | 320 | 164 | - |
| Age in years, Mean (SD) | 25.93 (5.60) | 25.86 (5.43) | t_483_ = 0.13 (0.985) |
| Sex (male), n | 181 (56.56%) | 109 (66.46%) | $\chi_{1}^{2}$ = 4.02 (0.516) |
| Employed, n | 151 (47.18%) | 74 (45.68%) | $\chi_{1}^{2}$ = 0.05 (0.985) |
| BMI, Mean (SD) | 22.04 (3.17) | 22.60 (3.92) | t_469_ = -1.51 (0.553) |
| *Race, n* | | | |
| White, n | 307 (95.93%) | 149 (90.85%) | $\chi_{2}^{2}=$ 5.26 (0.553) |
| Black, n | 7 (2.18%) | 9 (5.48%) |
| Asian, n | 1 (0.31%) | 1 (0.61%) |
| **Psychopathology** | | | |
| PANSS positive score, Mean (SD) | 23.34 (6.16) | 23.05 (6.32) | t_483_ = 0.47 (0.960) |
| PANSS negative score, Mean (SD) | 21.22 (10.77) | 21.77 (7.37) | t_483_ = -0.76 (0.877) |
| PANSS general score, Mean (SD) | 44.53 (10.77) | 43.89 (10.92) | t_483_ = 0.60 (0.898) |
| CGI, Mean (SD) | 4.85 (0.76) | 4.86 (0.82) | t_483_ = -0.17 (0.985) |
| CDSS, Mean (SD) | 5.2 (4.85) | 4.87 (4.97) | t_483_ = 0.68 (0.877) |
| **Substance use** | | | |
| Alcohol use, n | 111 (34.69%) | 60 (36.59%) | $\chi_{1}^{2}$ = 0.10 (0.96) |
| Cannabis use, n | 71 (22.18%) | 49 (29.88%) | $\chi_{1}^{2}$ = 3.04 (0.553) |
| Other substance use, n | 27 (8.43%) | 21 (12.81%) | $\chi_{1}^{2}=$ 1.851 (0.553) |
| **Diagnosis** | | | |
| *Diagnose, n* | | | |
| Schizophrenia, n | 163 (50.93%) | 90 (50.94%) | $\chi_{3}^{2}=$ 4.07 (0.877) |
| Schizophreniform disorder, n | 0 (0%) | 1 (0.06%) |
| Schizoaffective disorder, n | 24 (7.50%) | 8 (5%) |
| Other psychotic disorder, n | 128 (40.63%) | 61 (38.12%) |
| **Medication** | | | |
| Antidepressant prescription, n | 7 (2.18%) | 4 (2.44%) | $\chi_{1}^{2}$ = 0.00 (1.000) |

Abbreviations: BMI, body mass index (calculated as weight in kilograms divided by square of height in meters); PANSS, positive and negative syndrome scale; CGI, clinical global impression; CDSS, Calgary depression scale for schizophrenia; EUFEST, European first episode schizophrenia trial.

Table S6. Statistical comparisons of baseline variables between RAISE-ETP patients included and excluded in the study.

| **Variables** | **Included** | **Excluded** | **t/χ^2^ (FDR-corrected p value)** |
| --- | --- | --- | --- |
| **Sociodemographics** | | | |
| Sample, n | 234 | 170 | - |
| Age in years, Mean (SD) | 23.90 (5.27) | 23.23 (4.76) | t_402_ = 1.32 (0.433) |
| Sex (male), n | 172 (73.50%) | 120 (71.00%) | $\chi_{1}^{2}$ = 0.19 (0.821) |
| Employed, n | 31 (13.24%) | 27 (15.98%) | $\chi_{1}^{2}$ = 0.39 (0.821) |
| BMI, Mean (SD) | 27.06 (6.85) | 25.69 (6.26) | t_388_ = 2.04 (0.261) |
| *Race, n* | | | |
| White, n | 135 (57.69%) | 83 (50.88%) | $\chi_{2}^{2}=$ 9.09 (0.26) |
| Black, n | 79 (33.76%) | 73 (43.20%) |
| Asian, n | 4 (1.70%) | 7 (4.14%) |
| **Psychopathology** | | | |
| PANSS positive score, Mean (SD) | 18.84 (5.22) | 18.67 (5.24) | t_402_ = 0.31 (0.821) |
| PANSS negative score, Mean (SD) | 18.84 (5.22) | 20.33 (5.34) | t_402_ = -0.44 (0.821) |
| PANSS general score, Mean (SD) | 20.09 (5.30) | 38.23 (8.93) | t_402_ = -1.18 (0.465) |
| CGI, Mean (SD) | 4.06 (0.83) | 4.03 (0.80) | t_402_ = 0.42 (0.821) |
| CDSS, Mean (SD) | 4.16 (3.79) | 5.33 (4.81) | t_402_ = -2.60 (0.223) |
| **Substance use** | | | |
| Alcohol use, n | 63 (26.92%) | 50 (29.94%) | $\chi_{1}^{2}$ = 0.30 (0.821) |
| Cannabis use, n | 53 (22.65%) | 42 (25.15%) | $\chi_{1}^{2}$ = 0.21 (0.821) |
| Other substance use, n | 5 (2.13%) | 5 (2.99%) | $\chi_{1}^{2}=$ 0.04 (0.833) |
| **Diagnosis** | | | |
| *Diagnose, n* | | | |
| Schizophrenia, n | 134 (57.26%) | 79 (46.75%) | $\chi_{3}^{2}=$ 6.34 (0.433) |
| Schizophreniform disorder, n | 37 (15.81%) | 30 (17.64%) |
| Schizoaffective disorder, n | 40 (17.09%) | 41 (24.11%) |
| Other psychotic disorder, n | 23 (9.82%) | 19 (11.18%) |
| **Medication** | | | |
| Antidepressant prescription, n | 78 (33.33%) | 60 (35.29%) | $\chi_{1}^{2}$ = 0.09 (0.821) |

Abbreviations: BMI, body mass index (calculated as weight in kilograms divided by square of height in meters); PANSS, positive and negative syndrome scale; CGI, clinical global impression; CDSS, Calgary depression scale for schizophrenia; RAISE-ETP, recovery after an initial schizophrenia episode early treatment program.

Table S7. Statistical comparisons of variables between patients included in the study from EUFEST and RAISE-ETP samples.

| **Variables** | **EUFEST baseline** | **RAISE-ETP baseline** | **t/χ^2^ (FDR-corrected p value)** |
| --- | --- | --- | --- |
| **Sociodemographics** | | | |
| Sample, n | 320 | 234 | - |
| Age in years, Mean (SD) | 25.93 (5.60) | 23.90 (5.27) | t_553_ = 4.36 (<0.001) |
| Sex (male), n | 181 (56.56%) | 172 (73.50%) | $\chi_{1}^{2}$ = 16.06 (0.001) |
| Employed, n | 151 (47.18%) | 31 (13.24%) | $\chi_{1}^{2}$ = 69.05 (<0.001) |
| Body mass index (BMI), Mean (SD) | 22.04 (3.17) | 27.06 (6.85) | t_546_ = - 10.30 (<0.001) |
| *Race, n* | | | |
| White, n | 307 (95.93%) | 135 (57.69%) | $\chi_{2}^{2}=$ 124.42 (<0.001) |
| Black, n | 7 (2.18%) | 79 (33.76%) |
| Asian, n | 1 (0.31%) | 4 (1.70%) |
| **Psychopathology** | | | |
| PANSS positive score, Mean (SD) | 23.34 (6.16) | 18.84 (5.22) | t_553_ = 9.28 (<.001) |
| PANSS negative score, Mean (SD) | 21.22 (10.77) | 18.84 (5.22) | t_553_ = 2.03 (0.052) |
| PANSS general score, Mean (SD) | 44.53 (10.77) | 20.09 (5.30) | t_553_ = 9.47 (<.001) |
| CGI, Mean (SD) | 4.85 (0.76) | 4.06 (0.83) | t_553_ = 11.41 (<.001) |
| CDSS, Mean (SD) | 5.2 (4.85) | 4.16 (3.79) | t_553_ = 2.81 (0.007) |
| **Substance use** | | | |
| Alcohol use, n | 111 (34.69%) | 63 (26.92%) | $\chi_{1}^{2}$ = 3.43 (0.073) |
| Cannabis use, n | 71 (22.18%) | 53 (22.65%) | $\chi_{1}^{2}$ = 0.0006 (0.979) |
| Other substance use, n | 27 (8.43%) | 5 (2.13%) | $\chi_{1}^{2}=$ 8.67 (0.005) |
| **Diagnosis** | | | |
| *Diagnose, n* | | | |
| Schizophrenia, n | 163 (50.93%) | 134 (57.26%) | $\chi_{3}^{2}=$ 111.17 (<.001) |
| Schizophreniform disorder, n | 0 (0%) | 37 (15.81%) |
| Schizoaffective disorder, n | 24 (7.50%) | 40 (17.09%) |
| Other psychotic disorder, n | 128 (40.63%) | 23 (9.82%) |
| **Medication** | | | |
| Antidepressant prescription, n | 7 (2.18%) | 78 (33.33%) | $\chi_{1}^{2}$ = 98.56 (<.001) |

Abbreviations: BMI, body mass index (calculated as weight in kilograms divided by square of height in meters); PANSS, positive and negative syndrome scale; CGI, clinical global impression; CDSS, Calgary depression scale for schizophrenia; EUFEST, European first episode schizophrenia trial; RAISE-ETP, recovery after an initial schizophrenia episode early treatment program.

Table S8. Performance metrics of regressors and classifiers using all available variables at baseline.

| Sample | Label | Regressors | | Classifiers | | | | | | | | | | |
| --- | --- | --- | --- | --- | --- | --- | --- | --- | --- | --- | --- | --- | --- | --- |
|  |  | r (p value) | R^2^ (%) | TP | TN | FP | FN | Sens. | Spec. | BAC | PPV | NPV | PSI | AUC-ROC |
| EUFEST (all variables) | ±ΔCDSS 1 month | 0.65 (<.001) | 41.68 | 100 | 135 | 51 | 34 | 74.63 | 72.58 | 73.60 | 66.22 | 79.88 | 46.10 | 0.83 |
|  | ±ΔCDSS 6 months | 0.81 (<.001) | 65.50 | 80 | 167 | 48 | 25 | 76.19 | 77.67 | 76.93 | 62.5 | 86.98 | 49.48 | 0.87 |
|  | ±ΔCDSS 12 months | 0.88 (<.001) | 77.53 | 78 | 183 | 45 | 14 | 84.78 | 80.26 | 82.52 | 63.41 | 92.89 | 56.31 | 0.88 |
|  | ± PPD | - | - | 49 | 171 | 70 | 30 | 62.03 | 70.95 | 66.49 | 41.18 | 85.07 | 26.25 | 0.80 |
| RAISE-ETP (all variables) | +/- ΔCDSS 6 months | 0.5 (<.001) | 24.52 | 78 | 83 | 33 | 40 | 66.10 | 71.55 | 68.83 | 70.27 | 67.47 | 37.75 | 0.76 |
|  | ±ΔCDSS 12 months | 0.53 (<.001) | 28.06 | 97 | 60 | 32 | 45 | 68.31 | 65.21 | 66.76 | 75.19 | 57.14 | 32.33 | 0.74 |
|  | ± PPD | - | - | 26 | 144 | 45 | 19 | 57.78 | 76.19 | 66.98 | 36.62 | 88.34 | 24.96 | 0.74 |

Abbreviations: TP, true positive; TN, true negative; FP, false positive; FN, false negative; sens., sensitivity; spec., specificity; BAC, balanced accuracy; PPV, positive predictive value; NPV, negative predictive value; PSI, prognostic summary index; AUC-ROC, area under the receiver operating characteristic curve*;* EUFEST, European first episode schizophrenia trial; RAISE-ETP, recovery after an initial schizophrenia episode early treatment program; CDSS, Calgary depression scale for schizophrenia; PPD, post-psychotic depression.

Table S9. Performance metrics of regressors and classifiers in discovery and out of sample validation using leave-site-out cross-validation.

| Sample | Label | Regressors | | Classifiers | | | | | | | | | | |
| --- | --- | --- | --- | --- | --- | --- | --- | --- | --- | --- | --- | --- | --- | --- |
|  |  | r (p value) | R^2^ (%) | TP | TN | FP | FN | Sens. | Spec. | BAC | PPV | NPV | PSI | AUC-ROC |
| EUFEST OOT | ± ΔCDSS 6 months | 0.81 (<.001) | 65.90 | 87 | 144 | 71 | 18 | 82.86 | 66.98 | 74.92 | 55.06 | 88.88 | 43.95 | 0.83 |
|  | ± ΔCDSS 12 months | 0.88 (<.001) | 77.06 | 80 | 156 | 72 | 12 | 86.96 | 68.42 | 77.69 | 52.63 | 92.86 | 45.49 | 0.84 |
|  | ± PPD | - | - | 51 | 167 | 74 | 28 | 64.56 | 69.29 | 66.93 | 40.80 | 85.64 | 26.44 | 0.74 |
| EUFEST OOCV | ± ΔCDSS 6 months | 0.78 (<.001) | 61.41 | 97 | 116 | 99 | 8 | 92.38 | 53.95 | 73.17 | 49.49 | 93.55 | 43.04 | 0.87 |
|  | ± ΔCDSS 12 months | 0.83 (<.001) | 69.45 | 82 | 155 | 73 | 10 | 89.13 | 67.98 | 78.56 | 52.90 | 93.93 | 46.84 | 0.88 |
|  | ± PPD | - | - | 41 | 161 | 80 | 38 | 51.90 | 66.80 | 59.35 | 33.88 | 80.90 | 14.79 | 0.66 |
| RAISE-ETP  OOT | ± ΔCDSS 6 months | 0.55 (<.001) | 30.14 | 92 | 64 | 54 | 24 | 79.31 | 54.24 | 66.77 | 63.01 | 72.73 | 35.74 | 0.77 |
|  | ± ΔCDSS 12 months | 0.59 (<.001) | 34.67 | 69 | 75 | 67 | 23 | 61.54 | 52.82 | 63.91 | 50.74 | 76.53 | 27.27 | 0.73 |
|  | ± PPD | - | - | 27 | 125 | 64 | 18 | 60.00 | 66.14 | 63.07 | 29.67 | 87.41 | 17.08 | 0.68 |
| RAISE-ETP OOCV | ± ΔCDSS 6 months | 0.50 (<.001) | 24.57 | 79 | 77 | 41 | 37 | 68.10 | 65.25 | 66.68 | 65.83 | 67.54 | 33.38 | 0.74 |
|  | ± ΔCDSS 12 months | 0.59 (<.001) | 35.06 | 66 | 90 | 52 | 26 | 71.74 | 63.38 | 67.56 | 55.93 | 77.59 | 33.52 | 0.75 |
|  | ± PPD | - | - | 22 | 148 | 41 | 23 | 48.88 | 78.31 | 63.60 | 34.92 | 86.55 | 21.47 | 0.70 |

Abbreviations: TP, true positive; TN, true negative; FP, false positive; FN, false negative; sens., sensitivity; spec., specificity; BAC, balanced accuracy; PPV, positive predictive value; NPV, negative predictive value; PSI, prognostic summary index; AUC-ROC, area under the receiver operating characteristic curve*;* EUFEST, European first episode schizophrenia trial; RAISE-ETP, recovery after an initial schizophrenia episode early treatment program; CDSS, Calgary depression scale for schizophrenia; PPD, post-psychotic depression; OOT, out-of-training; OOCV, out-of-cross-validation.

Table S10. Performance metrics of regressors and classifiers in discovery and out of sample validation without psychopathology questionnaire total scores.

| Sample | Label | Regressors | | Classifiers | | | | | | | | | | |
| --- | --- | --- | --- | --- | --- | --- | --- | --- | --- | --- | --- | --- | --- | --- |
|  |  | r (p value) | R^2^ (%) | TP | TN | FN | FP | Sens. | Spec. | BAC | PPV | NPV | PSI | AUC-ROC |
| EUFEST OOT | ± ΔCDSS 6 months | 0.81 (<.001) | 66.01 | 85 | 149 | 20 | 66 | 80.95 | 69.30 | 75.13 | 56.29 | 88.17 | 44.46 | 0.81 |
|  | ± ΔCDSS 12 months | 0.88 (<.001) | 76.69 | 78 | 159 | 14 | 69 | 84.78 | 69.74 | 77.26 | 53.06 | 91.91 | 44.97 | 0.84 |
|  | ± PPD | - | - | 51 | 174 | 28 | 67 | 64.56 | 72.20 | 68.38 | 43.22 | 86.14 | 29.36 | 0.75 |
| EUFEST OOCV | ± ΔCDSS 6 months | 0.78 (<.001) | 60.76 | 94 | 120 | 11 | 95 | 89.52 | 55.81 | 72.67 | 49.74 | 91.60 | 41.34 | 0.83 |
|  | ± ΔCDSS 12 months | 0.83 (<.001) | 68.49 | 78 | 144 | 14 | 84 | 84.78 | 63.16 | 73.97 | 48.15 | 91.14 | 39.29 | 0.84 |
|  | ± PPD | - | - | 41 | 158 | 38 | 83 | 51.90 | 65.56 | 58.73 | 33.06 | 80.61 | 13.68 | 0.67 |
| RAISE-ETP  OOT | ± ΔCDSS 6 months | 0.49 (<.001) | 23.84 | 77 | 69 | 39 | 49 | 66.38 | 58.47 | 62.43 | 63.89 | 61.11 | 25.00 | 0.70 |
|  | ± ΔCDSS 12 months | 0.59 (<.001) | 34.50 | 80 | 51 | 12 | 90 | 86.96 | 36.17 | 61.56 | 47.06 | 80.95 | 28.01 | 0.72 |
|  | ± PPD | - | - | 19 | 147 | 26 | 42 | 42.22 | 77.78 | 60.00 | 31.15 | 84.97 | 16.12 | 0.70 |
| RAISE-ETP OOCV | ± ΔCDSS 6 months | 0.55 (<.001) | 30.04 | 94 | 67 | 22 | 51 | 81.03 | 56.78 | 68.91 | 64.83 | 75.28 | 40.11 | 0.72 |
|  | ± ΔCDSS 12 months | 0.58 (<.001) | 33.74 | 65 | 83 | 27 | 59 | 70.65 | 58.45 | 64.55 | 52.42 | 75.45 | 27.87 | 0.71 |
|  | ± PPD | - | - | 26 | 135 | 19 | 54 | 57.78 | 71.43 | 64.60 | 32.50 | 87.66 | 20.16 | 0.70 |

Abbreviations: TP, true positive; TN, true negative; FP, false positive; FN, false negative; sens., sensitivity; spec., specificity; BAC, balanced accuracy; PPV, positive predictive value; NPV, negative predictive value; PSI, prognostic summary index; AUC-ROC, area under the receiver operating characteristic curve*;* EUFEST, European first episode schizophrenia trial; RAISE-ETP, recovery after an initial schizophrenia episode early treatment program; CDSS, Calgary depression scale for schizophrenia; PPD, post-psychotic depression; OOT, out-of-training; OOCV, out-of-cross-validation.

Table S11. Performance metrics of classifiers in the prediction of negative symptoms changes and remission from psychosis.

| Sample | Label | BAC | Perm. p value |
| --- | --- | --- | --- |
| EUFEST | ± ΔPANSS_NEG subscale 6 months | 44.95 | 0.987 |
|  | ± ΔPANSS_NEG Marder scale 6 months | 52.69 | 0.190 |
|  | ± ΔPANSS_NEG subscale 12 months | 48.58 | 0.880 |
|  | ± ΔPANSS_NEG Marder scale 12 months | 51.62 | 0.132 |
|  | ± PANSS_NEG subscale | 52.20 | 0.088 |
|  | ± PANSS_NEG Marder scale | 53.52 | 0.010 |
|  | ± PANSS_POS_50% | 50.15 | 0.501 |
| RAISE-ETP | ± ΔPANSS_NEG subscale 6 months | 54.73 | 0.106 |
|  | ± ΔPANSS_NEG Marder scale 6 months | 50.57 | 0.450 |
|  | ± ΔPANSS_NEG subscale 12 months | 52.24 | 0.301 |
|  | ± ΔPANSS_NEG Marder scale 12 months | 49.93 | 0.539 |
|  | ± PANSS_NEG subscale | 53.01 | 0.001 |
|  | ± PANSS_NEG Marder scale | 53.17 | 0.002 |
|  | ± PANSS_POS_50% | 54.37 | <0.001 |

Abbreviations: BAC balanced accuracy; EUFEST, European first episode schizophrenia trial; RAISE-ETP, recovery after an initial schizophrenia episode early treatment program; PANSS, positive and negative syndrome scale.

Table S12. Mixed-design ANOVA of depression scores by treatment group and visit in the RAISE-ETP sample.

| Source | SS | DF1 | DF2 | MS | F | p-value |
| --- | --- | --- | --- | --- | --- | --- |
| NAVIGATE : CC | 171.23 | 1 | 232 | 171.23 | 6.71 | 0.010 |
| Visit | 314.78 | 2 | 464 | 157.39 | 17.33 | <.001 |
| Interaction | 54.49 | 2 | 2 | 27.259 | 3.00 | 0.050 |

Abbreviations: DF1, degrees of freedom 1; DF2, degrees of freedom 2; SS, sum of squares; MS, mean square; F, F-statistic; CC, community care treatment plan.

Table S3. Mixed-design ANOVA of depression scores by antidepressant treatment and visit in the RAISE-ETP sample.

| Source | SS | DF1 | DF2 | MS | F | p-value |
| --- | --- | --- | --- | --- | --- | --- |
| +AD : -AD | 194.08 | 1 | 232 | 194.08 | 7.64 | 0.006 |
| Visit | 314.78 | 2 | 464 | 157.39 | 17.24 | <.001 |
| Interaction | 33.00 | 2 | 464 | 16.50 | 1.81 | 0.165 |

Abbreviations: DF1, degrees of freedom 1; DF2, degrees of freedom 2; SS, sum of squares; MS, mean square; F, F-statistic; ±AD, antidepressant treatment (yes or no).

Table S14. Mixed-design ANOVA of depression scores by antidepressant type and visit for AD treated patients in RAISE-ETP sample.

| Source | SS | DF1 | DF2 | MS | F | p-value |
| --- | --- | --- | --- | --- | --- | --- |
| AD type | 113.31 | 4 | 90 | 28.33 | 0.82 | 0.518 |
| Visit | 229.52 | 2 | 180 | 114.76 | 10.32 | <.001 |
| Interaction | 55.27 | 8 | 180 | 6.91 | 0.62 | 0.759 |

Abbreviations: DF1, degrees of freedom 1; DF2, degrees of freedom 2; SS, sum of squares; MS, mean square; F, F-statistic; AD type: antidepressant type, including the following: TCA, tricyclic antidepressants; NDRI, norepinephrine-dopamine reuptake inhibitors; SSRI, selective serotonin reuptake inhibitors; SNRI, serotonin-norepinephrine reuptake inhibitors; SARI, serotonin antagonist and reuptake inhibitors.

| Model: Mixed Linear Model  N observations: 702  N groups: 33  Min. group size: 3  Max. group size: 57  Mean group size: 21.3 | | Dependent variable: CDSS total  Method: REML  Scale: 13.40  Log-Likelihood: -1924.18  Converged: Yes | | | |
| --- | --- | --- | --- | --- | --- |
| Variable | Coefficient | Std. Err. | z | P>|z| | C.I |
| Intercept | 5.91 | 0.67 | 8.78 | <.001 | [4.59, 7.22] |
| Antidepressant (- AD) | -1.58 | 0.46 | -3.5 | 0.001 | [-2.48, -0.68] |
| Treatment (NAVIGATE) | -1.47 | 0.67 | -2.21 | 0.027 | [-2.78, -0.17] |
| Long-acting injectables (- LAI) | 0.11 | 0.47 | 0.24 | 0.813 | [-0.82, 1.04] |
| Antidepressant (- AD): Treatment (NAVIGATE) | 0.95 | 0.63 | 1.52 | 0.130 | [-0.28, 2.19] |
| Visit | -0.18 | 0.04 | -4.39 | <.001 | [-0.26, -0.10] |
| Group Var | 1.45 | 0.18 | - | - | - |

Table S15. Mixed linear model regression of CDSS scores considering antidepressant prescription, treatment plan and long-acting injectable prescription.

Abbreviations: CDSS, Calgary depression scale for schizophrenia; REML, restricted maximum likelihood; C.I., confidence interval; LAI, long-acting injectable; AD, antidepressant.

| Model: Mixed Linear Model  N observations: 171  N groups: 24  Min. group size: 3  Max. group size: 15  Mean group size: 7.1 | | Dependent variable: CDSS total  Method: REML  Scale: 16.33  Log-Likelihood: -482.22  Converged: Yes | | | |
| --- | --- | --- | --- | --- | --- |
| Variable | Coefficient | Std. Err. | z | P>|z| | C.I |
| Intercept | 14.45 | 1.35 | 10.68 | <.001 | [11.79, 17.10] |
| Antidepressant (- AD) | -2.25 | 1.04 | -2.17 | 0.030 | [-4.28, -0.22] |
| Treatment (NAVIGATE) | -2.62 | 1.12 | -2.33 | 0.020 | [-4.82, -0.42] |
| Long-acting injectables (- LAI) | -2.37 | 1.05 | -2.26 | 0.024 | [-4.41, -0.32] |
| Antidepressant (- AD): Treatment (NAVIGATE) | 1.94 | 1.43 | 1.36 | 0.175 | [-0.86, 4.74] |
| Visit | -0.65 | 0.09 | -7.17 | <.001 | [-0.83, -0.47] |
| Group Var | 1.49 | 0.39 | - | - | - |

Table S16. Mixed linear model regression of CDSS scores considering antidepressant prescription, treatment plan and long-acting injectable prescription for RAISE-ETP patients with CDSS ≥ 7 at baseline.

Abbreviations: CDSS, Calgary depression scale for schizophrenia; REML, restricted maximum likelihood; C.I., confidence interval; LAI, long-acting injectable; AD, antidepressant.

Table S17. Comparison of misclassifications across different ethnicities and races in RAISE-ETP patients.

| Outcome label | Group | Χ^2^ statistic | p-value, FDR corrected |
| --- | --- | --- | --- |
| ±ΔCDSS (6 months) | Race | $\chi_{4}^{2}=$ 3.573 | 0.900 |
|  | Ethnicity | $\chi_{1}^{2}=$ 0.015 | 0.902 |
| ±ΔCDSS (12 months) | Race | $\chi_{4}^{2}=$ 5.695 | 0.900 |
|  | Ethnicity | $\chi_{1}^{2}=$ 0.102 | 0.900 |
| ±PPD | Race | $\chi_{4}^{2}=$ 4.176 | 0.900 |
|  | Ethnicity | $\chi_{1}^{2}=$ 0.158 | 0.900 |

Ethnicity included as categories: Hispanic or Latino and not Hispanic or Latino. Race included as categories: American Indian or Alaska Native, Asian, Black or African American, Native Hawaiian or Other Pacific Islander, and White. Abbreviations: CDSS, Calgary depression scale for schizophrenia; RAISE-ETP, recovery after an initial schizophrenia episode early treatment program; FDR, false discovery rate.

Table S18. Comparison of models’ performance when trained with all harmonized variables and a condensed set of top predictors.

| Outcome label | Sample | Baseline variables, n | Sens. | Spec. | BAC (p label perm.) | Wilcoxon statistic (p value, FDR corrected) |
| --- | --- | --- | --- | --- | --- | --- |
| ±ΔCDSS (6 months) | EUFEST OOCV | 123 (all) | 85.71 | 66.98 | 76.35 (<.001) | W = 20568.5 (0.299) |
|  |  | 24 | 83.80 | 66.97 | 75.39 (<.001) |  |
|  | RAISE OOCV | 123 (all) | 68.10 | 64.41 | 66.26 (<.001) | W = 6195.0 (<.001) |
|  |  | 20 | 79.31 | 57.62 | 68.46 (<.001) |  |
| ±ΔCDSS (12 months) | EUFEST OOCV | 123 (all) | 68.48 | 77.63 | 73.05 (0.026) | W = 5911.0 (<.001) |
|  |  | 28 | 60.08 | 83.69 | 71.89 (<.001) |  |
|  | RAISE OOCV | 123 (all) | 70.65 | 66.90 | 68.78 (<.001) | W = 3872.0 (<.001) |
|  |  | 24 | 81.52 | 55.63 | 68.57 (<.001) |  |
| ±PPD | EUFEST OOCV | 123 (all) | 49.37 | 68.46 | 58.92 (<.001) | W = 22049.5 (0.116) |
|  |  | 8 | 58.22 | 61.41 | 59.81 (<.001) |  |
|  | RAISE OOCV | 123 (all) | 40.0 | 78.31 | 59.15 (<.001) | W = 5156.0 (0.100) |
|  |  | 10 | 51.11 | 77.24 | 64.17 (<.001) |  |

Most relevant variables were defined as those with a feature selection of 50% or higher, and the number of variables is provided. To test for the differences in predictions using all the harmonized variables and the condensed version, the predicted OOCV labels were tested for significance using the Wilcoxon signed-rank test of the model probability scores. : BAC, balanced accuracy; CDSS, Calgary depression scale for schizophrenia; EUFEST, European first episode schizophrenia trial; FDR, false discovery rate; OOCV, out-of-cross-validation; OOT, out-of-training; PPD, post-psychotic depression; RAISE-ETP, recovery after an initial schizophrenia episode early treatment program; sens., sensitivity; spec., specificity.

Table S19. Performance metrics of regressors and classifiers in discovery and out of sample validation using only biological variables.

| Sample | Label | Regressors | | Classifiers | | | | | | | | | | | |
| --- | --- | --- | --- | --- | --- | --- | --- | --- | --- | --- | --- | --- | --- | --- | --- |
|  |  | r (p value) | R^2^ | TP | TN | FP | FN | Sens. | Spec. | BAC | PPV | NPV | PSI | AUC-ROC | Perm. p value |
| EUFEST OOT | ± ΔCDSS 6 months | 0.01 (0.833) | 0.01 | 48 | 64 | 117 | 34 | 58.53 | 35.35 | 46.94 | 29.09 | 65.30 | -5.60 | 0.41 | 0.831 |
|  | ± ΔCDSS 12 months | -0.00 (0.984) | 0.00 | 42 | 100 | 112 | 40 | 51.21 | 47.17 | 49.19 | 27.27 | 71.42 | -1.29 | 0.46 | 0.625 |
|  | ± PPD | - | - | 45 | 151 | 90 | 34 | 56.96 | 62.65 | 59.80 | 33.33 | 81.62 | 14.95 | 0.60 | <.001 |
| EUFEST OOCV | ± ΔCDSS 6 months | -0.06 (0.249) | 0.40 | 18 | 179 | 36 | 87 | 17.14 | 83.25 | 50.19 | 33.33 | 67.29 | 0.62 | 0.47 | 0.141 |
|  | ± ΔCDSS 12 months | -0.07 (0.207) | 0.50 | 25 | 165 | 63 | 67 | 27.17 | 72.36 | 49.77 | 28.40 | 71.12 | -0.47 | 0.49 | 0.502 |
|  | ± PPD | - | - | 24 | 197 | 44 | 55 | 30.37 | 81.74 | 56.06 | 35.29 | 78.17 | 13.46 | 0.55 | <.001 |
| RAISE-ETP  OOT | ± ΔCDSS 6 months | -0.04 (0.573) | 0.10 | 39 | 81 | 37 | 77 | 33.62 | 68.64 | 51.13 | 51.31 | 51.26 | 2.58 | 0.53 | 0.414 |
|  | ± ΔCDSS 12 months | -0.08 (0.179) | 0.78 | 43 | 82 | 60 | 49 | 46.73 | 57.74 | 52.24 | 41.74 | 62.59 | 4.34 | 0.52 | 0.235 |
|  | ± PPD | - | - | 28 | 125 | 64 | 17 | 62.22 | 66.14 | 64.18 | 30.43 | 88.03 | 18.46 | 0.69 | <.001 |
| RAISE-ETP OOCV | ± ΔCDSS 6 months | 0.04 (0.754) | -0.02 | 89 | 15 | 95 | 15 | 85.57 | 13.63 | 49.60 | 48.37 | 50.0 | -1.63 | 0.54 | 0.879 |
|  | ± ΔCDSS 12 months | -0.02 (0.771) | 0.03 | 57 | 49 | 81 | 28 | 67.05 | 37.69 | 52.37 | 41.30 | 63.63 | 4.94 | 0.51 | 0.120 |
|  | ± PPD | - | - | 29 | 92 | 97 | 16 | 64.44 | 48.67 | 56.56 | 23.01 | 85.18 | 8.20 | 0.57 | 0.087 |

Models were trained using biological-only variables, without a feature selection method but otherwise using the same methodology. Biological variables included V1l05, V1l06, V1l07, V1l09, ph05, ph04, ph06 and phbmi. Abbreviations: TP, true positive; TN, true negative; FP, false positive; FN, false negative; sens., sensitivity; spec., specificity; BAC, balanced accuracy; PPV, positive predictive value; NPV, negative predictive value; PSI, prognostic summary index; AUC-ROC, area under the receiver operating characteristic curve*;* EUFEST, European first episode schizophrenia trial; RAISE-ETP, recovery after an initial schizophrenia episode early treatment program; CDSS, Calgary depression scale for schizophrenia; PPD, post-psychotic depression; OOT, out-of-training; OOCV, out-of-cross-validation.

Table S20. Performance metrics of regressors and classifiers in discovery and out of sample validation using all harmonized variables but excluding biological variables.

| Sample | Label | Regressors | | Classifiers | | | | | | | | | | | |
| --- | --- | --- | --- | --- | --- | --- | --- | --- | --- | --- | --- | --- | --- | --- | --- |
|  |  | r (p value) | R^2^ | TP | TN | FP | FN | Sens. | Spec. | BAC | PPV | NPV | PSI | AUC-ROC | Perm. p value |
| EUFEST OOT | ± ΔCDSS 6 months | 0.82 (<.001) | 67.33 | 80 | TN | 60 | 25 | 76.19 | 72.09 | 74.14 | 57.14 | 86.11 | 43.25 | 0.82 | <.001 |
|  | ± ΔCDSS 12 months | 0.88 (<.001) | 77.07 | 76 | 155 | 67 | 16 | 82.60 | 70.61 | 76.61 | 53.14 | 90.96 | 44.11 | 0.84 | <.001 |
|  | ± PPD | - | - | 51 | 161 | 70 | 28 | 64.55 | 70.95 | 67.76 | 41.15 | 85.92 | 28.07 | 0.74 | <.001 |
| EUFEST OOCV | ± ΔCDSS 6 months | 0.76 (<.001) | 58.11 | 92 | 171 | 79 | 13 | 87.62 | 63.26 | 75.44 | 53.80 | 91.27 | 45.07 | 0.84 | <.001 |
|  | ± ΔCDSS 12 months | 0.80 (<.001) | 64.79 | 56 | 136 | 42 | 36 | 60.86 | 81.58 | 71.22 | 57.14 | 83.78 | 40.93 | 0.82 | <.001 |
|  | ± PPD | - | - | 42 | 186 | 77 | 37 | 53.16 | 68.05 | 60.61 | 81.59 | 35.29 | 16.89 | 0.68 | <.001 |
| RAISE-ETP  OOT | ± ΔCDSS 6 months | 0.50 (<.001) | 25.30 | 85 | 164 | 48 | 31 | 73.28 | 59.32 | 66.30 | 63.91 | 69.31 | 33.21 | 0.74 | <.001 |
|  | ± ΔCDSS 12 months | 0.55 (<.001) | 30.44 | 60 | 70 | 52 | 32 | 65.21 | 63.38 | 64.30 | 53.57 | 73.77 | 27.34 | 0.68 | <.001 |
|  | ± PPD | - | - | 27 | 90 | 50 | 18 | 60.0 | 73.54 | 66.77 | 35.06 | 88.53 | 23.60 | 0.67 | <.001 |
| RAISE-ETP OOCV | ± ΔCDSS 6 months | 0.44 (<.001) | 19.32 | 86 | 139 | 58 | 30 | 74.13 | 50.85 | 62.49 | 59.72 | 66.66 | 26.38 | 0.69 | <.001 |
|  | ± ΔCDSS 12 months | 0.53 (<.001) | 29.06 | 66 | 60 | 56 | 26 | 71.74 | 60.56 | 66.15 | 54.09 | 76.78 | 30.88 | 0.73 | <.001 |
|  | ± PPD | - | - | 17 | 86 | 44 | 28 | 37.77 | 76.72 | 57.25 | 27.87 | 83.81 | 11.68 | 0.69 | <.001 |

Models were trained using biological-only variables, without a feature selection method but otherwise using the same methodology. Biological variables included V1l05, V1l06, V1l07, V1l09, ph05, ph04, ph06 and phbmi. Abbreviations: TP, true positive; TN, true negative; FP, false positive; FN, false negative; sens., sensitivity; spec., specificity; BAC, balanced accuracy; PPV, positive predictive value; NPV, negative predictive value; PSI, prognostic summary index; AUC-ROC, area under the receiver operating characteristic curve*;* EUFEST, European first episode schizophrenia trial; RAISE-ETP, recovery after an initial schizophrenia episode early treatment program; CDSS, Calgary depression scale for schizophrenia; PPD, post-psychotic depression; OOT, out-of-training; OOCV, out-of-cross-validation.

Table S21. Comparison of models’ performance when trained with all the available harmonized variables and when removing the biological variables.

| Outcome label | Sample | Baseline variables, n | Sens. | Spec. | BAC (p label perm.) | Wilcoxon signed-rank statistic (p value, FDR corrected) |
| --- | --- | --- | --- | --- | --- | --- |
| ±ΔCDSS (6 months) | EUFEST OOCV | 123 (all) | 85.71 | 66.98 | 76.35 (<.001) | W = 12397.0 (<.001) |
|  |  | 115 | 87.62 | 63.26 | 75.44 (<.001) |  |
|  | RAISE OOCV | 123 (all) | 68.10 | 64.41 | 66.26 (<.001) | W = 6357.0 (<.001) |
|  |  | 115 | 74.13 | 50.85 | 62.49 (<.001) |  |
| ±ΔCDSS (12 months) | EUFEST OOCV | 123 (all) | 68.48 | 77.63 | 73.05 (0.026) | W = 9193.0 (<.001) |
|  |  | 115 | 60.86 | 81.58 | 71.22 (<.001) |  |
|  | RAISE OOCV | 123 (all) | 70.65 | 66.90 | 68.78 (<.001) | W = 8264.5 (<.001) |
|  |  | 115 | 71.74 | 60.56 | 66.15 (<.001) |  |
| ±PPD | EUFEST OOCV | 123 (all) | 49.37 | 68.46 | 58.92 (<.001) | W = 23251.5 (0.089) |
|  |  | 115 | 53.16 | 68.05 | 60.61 (<.001) |  |
|  | RAISE OOCV | 123 (all) | 40.0 | 78.31 | 59.15 (<.001) | W = 6976.0 (0.232) |
|  |  | 115 | 37.77 | 76.72 | 57.25 (<.001) |  |

Models were trained using all available harmonized variables and also removing the biological variables. Biological variables included V1l05, V1l06, V1l07, V1l09, ph05, ph04, ph06 and phbmi. To test for the differences in predictions using all the harmonized variables and the condensed version, the predicted OOCV labels were tested for significance using the Wilcoxon signed-rank test of the model probability scores. : BAC, balanced accuracy; CDSS, Calgary depression scale for schizophrenia; EUFEST, European first episode schizophrenia trial; FDR, false discovery rate; OOCV, out-of-cross-validation; OOT, out-of-training; PPD, post-psychotic depression; RAISE-ETP, recovery after an initial schizophrenia episode early treatment program; sens., sensitivity; spec., specificity.


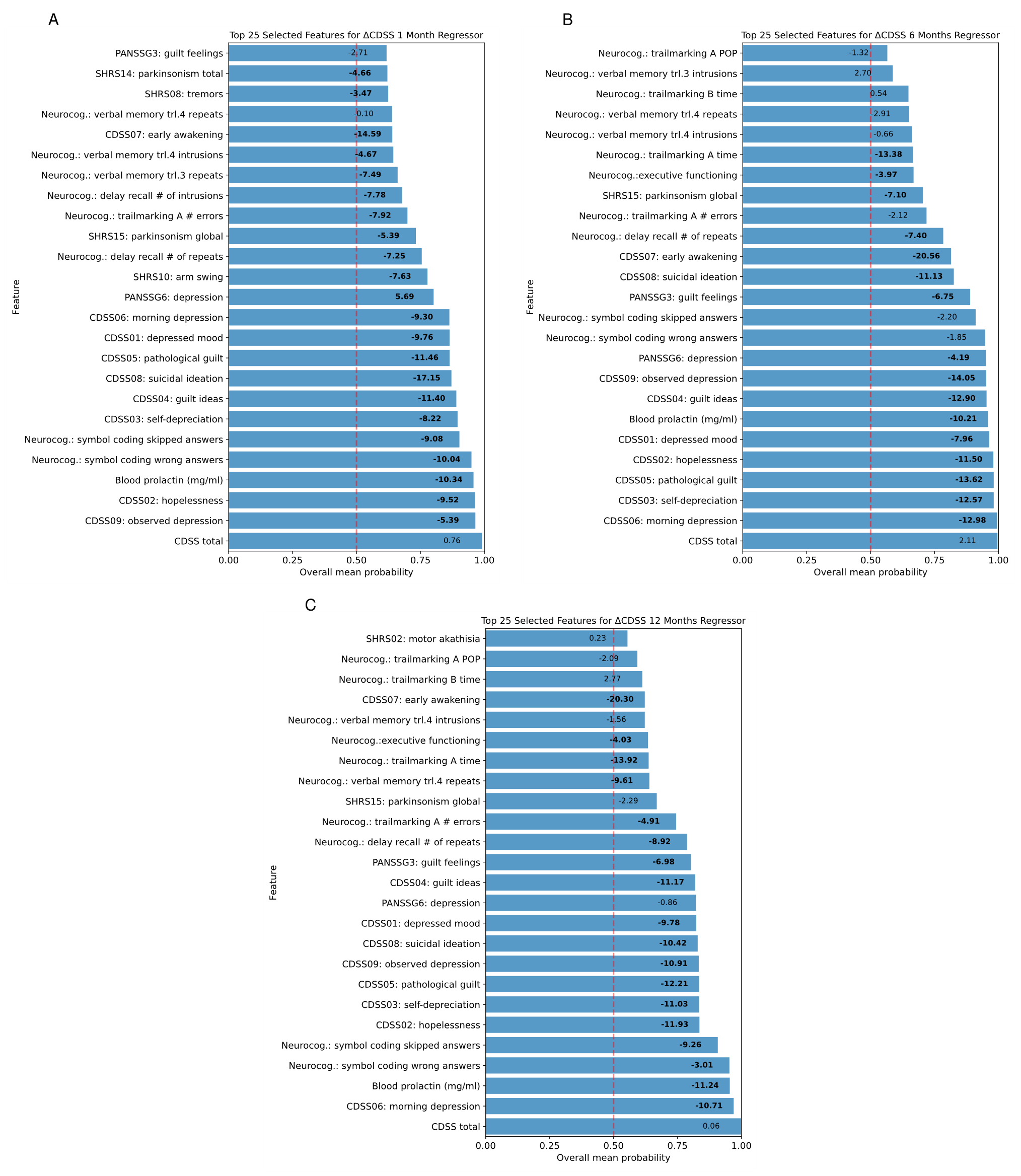


The relevance of features was assessed using two metrics: feature selection probability and overall cross-validation ratio (CVR; see **Supplementary Methods**). We highlight the top 25 predictors based on selection probability for the support vector regressors predicting ΔCDSS at 1 month (A), 6 months (B), and 12 months (C) using all available variables in the EUFEST sample. The values of the variables are color-coded blue if the selection probability ≥ 0.5. The text values represent the CVR of the features, set in bold if |CVR| ≥ 3. These two metrics were used to evaluate the consistency of selection, weight, and sign of the features used by the models. Abbreviations: CDSS, Calgary depression scale for schizophrenia; EUFEST, European first episode schizophrenia trial; Neurocog., neurocognitive assessment; PANSS, positive and negative syndrome scale, where P means a positive score, N means a negative score and G means a general score; SHRS: Scale for the assessment of negative symptoms.

Figure S1. Top 25 predictors of regressors trained with all available variables in the EUFEST sample.


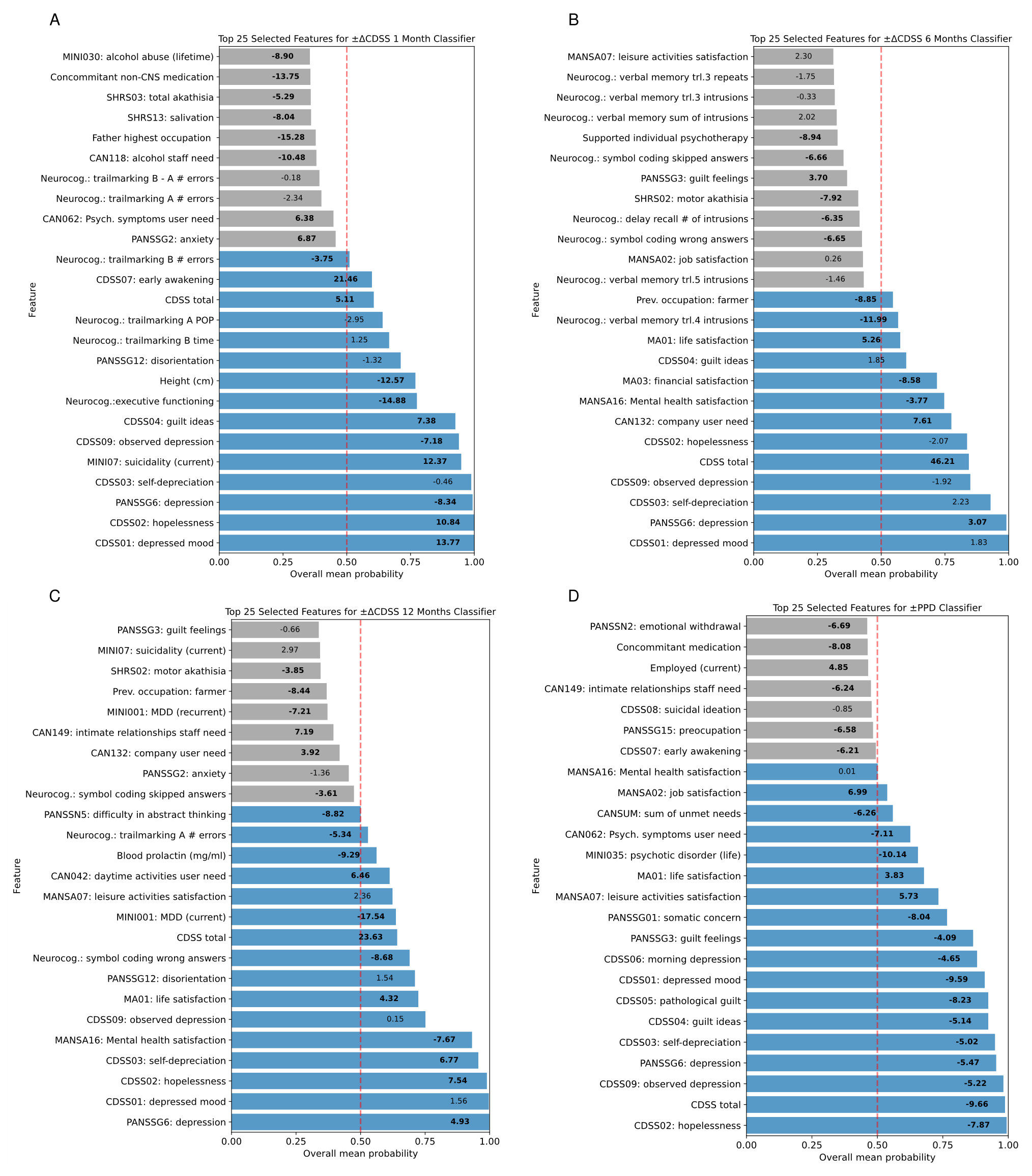


The relevance of features was assessed using two metrics: feature selection probability and overall cross-validation ratio (CVR; see **Supplementary Methods**). We highlight the top 25 predictors based on selection probability for the support vector classifiers predicting ±ΔCDSS at 1 month (A), 6 months (B), 12 months (C) and post-psychotic depression (±PPD; D) using all available variables in the EUFEST sample. The values of the variables are color-coded blue if the selection probability ≥ 0.5. The text values represent the CVR of the features, set in bold if |CVR| ≥ 3. These two metrics were used to evaluate the consistency of selection, weight, and sign of the features used by the models. Abbreviations: CAN, Camberwell assessment of needs; CDSS, Calgary depression scale for schizophrenia; EUFEST, European first episode schizophrenia trial; MANSA, Manchester short assessment of quality of life; MINI, mini international neuropsychiatric interview; Neurocog., neurocognitive assessment; PANSS, positive and negative syndrome scale, where P means a positive score, N means a negative score and G means a general score; SHRS: Scale for the assessment of negative symptoms.

Figure S2. Top 25 predictors of classifiers trained with all available variables in the EUFEST sample.


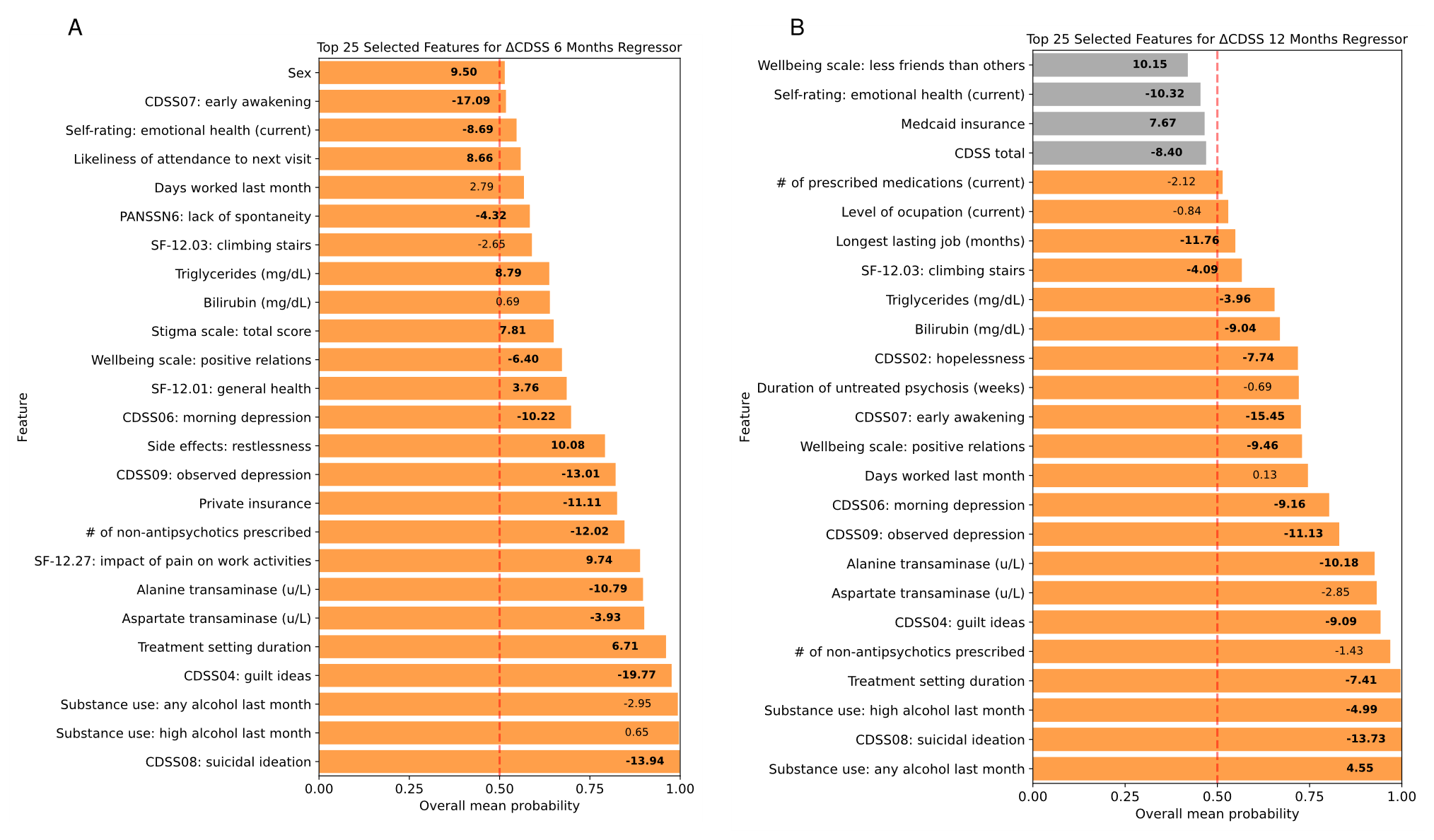


Figure S3. Top 25 predictors of regressors trained with all available variables in the RAISE-ETP sample.

The relevance of features was assessed using two metrics: feature selection probability and overall cross-validation ratio (CVR; see **Supplementary Methods**). We highlight the top 25 predictors based on selection probability for the support vector regressors predicting ΔCDSS at 6 months (A), and 12 months (B) using all available variables in the RAISE-ETP sample. The values of the variables are color-coded orange if the selection probability ≥ 0.5. The text values represent the CVR of the features, set in bold if |CVR| ≥ 3. These two metrics were used to evaluate the consistency of selection, weight, and sign of the features used by the models. Abbreviations: CDSS, Calgary depression scale for schizophrenia; PANSS, positive and negative syndrome scale, where P means a positive score, N means a negative score and G means a general score; RAISE-ETP, recovery after an initial schizophrenia episode early treatment program; SF-12, 12-item short form survey.


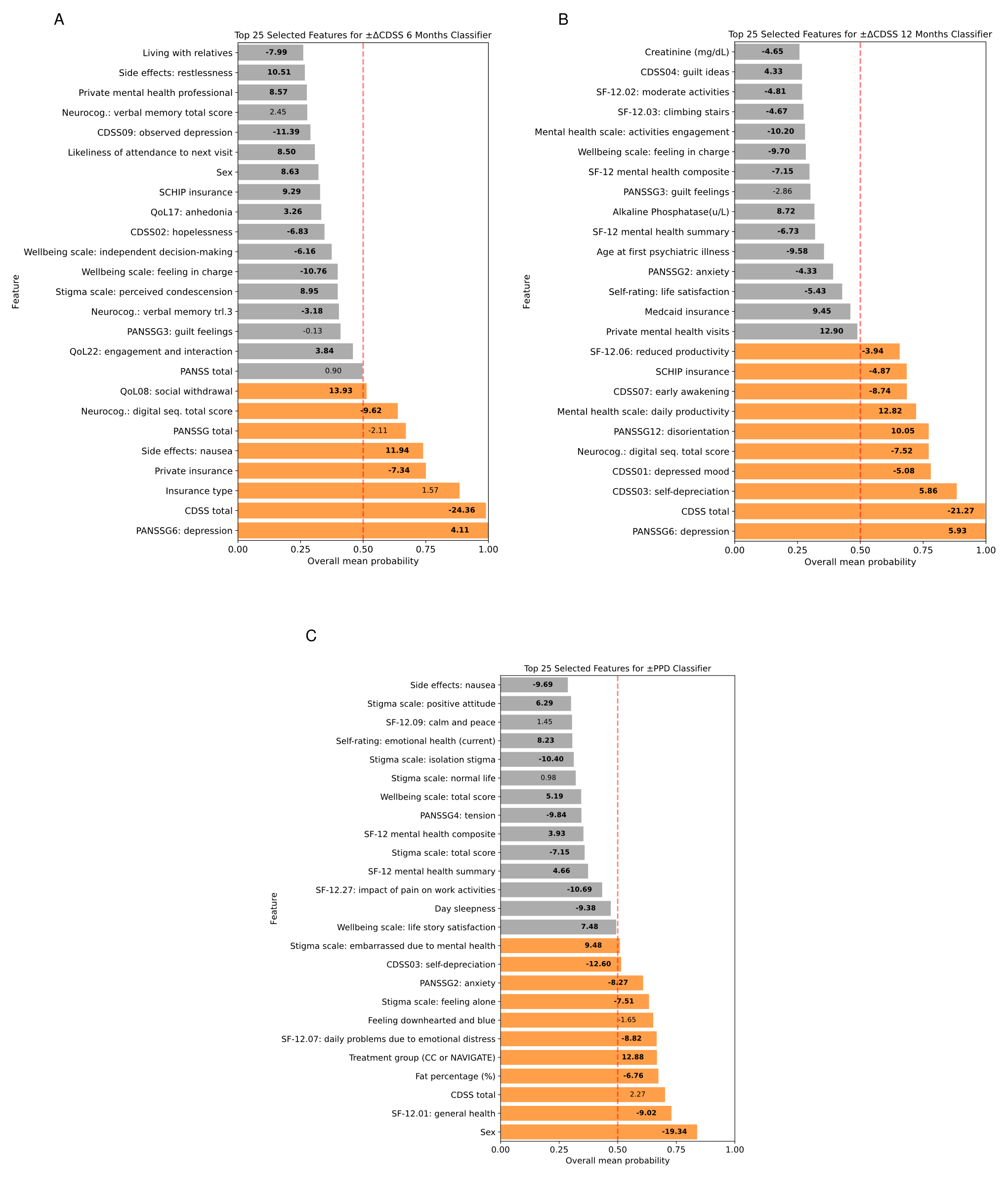


The relevance of features was assessed using two metrics: feature selection probability and overall cross-validation ratio (CVR; see **Supplementary Methods**). We highlight the top 25 predictors based on selection probability for the support vector classifiers predicting ±ΔCDSS at 6 months (A), 12 months (B) and post-psychotic depression (±PPD; C) using all available variables in the RAISE-ETP sample. The values of the variables are color-coded orange if the selection probability ≥ 0.5. The text values represent the CVR of the features, set in bold if |CVR| ≥ 3. These two metrics were used to evaluate the consistency of selection, weight, and sign of the features used by the models. Abbreviations: CC, community-based care; CDSS, Calgary depression scale for schizophrenia; PANSS, positive and negative syndrome scale, where P means a positive score, N means a negative score and G means a general score; QoL, quality of life scale; RAISE-ETP, recovery after an initial schizophrenia episode early treatment program; SCHIP, State children's health insurance program; SF-12, 12-item short form survey.

Figure S4. Top 25 predictors of classifiers trained with all available variables in the RAISE-ETP sample.


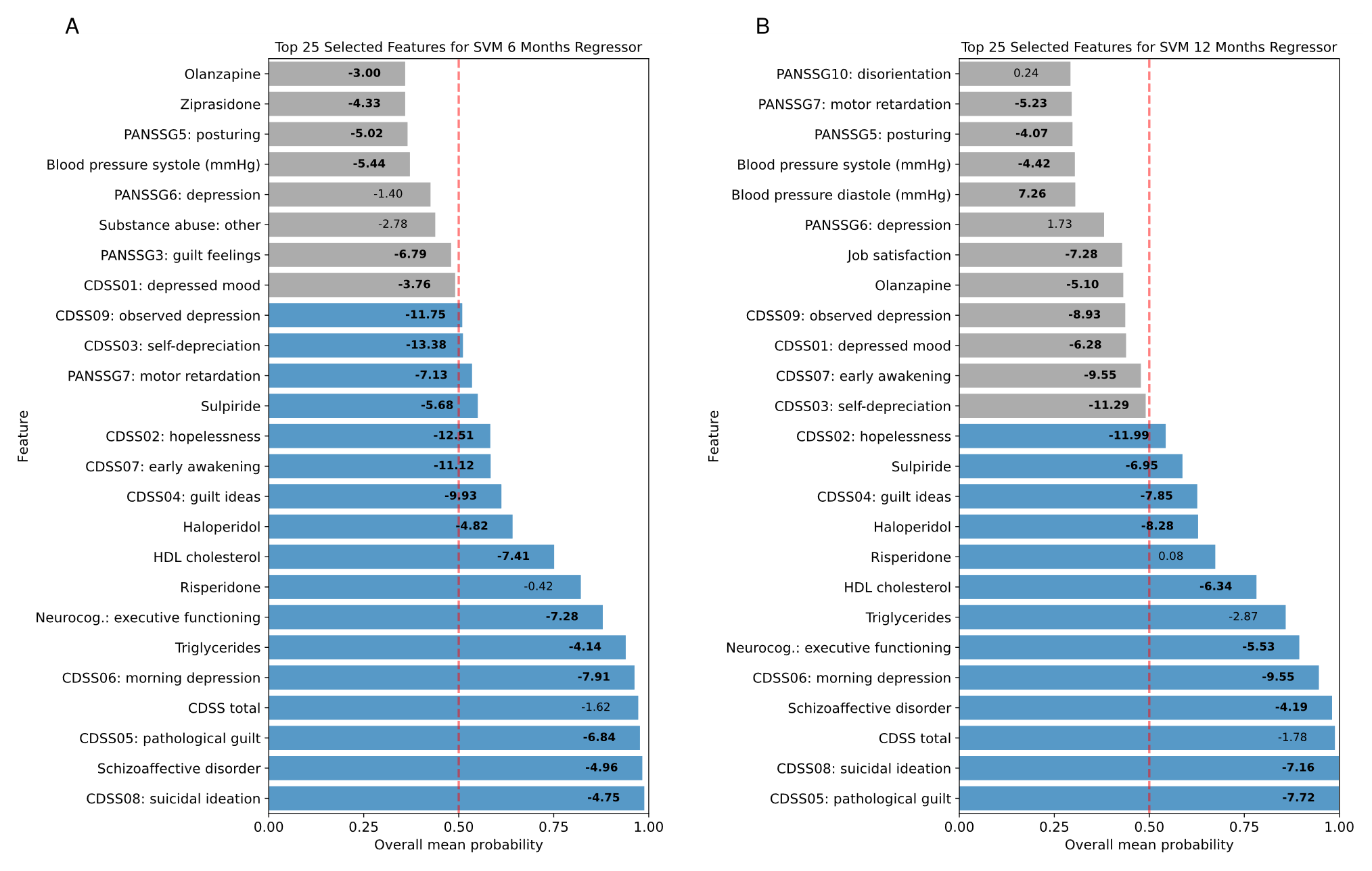


The relevance of features was assessed using two metrics: feature selection probability and overall cross-validation ratio (CVR; see **Supplementary Methods**). We highlight the top 25 predictors based on selection probability for the support vector regressors predicting ΔCDSS at 6 months (A), and 12 months (B) using the harmonized variables and training with the EUFEST sample (see **Supplementary Methods**). The values of the variables are color-coded blue if the selection probability ≥ 0.5. The text values represent the CVR of the features, set in bold if |CVR| ≥ 3. These two metrics were used to evaluate the consistency of selection, weight, and sign of the features used by the models. Abbreviations: CDSS, Calgary depression scale for schizophrenia; EUFEST, European first episode schizophrenia trial; Neurocog., neurocognitive assessment; PANSS, positive and negative syndrome scale, where P means a positive score, N means a negative score and G means a general score; HDL, high-density lipoproteins.

Figure S5. Top 25 predictors of regressors trained with harmonized variables in the EUFEST sample.


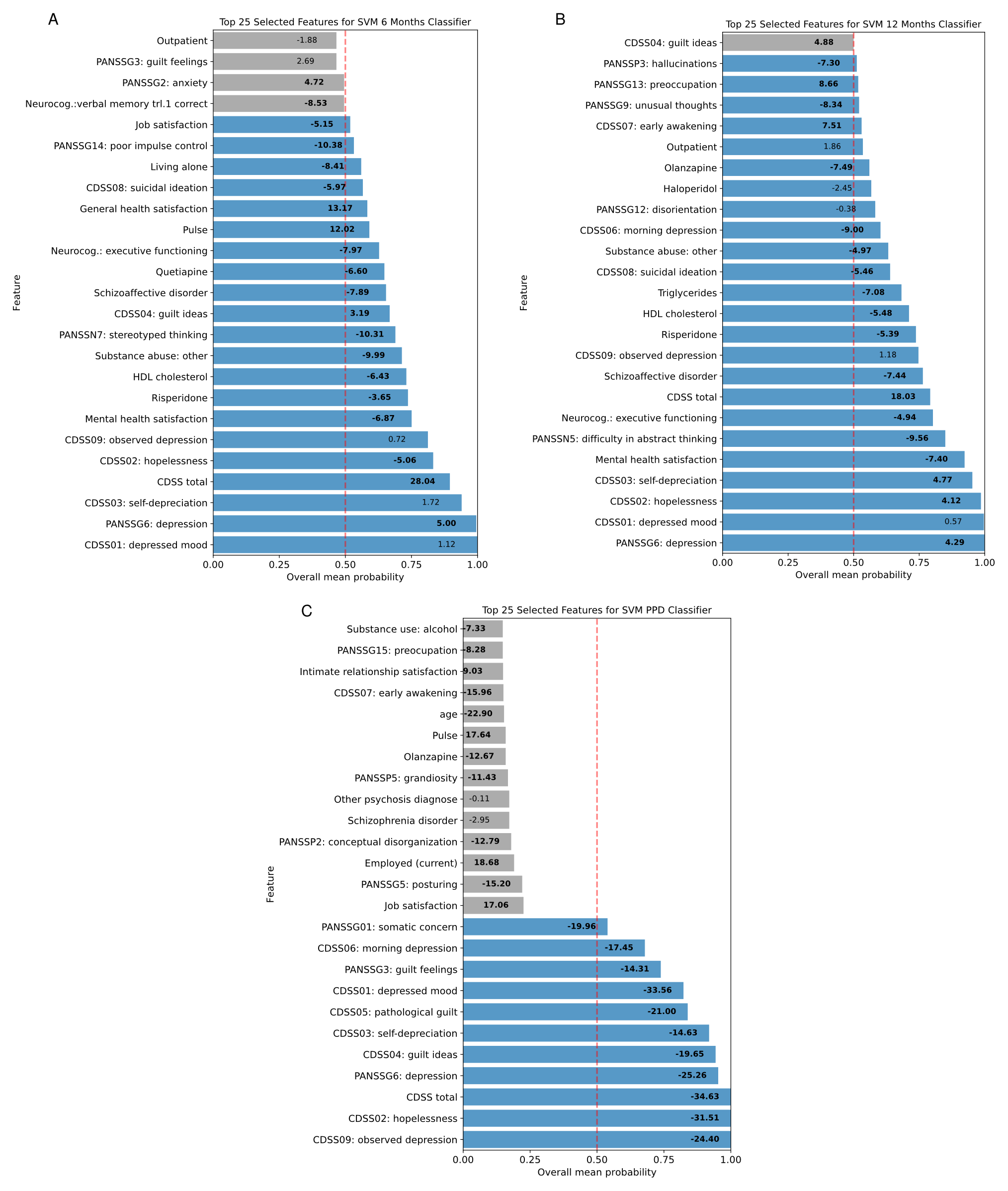


The relevance of features was assessed using two metrics: feature selection probability and overall cross-validation ratio (CVR; see **Supplementary Methods**). We highlight the top 25 predictors based on selection probability for the support vector classifiers predicting ±ΔCDSS at 6 months (A), 12 months (B) and post-psychotic depression (±PPD; C) using the harmonized variables and training with the EUFEST sample (see **Supplementary Methods**). The values of the variables are color-coded blue if the selection probability ≥ 0.5. The text values represent the CVR of the features, set in bold if |CVR| ≥ 3. These two metrics were used to evaluate the consistency of selection, weight, and sign of the features used by the models. Abbreviations: CDSS, Calgary depression scale for schizophrenia; EUFEST, European first episode schizophrenia trial; Neurocog., neurocognitive assessment; PANSS, positive and negative syndrome scale, where P means a positive score, N means a negative score and G means a general score; HDL, high-density lipoproteins.

Figure S6. Top 25 predictors of classifiers trained with harmonized variables in the EUFEST sample.


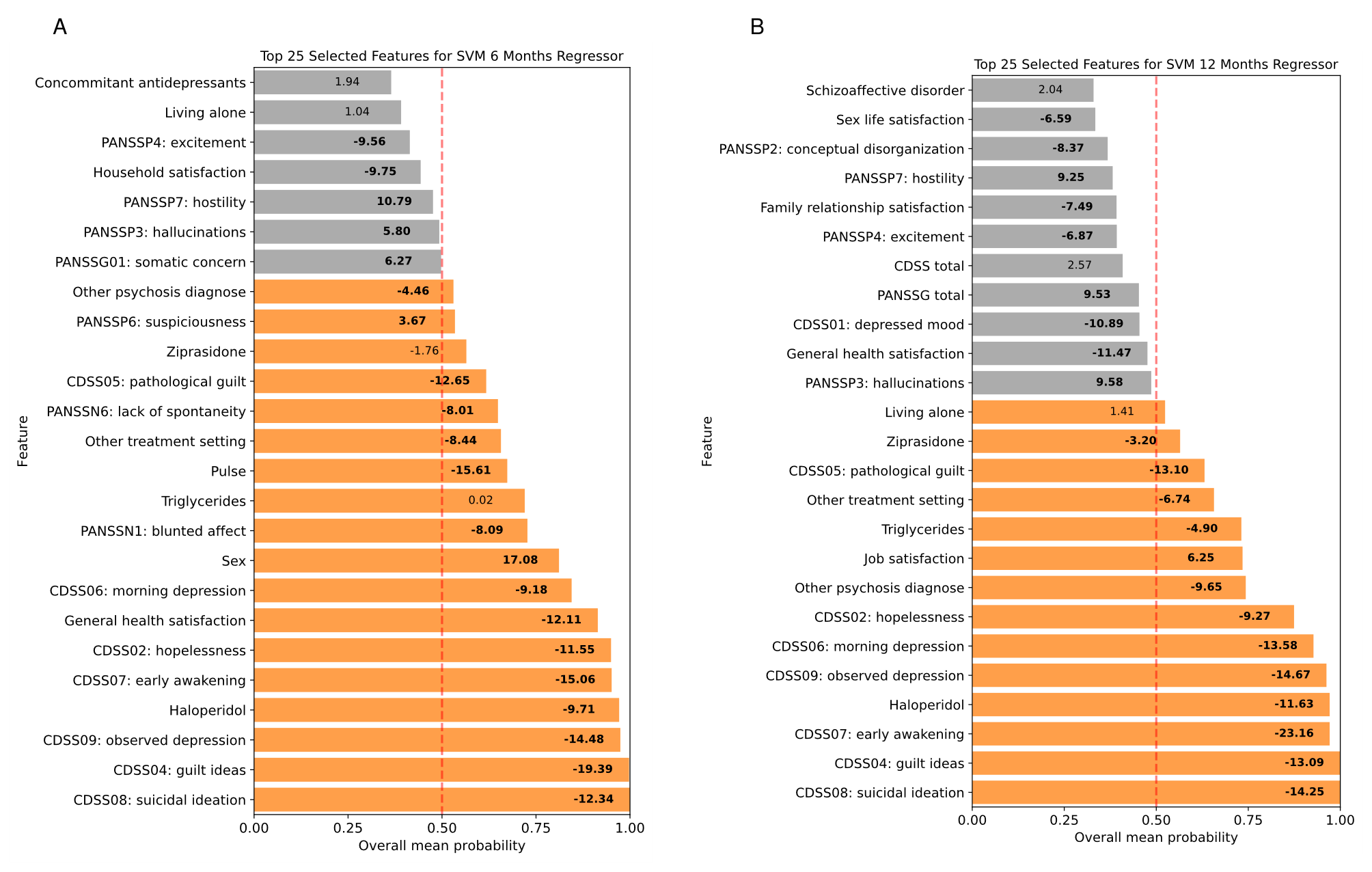


The relevance of features was assessed using two metrics: feature selection probability and overall cross-validation ratio (CVR; see **Supplementary Methods**). We highlight the top 25 predictors based on selection probability for the support vector regressors predicting ΔCDSS at 6 months (A), and 12 months (B) using the harmonized variables and training with the RAISE-ETP sample (see **Supplementary Methods**). The values of the variables are color-coded orange if the selection probability ≥ 0.5. The text values represent the CVR of the features, set in bold if |CVR| ≥ 3. These two metrics were used to evaluate the consistency of selection, weight, and sign of the features used by the models. Abbreviations: CDSS, Calgary depression scale for schizophrenia; PANSS, positive and negative syndrome scale, where P means a positive score, N means a negative score and G means a general score; RAISE-ETP, recovery after an initial schizophrenia episode early treatment program.

Figure S7. Top 25 predictors of regressors trained with harmonized variables in the RAISE-ETP sample.


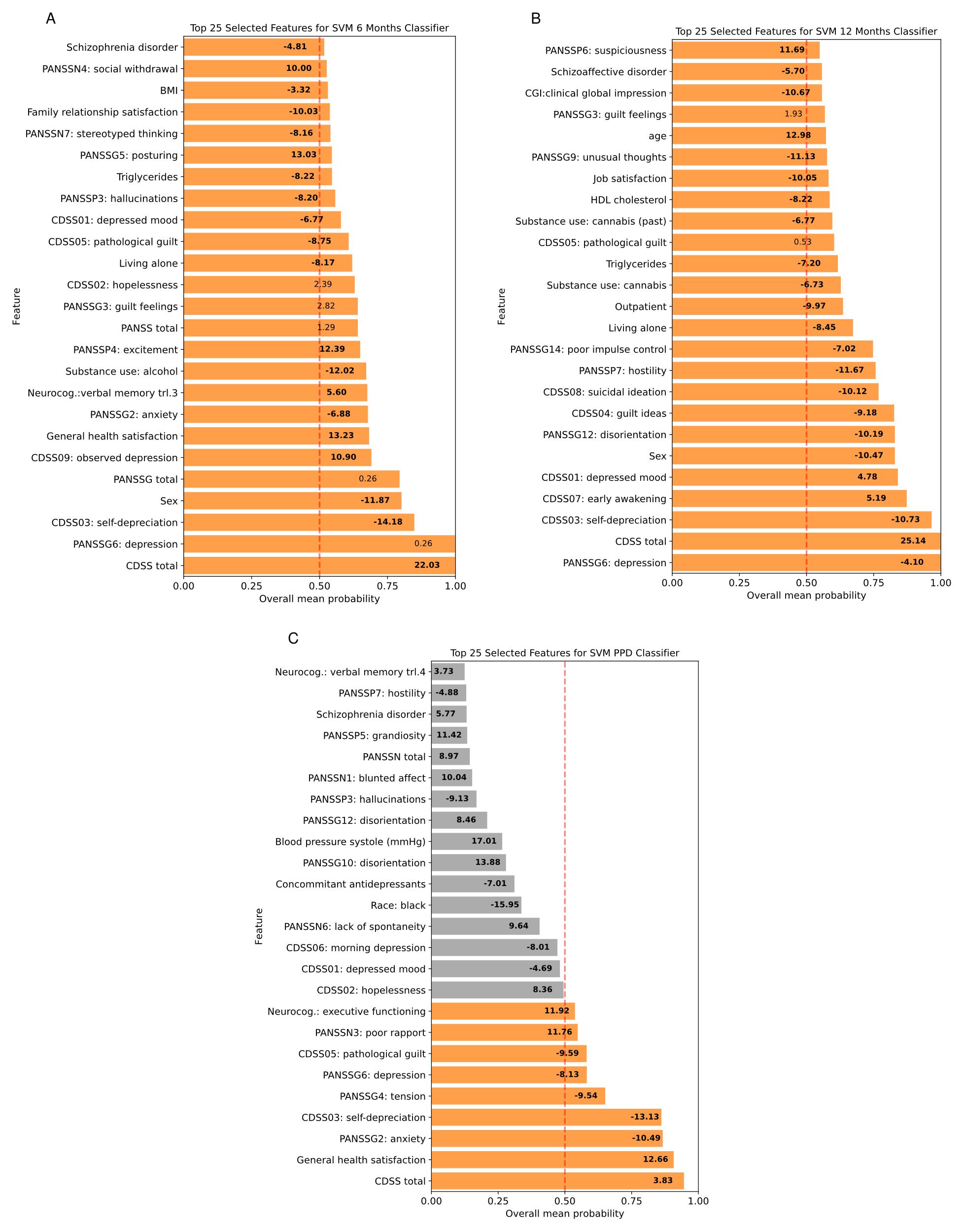


The relevance of features was assessed using two metrics: feature selection probability and overall cross-validation ratio (CVR; see **Supplementary Methods**). We highlight the top 25 predictors based on selection probability for the support vector classifiers predicting ±ΔCDSS at 6 months (A), 12 months (B) and post-psychotic depression (±PPD; C) using the harmonized variables and training with the RAIE-ETP sample (see **Supplementary Methods**). The values of the variables are color-coded orange if the selection probability ≥ 0.5. The text values represent the CVR of the features, set in bold if |CVR| ≥ 3. These two metrics were used to evaluate the consistency of selection, weight, and sign of the features used by the models. Abbreviations: BMI, body mass index; CDSS, Calgary depression scale for schizophrenia; CGI, clinical global impression; HDL, high-density lipoproteins; Neurocog., Neurocognitive assessment; PANSS, positive and negative syndrome scale, where P means a positive score, N means a negative score and G means a general score; RAISE-ETP, recovery after an initial schizophrenia episode early treatment program.

Figure S8. Top 25 predictors of classifiers trained with harmonized variables in the RAISE-ETP sample.


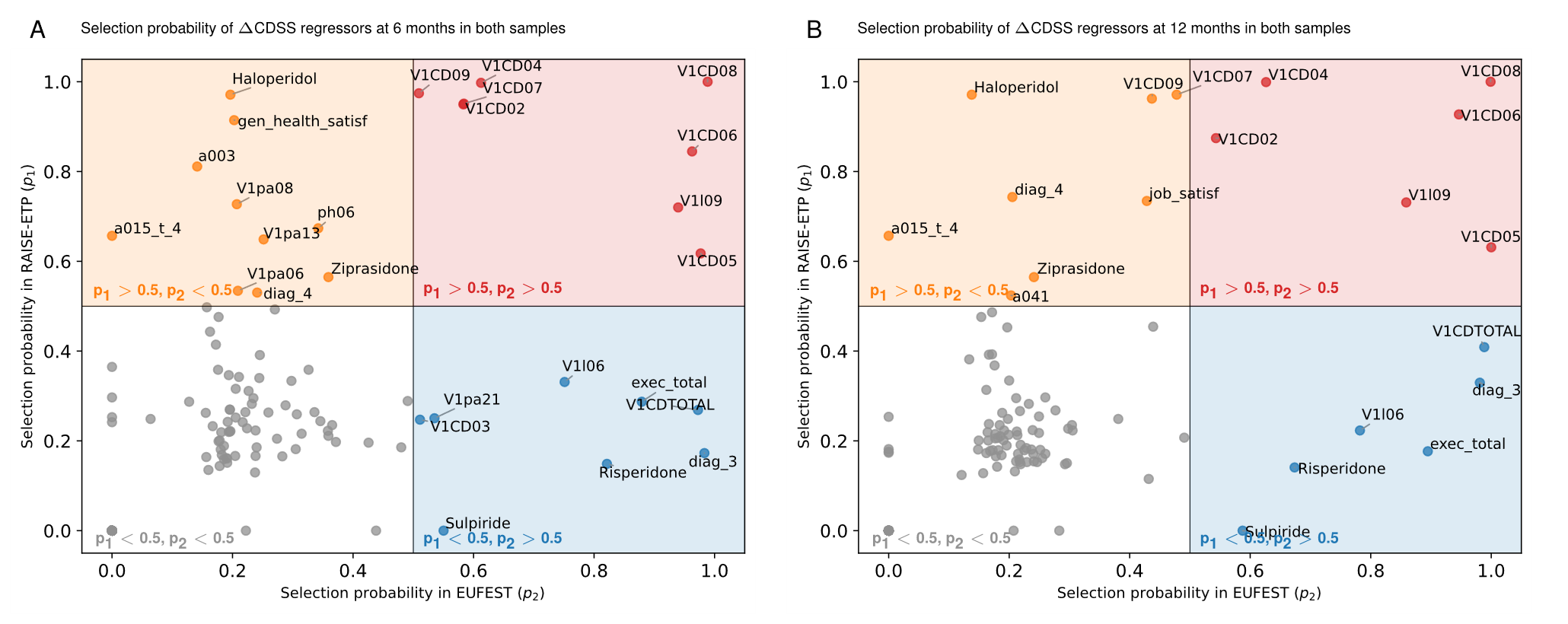


Figure S9. Selection probability of features across ΔCDSS regressors in both samples.

The robustness of the selected features when regressors are trained using the two independent samples was assessed by visualizing the selection probability of each harmonized feature when the model was trained with either cohort for the ΔCDSS label at 6 months (A) and at 12 months (B). Variable codes definitions can be seen in **Table S4**. Abbreviations: CDSS, Calgary depression scale for schizophrenia; EUFEST, European first episode schizophrenia trial; RAISE-ETP, recovery after an initial schizophrenia episode early treatment program.

**
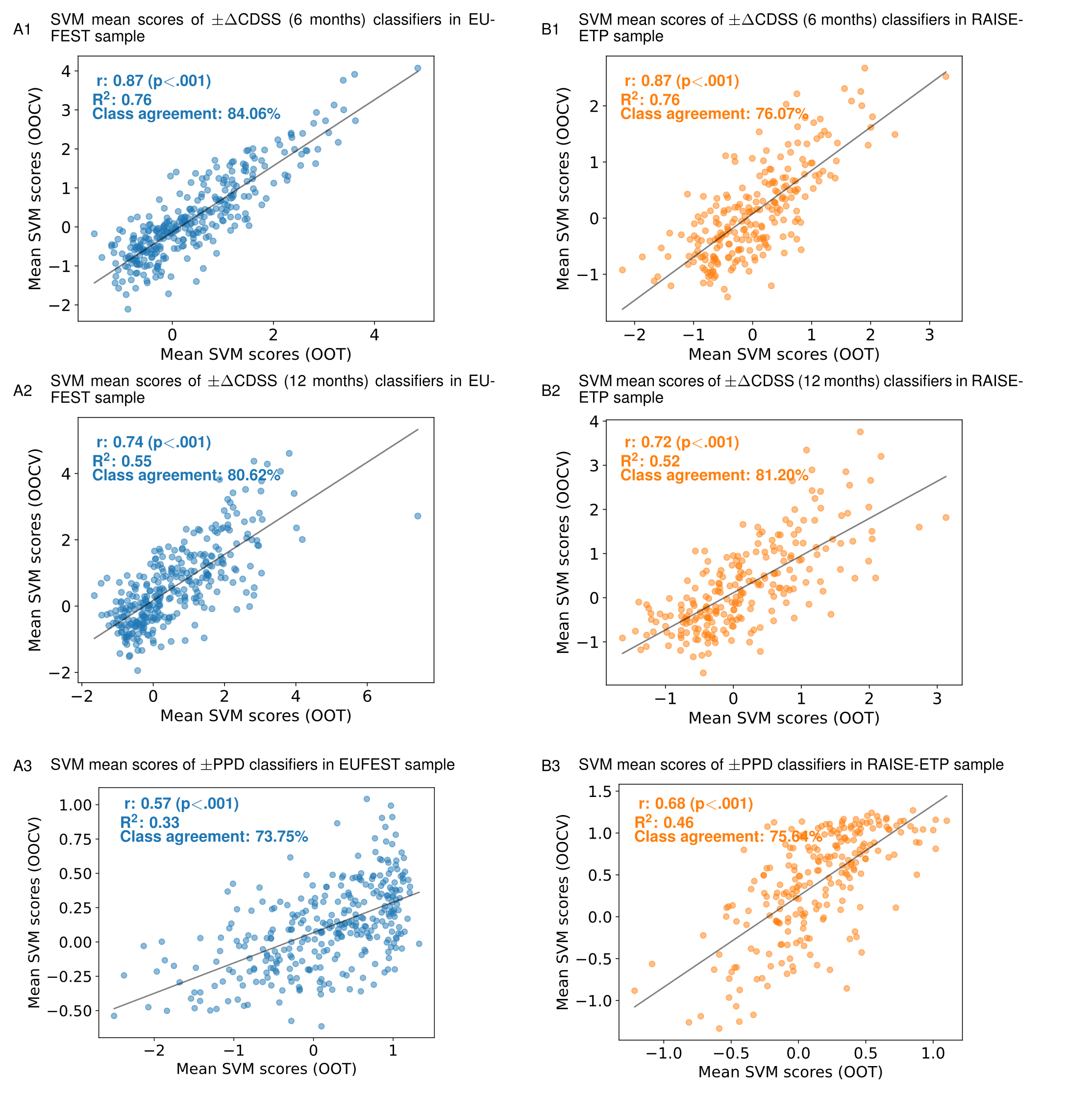
**

Figure S10. Correlation between SVM out-of-training and out-of-cross-validation scores for EUFEST and RAISE-ETP patients.

The agreement and reliability of model predictions for the same patients were assessed by comparing predictions made using out-of-training (OOT) data and out-of-cross-validation (OOCV) data, which represents an external independent sample. This analysis illustrated the decision scores for the ±ΔCDSS classifiers at 6 months (A1 for the EUFEST sample and B1 for the RAISE-ETP sample) and 12 months (A2 for the EUFEST sample and B2 for the RAISE-ETP sample), as well as for the ±PPD classifier (A2 for the EUFEST sample and B2 for the RAISE-ETP sample). Pearson’s correlation coefficient, the coefficient of determination (R²), and the predicted outcome agreement were also calculated and are presented in the graphs. Abbreviations: CDSS, Calgary depression scale for schizophrenia; EUFEST, European first episode schizophrenia trial; RAISE-ETP, recovery after an initial schizophrenia episode early treatment program.


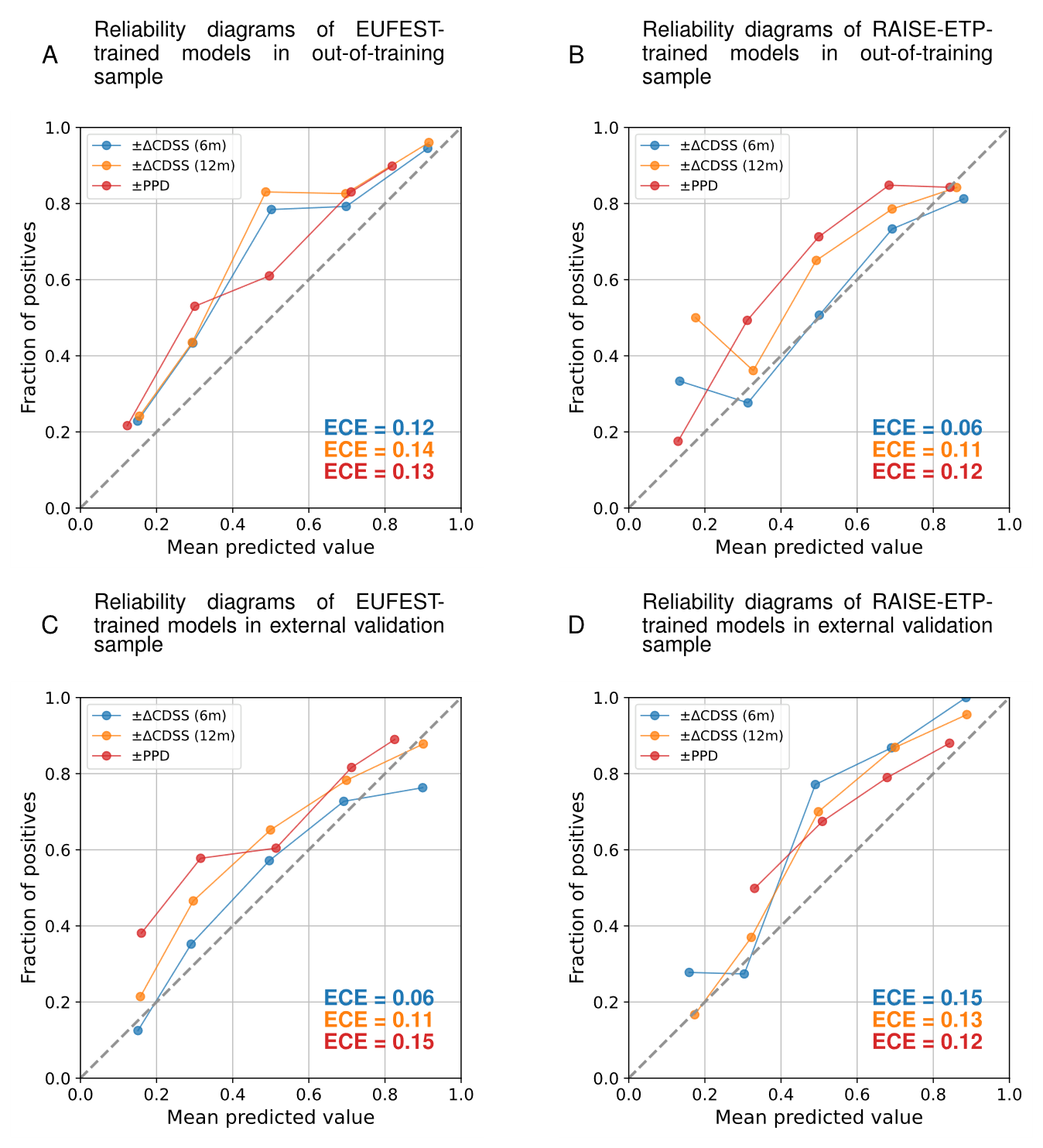


Calibration analyses were performed using reliability maps and expected calibration error (ECE; see **Supplementary Methods**). The calibration was repeated at the out-of-training (OOT; A and B) and out-of-cross-validation level (OOCV; C and D), for models trained using the EUFEST (A and C) and RAISE-ETP samples (B and D). The ECE, which ranges from 0 to 1 with 0 indicating perfect calibration, was calculated for each sample and classifier and is shown in the graphs. This way, (A) shows the calibration for EUFEST-trained models in the OOT sample (EUFEST patients). (B) shows the calibration for EUFEST-trained models in the OOCV sample (RAISE-ETP patients). (C) shows the calibration for RAISE-ETP-trained models in the OOT sample (RAISE-ETP patients). (D) shows the calibration for RAISE-ETP-trained models in the OOCV sample (EUFEST patients). Note that positives denote a decrease in depressive symptoms or no depressive episodes. Abbreviations: CDSS, Calgary depression scale for schizophrenia; EUFEST, European first episode schizophrenia trial; PPD, post-psychotic depression; RAISE-ETP, recovery after an initial schizophrenia episode early treatment program.

Figure S11. Reliability diagrams of the classifiers.


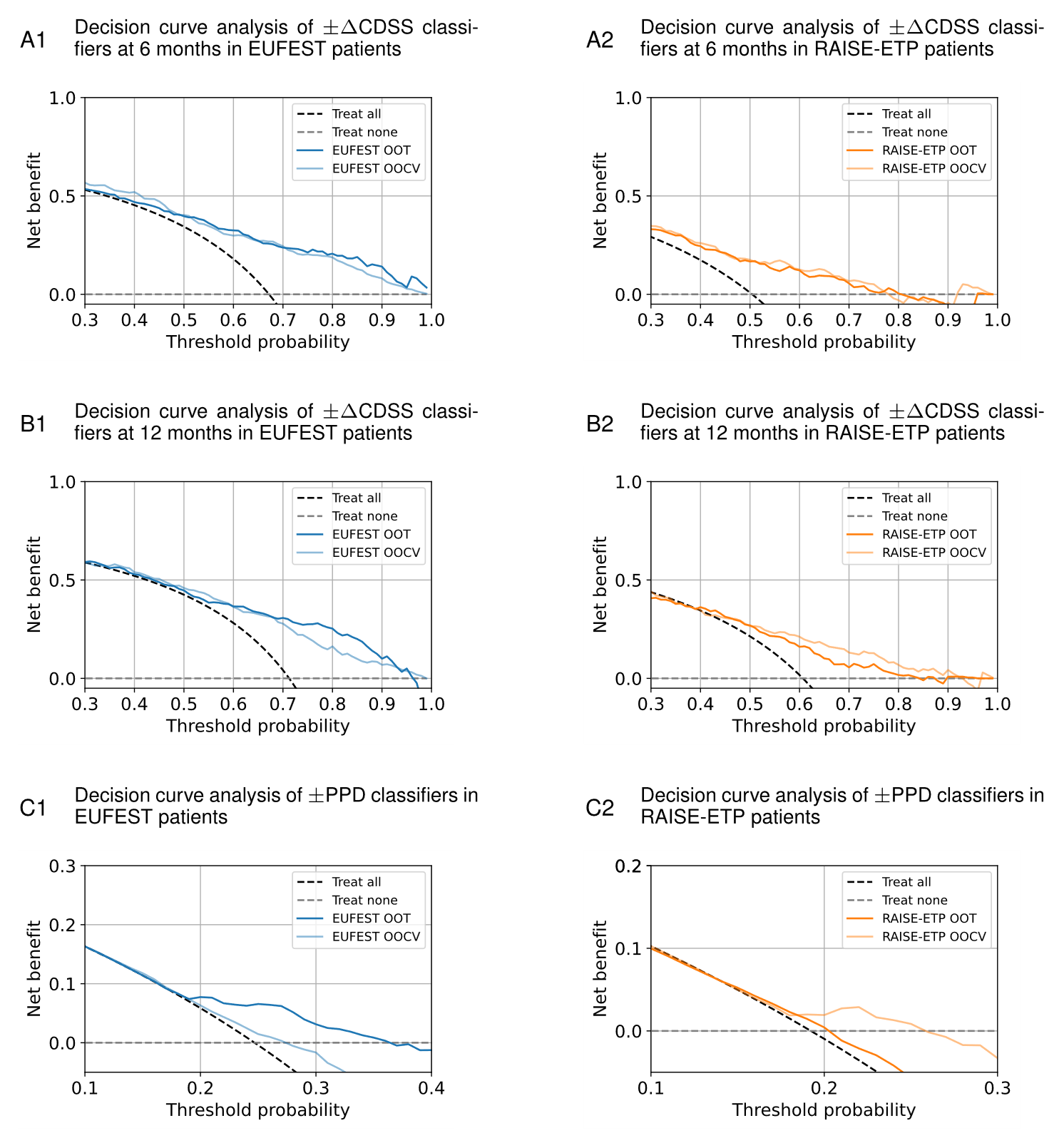


Decision curves were calculated for classifiers predicting ±ΔCDSS at 6 months (A1 and A2 for net benefit in EUFEST and RAISE-ETP patients, respectively), at 12 months (B1 and B2 for net benefit in EUFEST and RAISE-ETP patients, respectively), and for the post-psychotic depression label (±PPD; C1 and C2 for net benefit in EUFEST and RAISE-ETP patients, respectively). Curves are shown for models evaluated both out-of-training (OOT) and out-of-cross-validation (OOCV). Specifically, (A1, B1, C1) illustrate the net benefit of models applied to EUFEST patients, trained on the EUFEST dataset (EUFEST OOT) and RAISE-ETP dataset (EUFEST OOCV). Conversely, (A2, B2, C2) display the net benefit for RAISE-ETP patients, with models trained on the RAISE-ETP dataset (RAISE-ETP OOT) and EUFEST dataset (RAISE-ETP OOCV). The "treat all" trace shows the net benefit of treating every FEP patient (e.g., with antidepressant or a talking therapy), while the "treat none" trace shows the net benefit of treating no FEP patients. Abbreviations: CDSS, Calgary depression scale for schizophrenia; EUFEST, European first episode schizophrenia trial; RAISE-ETP, recovery after an initial schizophrenia episode early treatment program.

Figure S12. Decision curves of classifiers.


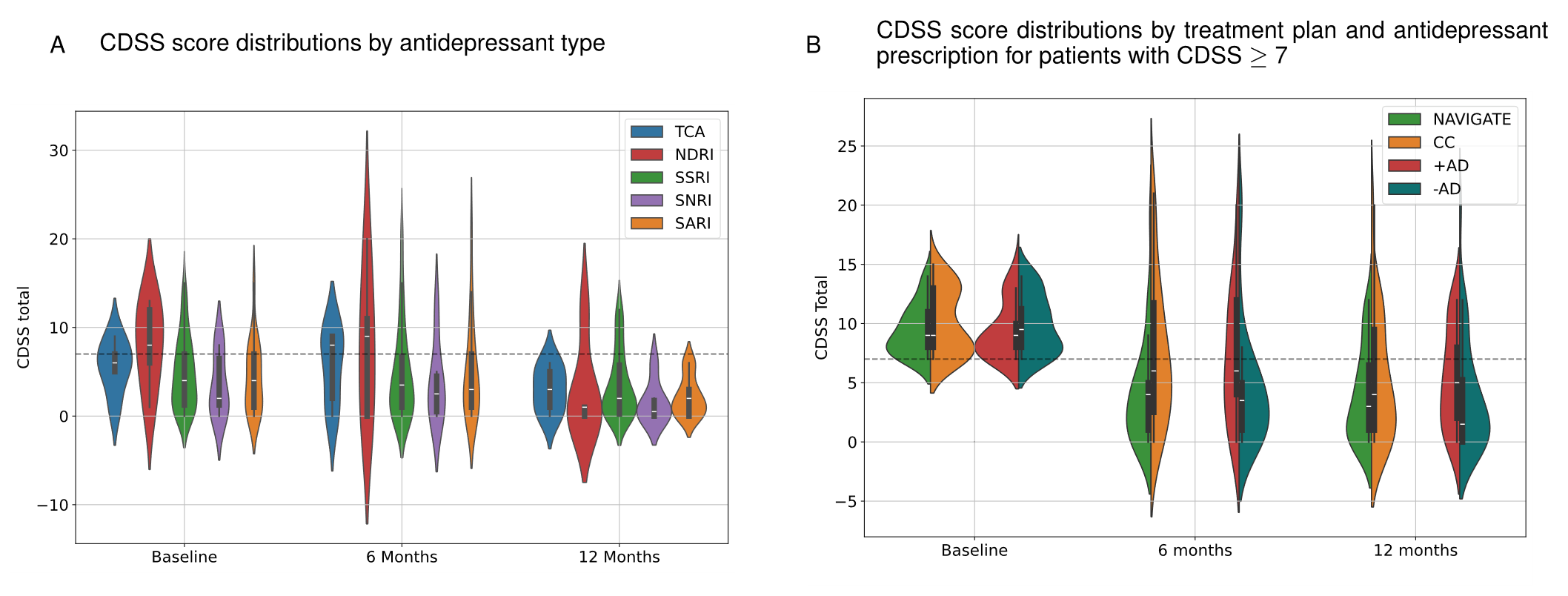


Figure S13. Calgary depression scores by treatment type in the RAISE-ETP sample.

Violin plots with the CDSS total score distributions by antidepressant type (A) and treatment plan and antidepressant prescription for patients with CDSS ≥ 7 (B) at baseline, 6 months, and 12 months. The horizontal dashed line at 7 shows the CDSS threshold to classify patients with a major depressive episode. Abbreviations: Abbreviations: CDSS, Calgary depression scale for schizophrenia; RAISE-ETP, recovery after an initial schizophrenia episode early treatment program.


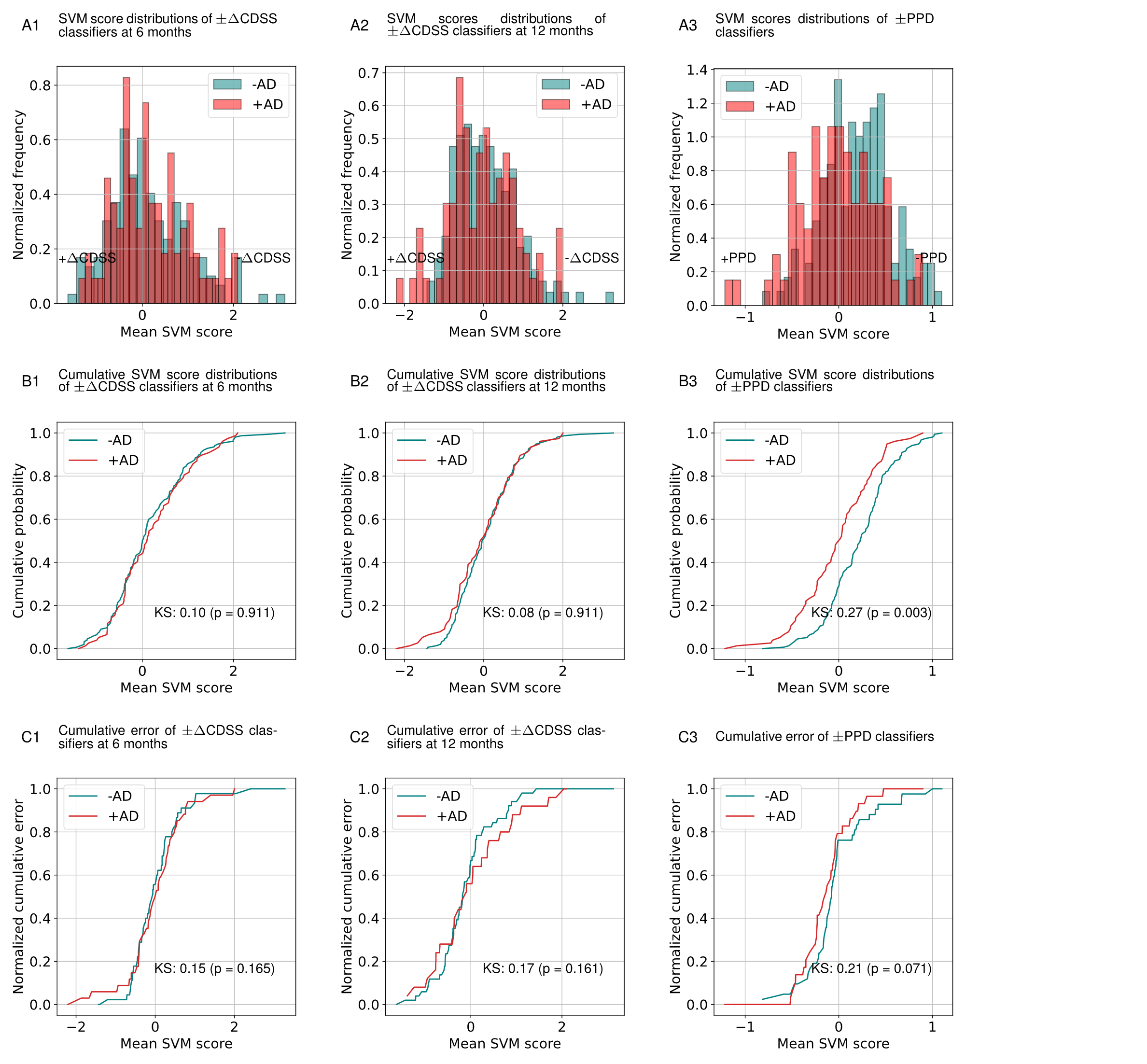


The SVM decision score distributions, cumulative distributions, and cumulative misclassifications of RAISE-ETP patients with and without an antidepressant prescription were visualized for RAISE-ETP-trained classifiers predicting ±ΔCDSS at 6 months (A1, B1, and C1), at 12 months (A2, B2, and C2), and post-psychotic depression (±PPD; A3, B3, and C3). Additionally, to assess the influence of antidepressant prescription on the types of misclassifications, we calculated the normalized cumulative number of misclassifications over patients ranked by their SVM decision scores for both treatment groups. The Kolmogorov-Smirnov test was used to statistically assess differences between the decision score distributions and the normalized cumulative error distributions. Abbreviations: AD, antidepressant; CDSS, Calgary depression scale for schizophrenia; KS, Kolmogorov-Smirnov statistic; RAISE-ETP, recovery after an initial schizophrenia episode early treatment program.

Figure S14. Decision score distributions and cumulative error distributions by antidepressant use in RAISE-ETP patients.


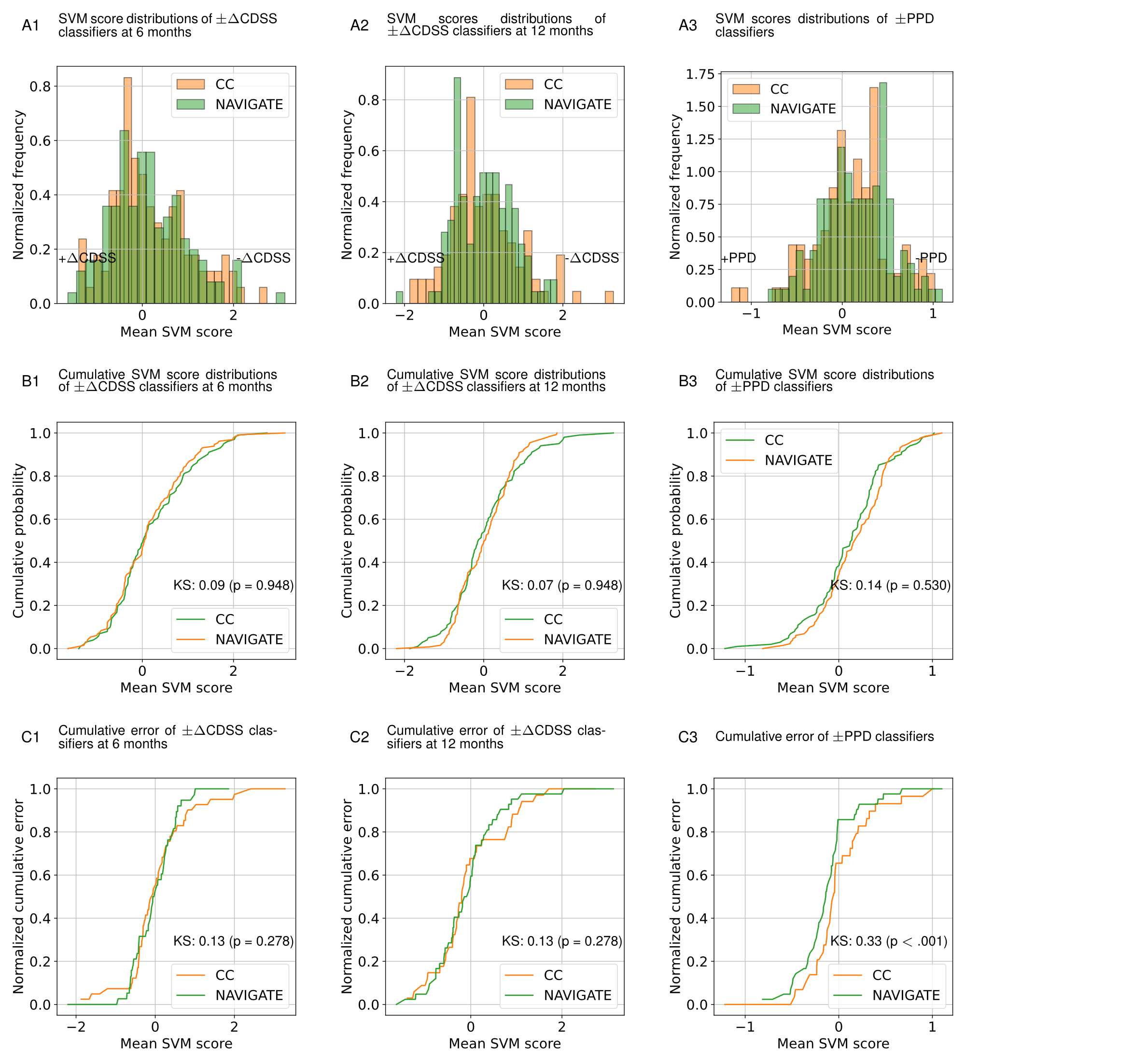


The SVM decision score distributions, cumulative distributions, and cumulative misclassifications of RAISE-ETP patients in the community-based care and NAVIGATE care were visualized for RAISE-ETP-trained classifiers predicting ±ΔCDSS at 6 months (A1, B1, and C1), at 12 months (A2, B2, and C2), and post-psychotic depression (±PPD; A3, B3, and C3). Additionally, to assess the influence of treatment program on the types of misclassifications, we calculated the normalized cumulative number of misclassifications over patients ranked by their SVM decision scores for both treatment groups. The Kolmogorov-Smirnov test was used to statistically assess differences between the decision score distributions and the normalized cumulative error distributions. Abbreviations: CC, community care; CDSS, Calgary depression scale for schizophrenia; KS, Kolmogorov-Smirnov statistic; RAISE-ETP, recovery after an initial schizophrenia episode early treatment program.

Figure S15. Decision score distributions and cumulative error distributions by treatment plan in RAISE-ETP patients.
