# Supplementary material for: AI-based prediction of depression symptomatology in first-episode psychosis patients: insights from the EUFEST and RAISE-ETP clinical trials
Source: Psychol Med. 2025 Jul 30;55:e221. doi: 10.1017/S0033291725100950 (PMC12341035; doi:10.1017/S0033291725100950)
Supplement: Mena et al. supplementary material 2 — Mena et al. supplementary material [file S0033291725100950sup002.zip › supporting files/baseline_extraction_eufest.html]

baseline\_extraction


In [10]:

```
import pandas as pd
import numpy as np

pd.set_option('display.max_rows', 5)
pd.set_option('display.max_columns', None)

#Folder name with original input data.
dir_input = 'Raw data/'

#Folder name for output data.
dir_output = 'Baseline/'
```

In [11]:

```
################################################################################
#Data Extraction
################################################################################
# Sergio Mena Ortega, 2023


#------------- Camberwell Assessment of Needs (CAN) -----------------------------
# OUTPUT dataframe: df_cam

df_cam = pd.read_excel(dir_input+'camber_070727.xlsx', sheet_name = 'Data').filter(regex='^(V1|crfnr)')

#Replacing "9" with NaN - > unknown.
df_cam.replace(9, np.nan, inplace=True)

#Remove non-applicable columns.
df_cam = df_cam.drop(columns = ['V1CA021', 'V1CA163'])

#Calculate sum of no/low need, moderate need and high need from relevant columns. 
df_cam_var_labels = pd.read_excel(dir_input+'camber_070727.xlsx', sheet_name = 'Variable labels', header = None)
df_cam_var_labels = df_cam_var_labels[df_cam_var_labels[0].str.startswith('V1')]
filtered_codes = df_cam_var_labels[df_cam_var_labels[1] == "{0.0: 'No', 1.0: 'No/Moderate', 2.0: 'Unmet need', 9.0: 'not known'}"][0].values
#Add SUM columns
df_cam['V1CASUM_NO'] = (df_cam[filtered_codes] == 0).sum(axis=1)
df_cam['V1CASUM_MODERATE'] = (df_cam[filtered_codes] == 1).sum(axis=1)
df_cam['V1CASUM_UNMET'] = (df_cam[filtered_codes] == 2).sum(axis=1)
# Set sums to NaN if all values in the corresponding rows are NaN.
nan_rows = df_cam[filtered_codes].isna().all(axis=1)
df_cam.loc[nan_rows, ['V1CASUM_NO', 'V1CASUM_MODERATE', 'V1CASUM_UNMET']] = np.nan


#------------- Calgary Depression scale for Schizophrenia ----------------
# OUTPUT dataframe: df_cdss

#Replacing "9" with NaN - > unknown.
df_cdss = pd.read_excel(dir_input+'cdss_070904.xlsx', sheet_name = 'Data').filter(regex='^(V1|crfnr)').replace(9, np.nan)

#Add a total score variable.
df_cdss['V1CDTOTAL'] = df_cdss[['V1CD01', 'V1CD02', 'V1CD03', 'V1CD04', 'V1CD05', 'V1CD06', 'V1CD07', 'V1CD08', 'V1CD09']].sum(axis = 1)

#Assign nan when all values are nan. 
df_cdss.loc[df_cdss[['V1CD01', 'V1CD02', 'V1CD03', 'V1CD04', 'V1CD05', 'V1CD06', 'V1CD07', 'V1CD08', 'V1CD09']].isna().all(axis = 1), 'V1CDTOTAL'] = np.nan


#------------- Clinical global impression.--------------------------------
# OUTPUT dataframe: df_cgi
# - Replacement of 0's (not assessed) with NaN.
df_cgi = pd.read_excel(dir_input+'cgi_070727.xlsx', sheet_name = 'Data').filter(regex='^(V1|crfnr)').replace(0, np.nan)


#------------- Concominant medication -------------------------------------------
# OUTPUT dataframe: df_concommed
#Extract type of drug based on ATC Code (https://www.whocc.no/atc_ddd_index/?code=N&showdescription=no)
def categorize_drug(atcode):
    if type(atcode)  == str: 
        if atcode[:3] == 'N01':
            return 'CONCOMMED_ANESTHETICS'
        elif atcode[:3] == 'N02':
            return 'CONCOMMED_ANALGESICS'
        elif atcode[:3] == 'N03':
            return 'CONCOMMED_ANTIEPILEPTICS'
        elif atcode[:3] == 'N04':
            return 'CONCOMMED_ANTI-PARKINSON DRUGS'
        elif atcode[:3] == 'N05':
            return 'CONCOMMED_PSYCHOLEPTICS'
        elif atcode[:3] == 'N06':
            return 'CONCOMMED_PSYCHOANALEPTICS'
        elif atcode[:3] == 'N07':
            return 'CONCOMMED_OTHER_NSD'
        else:
            return 'CONCOMMED_OTHER_NON-NSD'
    else:
        return None

df_concommed = pd.read_excel(dir_input+'Concommed_070727.xlsx', sheet_name = 'Data', usecols = ['crfnr','dStart1', 'dEind1', 'atcode1'])
#Extract dates of baseline visit. 
df_v1_date = pd.read_excel(dir_input+'v1_070727.xlsx', sheet_name = 'Data').filter(regex='^(rr001|crfnr)')
#Left merge. 
df_concommed  = pd.merge(df_concommed, df_v1_date, on='crfnr', how='left')
# Create "concomminant before" variable, when date of V1 is after start of first concominant prescription.
df_concommed['CONCOMMED_BEFORE'] = (df_concommed['rr001_dat'] >= df_concommed['dStart1']).astype(int) 
# Set CONCOMMED_BEFORE to NaN where R1_pi02_dat is NaN or NaT.
df_concommed.loc[df_concommed['dStart1'].isna(), 'CONCOMMED_BEFORE'] = np.nan
# Store patient ID and CONCOMMED_BEFORE.
df_concommed = df_concommed[['crfnr','CONCOMMED_BEFORE']]

# Define the list of drug prescriptions (it goes up to 25)
medications = [str(i) for i in range(1, 25)]

list_of_classes = ['CONCOMMED_ANESTHETICS', 'CONCOMMED_ANALGESICS', 'CONCOMMED_ANTIEPILEPTICS', 
                   'CONCOMMED_ANTI-PARKINSON DRUGS', 'CONCOMMED_PSYCHOLEPTICS', 
                   'CONCOMMED_PSYCHOANALEPTICS', 'CONCOMMED_OTHER_NSD', 'CONCOMMED_OTHER_NON-NSD']

list_of_TYPE = []

for medication in medications:
    # Read intervention data
    cols = ['crfnr','dStart'+medication, 'dEind'+medication, 'atcode'+medication]
    df_concommed_each = pd.read_excel(dir_input+'Concommed_070727.xlsx', sheet_name='Data', usecols = cols)

    # Left merge
    df_concommed_each = pd.merge(df_concommed_each, df_v1_date, on='crfnr', how='left')

    # Create "concommitant medication before" variable.
    df_concommed_each['CONCOMMED_BEFORE'] = (df_concommed_each['rr001_dat'] >= df_concommed_each['dStart'+medication]).astype(int)
    
    # Mapping of codes to type of drug. 
    for class_drug in list_of_classes:   
        df_concommed_each[class_drug] = df_concommed_each['atcode'+medication].apply(lambda x: 1 if categorize_drug(x) == class_drug else 0) 

    
    # Set dummy codes for types of drugs to 0 if CONCOMMED_BEFORE is 0.
    df_concommed_each.loc[df_concommed_each['CONCOMMED_BEFORE'] == 0, list_of_classes] = 0
    # Append it to the list. 
    list_of_TYPE.append(df_concommed_each[list_of_classes])

    
#Record type of concommitant medications and overlap them. If one type is prescribed more than once, it is stil set to 1. 
df_concommed.loc[:, list_of_classes] = np.where(np.sum(list_of_TYPE, axis=0)>= 1, 1, 0)

# If INTERV_BEFORE is n.nan, then all interventions are nan (since it is not clear if v1 was before or after).
df_concommed.loc[df_concommed['CONCOMMED_BEFORE'].isna(), list_of_classes] = np.nan

#IMPORTANT NEW ADDITION: If there is no record of concomitant medication, then set CONCOMMED_BEFORE to 0 as well as the classes variables.
df_v1 = pd.read_excel(dir_input+'v1_070727.xlsx', sheet_name = 'Data').filter(regex='^(crfnr)')
# Get entries of patients not in df_concommed and set all variables to 0.
df_no_concommed = df_v1[~df_v1['crfnr'].isin(df_concommed['crfnr'])].copy()
df_no_concommed[['CONCOMMED_BEFORE', list_of_classes]] = 0
# Concatenate entries in the register and not in the register.
df_concommed = pd.concat([df_concommed, df_no_concommed]).sort_values(by='crfnr')

## NOTE that NaN entries in this dataset involve patients where there is a register of concomitant medication, but the dates are 
#not clear and therefore not known whether it was before or after baseline.


#------------- Edinburgh Handness Inventory -------------------------------------
# OUTPUT dataframe: df_ehi
df_ehi = pd.read_excel(dir_input+'ehi_070727.xlsx').filter(regex='^(vr|f|crfnr)').filter(regex='^(?!ftl|ftr|ftu|vr1tl|vr1tr).*$')
#Get totals of right and left hand scores.
vr_l_cols = [col for col in df_ehi.columns if col.startswith('vr') and col.endswith('l')]
vr_r_cols = [col for col in df_ehi.columns if col.startswith('vr') and col.endswith('r')]

# Check for NaN values - > NaN sum, and otherwise calculate weighted sums accordingly
df_ehi['w_sum_vr_l'] = df_ehi[vr_l_cols].apply(lambda row: row.sum() / row.count() if not row.isna().all() else np.nan, axis=1)
df_ehi['w_sum_vr_r'] = df_ehi[vr_r_cols].apply(lambda row: row.sum() / row.count() if not row.isna().all() else np.nan, axis=1)

# Calculate Laterality Quotient Score with consideration for NaN values, based on: https://link.springer.com/referenceworkentry/10.1007/978-0-387-79948-3_68
df_ehi['LQS_SCORE'] = np.where((df_ehi['w_sum_vr_r'].isnull()) | (df_ehi['w_sum_vr_l'].isnull()),
                               np.nan,
                               100 * (df_ehi['w_sum_vr_r'] - df_ehi['w_sum_vr_l']) / (df_ehi['w_sum_vr_r'] + df_ehi['w_sum_vr_l']))

#Dummy coding f1-9 variables. 
# Assuming df is your DataFrame and f1-f9 are the columns to be dummy coded
f_cols =  [col for col in df_ehi.columns if col.startswith('f')]

# Map the values into categorical, otherwise it does not detect columns.
df_ehi[f_cols] = df_ehi[f_cols].replace({0: 'unknown', 1: 'left', 2: 'right'})

# Dummy code the variables using pd.get_dummies individually in a for loop.
for col in f_cols:
    df_dummies = pd.get_dummies(df_ehi[col], prefix=col)
    #If the rows are all 0's (meaning the original var was NaN) set the dummy variables to NaN.
    mask_all_zeros = (df_dummies.sum(axis=1) == 0)
    df_dummies[df_dummies.sum(axis=1) == 0] = np.nan
    #Add to dataframe and remove original.
    df_ehi = pd.concat([df_ehi, df_dummies], axis=1)
    df_ehi.drop(col, axis=1, inplace=True)

# Check for NaN values - > NaN sum, and otherwise calculate weighted sums accordingly
left_cols = [f'{f}_left' for f in f_cols]
right_cols = [f'{f}_right' for f in f_cols]
unknown_cols = [f'{f}_unknown' for f in f_cols]

df_ehi['w_sum_f_left'] = df_ehi[left_cols].apply(lambda row: row.sum() / row.count() if not row.isna().all() else np.nan, axis=1)
df_ehi['w_sum_f_right'] = df_ehi[right_cols].apply(lambda row: row.sum() / row.count() if not row.isna().all() else np.nan, axis=1)
df_ehi['w_sum_f_unknown'] = df_ehi[unknown_cols].apply(lambda row: row.sum() / row.count() if not row.isna().all() else np.nan, axis=1)

#IMPORTANT: as of now, if only a few single score has NaN we still calculate the sum of the rest and divide by
#the number of answered items. This seems to be the procedure that they followed in their own sums when 
#inspected visually, but they seem to not do it for all of them. If all scores are NaN, then the sum is set as NaN.


#------------- Global Assessment of Functioning (GAF) ---------------------------
# OUTPUT dataframe: df_gaf
df_gaf = pd.read_excel(dir_input+'gaf_070904.xlsx', sheet_name = 'Data').filter(regex='^(V1|crfnr)')

#------------- Lab data (labdata) -----------------------------------------------
# OUTPUT dataframe: df_labdata
df_labdata = pd.read_excel(dir_input+'labdata_070727.xlsx', sheet_name = 'Data').filter(regex='^(V1.*[0-9]$|crfnr)')

#Standardising glucose: 1,2,3 units not present. Standardising 4 -> 5.
#1 mmol/L = 18.01588 mg/dL, multiply the units of "4" by 18.01588.
df_labdata.loc[df_labdata['V1l12'] == 4, 'V1l04'] *= 18.01588

#Standardising cholesterol: 1,2,3 units not present. Standardising 4 -> 5.
#1 mmol/L = 38.67 mg/dL, multiply the units of "4" by 38.67.
df_labdata.loc[df_labdata['V1l13'] == 4, 'V1l05'] *= 38.67

#Standardising LDL: 1,2,3 units not present. Standardising 4 -> 5.
#1 mmol/L = 38.67 mg/dL, multiply the units of "4" by 38.67.
df_labdata.loc[df_labdata['V1l14'] == 4, 'V1l06'] *= 38.67

#Standardising HDL: 1,2,3 units not present. Standardising 4 -> 5.
#1 mmol/L = 38.67 mg/dL, multiply the units of "4" by 38.67.
df_labdata.loc[df_labdata['V1l15'] == 4, 'V1l07'] *= 38.67

#Standardising triglicerides: 1,2,3 units not present. Standardising 4 -> 5.
#1 mmol/L = 88.57 mg/dL, multiply the units of "4" by 38.67.
df_labdata.loc[df_labdata['V1l16'] == 4, 'V1l09'] *= 38.67

#Standardising prolactin: 2,4 and 5 not present. Standardising 1 -> 3 (?).
#1 IU/L = 47.76 mcg/L, multiply the units of "1" by 47.76.
df_labdata.loc[df_labdata['V1l11'] == 1, 'V1l03'] *= 47.76

# - Remove irrelevant variable of done/undone and units. 
df_labdata = df_labdata.drop(columns=['V1l10', 'V1l12', 'V1l13', 'V1l14', 'V1l15', 'V1l16', 'V1l11'])

#------------- Manchester Short Assesssment QOF --------------------------------
# OUTPUT dataframe: df_mansa
df_mansa = pd.read_excel(dir_input+'mansa_070727.xlsx', sheet_name = 'Data').filter(regex='^(V1|crfnr)')


#------------- Antypsychotic Prescribed and Initial Dose -----------------------
# OUTPUT dataframe: df_antipsychotic
df_medrec = pd.read_excel(dir_input+'medrec_070727.xlsx', sheet_name = 'Data').filter(regex='^(MR|crfnr|R1_mr02_dat|R1_mr03|R1_mr04_dat)')

#Normalise dose given based on the max and min of eah individual drug. 
for drug_num in [1, 2, 3, 4, 5]:
    max_value = df_medrec[df_medrec['MR01'] == drug_num]['R1_mr03'].max()
    min_value = df_medrec[df_medrec['MR01'] == drug_num]['R1_mr03'].min()
    df_medrec.loc[df_medrec['MR01'] == drug_num, 'NORM_DOSE_MR01'] = (df_medrec[df_medrec['MR01'] == drug_num]['R1_mr03'] - min_value) / (max_value - min_value)

# Drop unneded columns for baseline. Dropping antipsychotic given since it is present for all patients in df_antipsychotics below.
df_medrec = df_medrec.drop(['R1_mr02_dat', 'R1_mr03', 'R1_mr04_dat', 'MR01'], axis=1)

#Load the study arm (type of antipsychotic).
df_antipsychotic = pd.read_excel(dir_input+'protocol violators.xlsx', sheet_name = 'Data').filter(regex='^(StudyArm|crfnr)')
#Dummy code antipsychotic column "StudyArm", add the dummy coded columns and drop the categorical column. 
df_antipsychotic = pd.concat([df_antipsychotic, pd.get_dummies(df_antipsychotic['StudyArm'], prefix='StudyArm')], axis=1).drop('StudyArm', axis=1)

#Merge the drug given and normalised dose. 
df_antipsychotic = pd.merge(df_antipsychotic, df_medrec, on='crfnr', how='left')


#------------- Antipsychotic Prescribed Mapping to Binding Profile -----------------------
# OUTPUT dataframe: df_moa_mapping
df_medrec = pd.read_excel(dir_input+'medrec_070727.xlsx', sheet_name = 'Data').filter(regex='^(MR|crfnr|R1_mr02_dat|R1_mr03|R1_mr04_dat)')

#Normalise dose given based on the max and min of eah individual drug. 
for drug_num in [1, 2, 3, 4, 5]:
    max_value = df_medrec[df_medrec['MR01'] == drug_num]['R1_mr03'].max()
    min_value = df_medrec[df_medrec['MR01'] == drug_num]['R1_mr03'].min()
    df_medrec.loc[df_medrec['MR01'] == drug_num, 'NORM_DOSE_MR01'] = (df_medrec[df_medrec['MR01'] == drug_num]['R1_mr03'] - min_value) / (max_value - min_value)

# Drop unneded columns for baseline. Dropping antipsychotic given since it is present for all patients in df_antipsychotics below.
df_medrec = df_medrec.drop(['R1_mr02_dat', 'R1_mr04_dat', 'MR01', 'R1_mr03'], axis=1)

#Load the study arm (type of antipsychotic).
df_moa_mapping = pd.read_excel(dir_input+'protocol violators.xlsx', sheet_name = 'Data').filter(regex='^(StudyArm|crfnr)')

#Merge the drug given and normalised dose. 
df_moa_mapping = pd.merge(df_moa_mapping, df_medrec, on='crfnr', how='left')


list_of_receptors = ["D1", "D2", "D3", "D4", "D5", "H1", "H2", "H3", "H4", 
                "5-HT1", "5-HT1A", "5-HT1B", "5-HT1D", "5-HT1E", "5-HT1F", 
                "5-HT2", "5-HT2A", "5-HT2B", "5-HT2C", "5-HT3", "5-HT5A", 
                "5-HT6", "5-HT7", "α1", "α1A", "α1B", "α2", "α2A", "α2B", 
                "α2C", "β1", "β2", "M", "M1", "M2", "M3", "M4", "M5", 
                "SERT", "NET", "DAT"]

#Load the mapping. 
df_mapping = pd.read_excel('antipsychotic_mapping.xlsx', sheet_name = 'Ki')
df_action = pd.read_excel('antipsychotic_mapping.xlsx', sheet_name = 'Action')

#Convert to pKi.
df_mapping[list_of_receptors] = - np.log10(df_mapping[list_of_receptors]*1e-9)

# Multiply the agonist values in df_pKi_mapping by -1.
for column in list_of_receptors:
    df_mapping.loc[df_action[column] == 1, column] *= -1

# Get pKi's for each drug. 
df_moa_mapping.loc[df_moa_mapping['StudyArm'] == 5, list_of_receptors] = df_mapping.loc[df_mapping['ATC Code'] == 'N05AE04', list_of_receptors].values
df_moa_mapping.loc[df_moa_mapping['StudyArm'] == 4, list_of_receptors] = df_mapping.loc[df_mapping['ATC Code'] == 'N05AL05', list_of_receptors].values
df_moa_mapping.loc[df_moa_mapping['StudyArm'] == 3, list_of_receptors] = df_mapping.loc[df_mapping['ATC Code'] == 'N05AH04', list_of_receptors].values
df_moa_mapping.loc[df_moa_mapping['StudyArm'] == 2, list_of_receptors] = df_mapping.loc[df_mapping['ATC Code'] == 'N05AH03', list_of_receptors].values
df_moa_mapping.loc[df_moa_mapping['StudyArm'] == 1, list_of_receptors] = df_mapping.loc[df_mapping['ATC Code'] == 'N05AD01', list_of_receptors].values

#Drop unwanted columns
df_moa_mapping = df_moa_mapping.drop(['StudyArm', 'NORM_DOSE_MR01'], axis=1)


#------------- MINI Psychiatric Diagnoses --------------------------------------
# OUTPUT dataframe: df_miniplus
df_miniplus = pd.read_excel(dir_input+'miniplus_070727.xlsx', sheet_name = 'Data').filter(regex='^(V1|crfnr)')
#Remove dates of visit.
df_miniplus  = df_miniplus.drop(columns = ['V1miint_dat'])

# ------------ Neurocognitive scores -------------------------------------------
# OUTPUT dataframe: df_neuro
df_neuro = pd.read_excel(dir_input+'neuro_070727.xlsx', sheet_name = 'Data').filter(regex='^(V1|crfnr)')
#Remove columns with "info not obtained"
df_neuro  = df_neuro.drop(columns = ['V1NEU001', 'V1NEU007', 'V1NEU010', 'V1NEU047'])
#Add trailmaking difference.
df_neuro['V1NEU04MINUS01'] = df_neuro['V1NEU04'] - df_neuro['V1NEU01']
df_neuro['V1NEU05MINUS02'] = df_neuro['V1NEU05'] - df_neuro['V1NEU02']

#Add total across 5 trials.
df_neuro['V1NESUMCORR'] = df_neuro[['V1NEU47', 'V1NEU50', 'V1NEU53', 'V1NEU56', 'V1NEU59']].sum(axis=1)
df_neuro['V1NESUMINTRU'] = df_neuro[['V1NEU48', 'V1NEU51', 'V1NEU54', 'V1NEU57', 'V1NEU60']].sum(axis=1)
df_neuro['V1NESUMREPE'] = df_neuro[['V1NEU49', 'V1NEU52', 'V1NEU55', 'V1NEU58', 'V1NEU61']].sum(axis=1)
#If all rows are NaN, then SUM is also NaN. 
nan_rows = df_neuro[['V1NEU49', 'V1NEU52', 'V1NEU55', 'V1NEU58', 'V1NEU61']].isna().all(axis=1)
df_neuro.loc[nan_rows, ['V1NESUMCORR', 'V1NESUMINTRU', 'V1NESUMREPE']] = np.nan

# ------------ Other Antipsychotics -------------------------------------------
# OUTPUT dataframe: df_otherantip
def categorize_drug(atcode):
    if type(atcode) == str:
        if atcode == 'N05AX08':
            return 'OTHERANTIPSY_Risperidone'
        elif atcode == 'N05AH02':
            return 'OTHERANTIPSY_Clozapine'
        elif atcode == 'N05AH03':
            return 'OTHERANTIPSY_Olanzapine'
        elif atcode == 'N05AH04':
            return 'OTHERANTIPSY_Quetiapine'
        elif atcode == 'N05AL01':
            return 'OTHERANTIPSY_Sulpiride'
        elif atcode == 'N05AD01':
            return 'OTHERANTIPSY_Haloperidol'
        elif atcode == 'N05AF05':
            return 'OTHERANTIPSY_Zuclopenthixol'
        elif atcode == 'N05AB03':
            return 'OTHERANTIPSY_Perphenazine'
        elif atcode == 'N05AA01':
            return 'OTHERANTIPSY_Chlorpromazine'
        elif atcode == 'N05AA02':
            return 'OTHERANTIPSY_Levomepromazine'
        elif atcode == 'N05AL05':
            return 'OTHERANTIPSY_Amisulpride'
        elif atcode == 'N05AX07':
            return 'OTHERANTIPSY_Prothipendyl'
        elif atcode == 'N05AX09':
            return 'OTHERANTIPSY_Clotiapine'
        elif atcode == 'N05AX12':
            return 'OTHERANTIPSY_Aripiprazole'
        elif atcode == 'N05AB10':
            return 'OTHERANTIPSY_Perazine'
        elif atcode == 'N05AF01':
            return 'OTHERANTIPSY_Flupentixol'
        elif atcode == 'N05AG03':
            return 'OTHERANTIPSY_Penfluridol'
        elif atcode == 'N05AG02':
            return 'OTHERANTIPSY_Pimozide'
        elif atcode == 'N05AA03':
            return 'OTHERANTIPSY_Promazine'
        elif atcode == 'N05AB02':
            return 'OTHERANTIPSY_Fluphenazine'
        elif atcode == 'N05AH01':
            return 'OTHERANTIPSY_Loxapine'
        elif atcode == 'N05AE04':
            return 'OTHERANTIPSY_Ziprasidone'
        elif atcode == 'N05AF03':
            return 'OTHERANTIPSY_Chlorprothixene'
        elif atcode == 'N05AB06':
            return 'OTHERANTIPSY_Trifluoperazine'
        elif atcode == 'N05AA06':
            return 'OTHERANTIPSY_Cyamemazine'
        elif atcode == 'N05AD05':
            return 'OTHERANTIPSY_Pipamperone'
        elif atcode == 'N05AX11':
            return 'OTHERANTIPSY_Zotepine'
        elif atcode == 'N05AC02':
            return 'OTHERANTIPSY_Thioridazine'
        elif atcode == 'N05AB04':
            return 'OTHERANTIPSY_Prochlorperazine'
        else:
            return 'OTHERANTIPSY_OTHER'
    else:
        return None

df_otherantip = pd.read_excel(dir_input+'Other_antipsych_070727.xlsx', sheet_name = 'Data', usecols = ['crfnr','dStart1', 'dEind1', 'atcode1'])
#Extract dates of baseline visit. 
df_v1_date = pd.read_excel(dir_input+'v1_070727.xlsx', sheet_name = 'Data').filter(regex='^(rr001|crfnr)')
#Left merge. 
df_otherantip = pd.merge(df_otherantip, df_v1_date, on='crfnr', how='left')
# Create "other antipsychotic before" variable, when date of V1 is after start of first other antipsychotic medication.
df_otherantip['OTHERANTIPSY_BEFORE'] = (df_otherantip['rr001_dat'] >= df_otherantip['dStart1']).astype(int) 
# Set OTHERANTIPSY_BEFORE to NaN where R1_pi02_dat is NaN or NaT.
df_otherantip.loc[df_otherantip['dStart1'].isna(), 'OTHERANTIPSY_BEFORE'] = np.nan
# Story patient ID and CONCOMMED_BEFORE.
df_otherantip = df_otherantip[['crfnr','OTHERANTIPSY_BEFORE']]


# Define the list of other antipsychotic administrations (it goes up to 25)
medications = [str(i) for i in range(1, 30)]

#Unique names of other antipsychotics present in the dataset. 
drug_names = ['OTHERANTIPSY_' + drug for drug in [
    'Risperidone', 'Clozapine', 'Olanzapine', 'Quetiapine', 'Sulpiride', 'Haloperidol',
    'Zuclopenthixol', 'Perphenazine', 'Chlorpromazine', 'Levomepromazine', 'Amisulpride',
    'Prothipendyl', 'Clotiapine', 'Gabapentin', 'Perazine', 'Flupentixol', 'Penfluridol',
    'Pimozide', 'Promazine', 'Fluphenazine', 'Loxapine', 'Ziprasidone', 'Chlorprothixene',
    'Trifluoperazine', 'Cyamemazine', 'Pipamperone', 'Zotepine', 'Thioridazine', 'Prochlorperazine']]


list_of_TYPE = []

for medication in medications:
    # Read other antipsychotic medication data
    cols = ['crfnr','dStart'+medication, 'dEind'+medication, 'atcode'+medication]
    df_otherantip_each = pd.read_excel(dir_input+'Other_antipsych_070727.xlsx', sheet_name='Data', usecols = cols)

    # Left merge
    df_otherantip_each = pd.merge(df_otherantip_each, df_v1_date, on='crfnr', how='left')

    # Create "other antipsychotic medication before" variable.
    df_otherantip_each['OTHERANTIPSY_BEFORE'] = (df_otherantip_each['rr001_dat'] >= df_otherantip_each['dStart'+medication]).astype(int)
    
    #Mapping of codes to drug name. 
    for drug in drug_names:
        df_otherantip_each[drug] = df_otherantip_each['atcode'+medication].apply(lambda x: 1 if categorize_drug(x) == drug else 0) 

    #Set dummy codes for types of drugs to 0 if CONCOMMED_BEFORE is 0.
    df_otherantip_each.loc[df_otherantip_each['OTHERANTIPSY_BEFORE'] == 0, drug_names] = 0
    #Append it to the list. 
    list_of_TYPE.append(df_otherantip_each[drug_names])

    
#Record other antipsychotic medication names and overlap them. If one type is prescribed more than once, it is stil set to 1. 
df_otherantip.loc[:, drug_names] = np.where(np.sum(list_of_TYPE, axis=0)>= 1, 1, 0)
# If OTHERANTIPSY_BEFORE is n.nan, then all interventions are nan (since it is not clear if v1 was before or after).
df_otherantip.loc[df_otherantip_each['OTHERANTIPSY_BEFORE'].isna(), drug_names] = np.nan


#IMPORTANT NEW ADDITION: If there is no record of other antipsychotics, then set OTHERANTIPSY_BEFORE and individual antipsychotic to 0.
df_v1 = pd.read_excel(dir_input+'v1_070727.xlsx', sheet_name = 'Data').filter(regex='^(crfnr)')
# Get entries of patients not in df_otherantip and set all variables to 0.
df_no_otherantip = df_v1[~df_v1['crfnr'].isin(df_otherantip['crfnr'])].copy()
df_no_otherantip[['OTHERANTIPSY_BEFORE', drug_names]] = 0
# Concatenate entries in the register and not in the register.
df_otherantip = pd.concat([df_otherantip, df_no_otherantip]).sort_values(by='crfnr')

## NOTE that NaN entries in this dataset involve patients where there is a register of other antipsychotics, but the dates are 
#not clear and therefore not known whether it was before, during or after baseline.


# ------------ PANSS -----------------------------------------------------------
# OUTPUT dataframe: df_panss
df_panss = pd.read_excel(dir_input+'pansa_070727.xlsx', sheet_name = 'Data').filter(regex='^(V1|crfnr)')
# List of PANSS score columns.
positive_columns = ['V1pa01', 'V1pa02', 'V1pa03', 'V1pa04', 'V1pa05', 'V1pa06', 'V1pa07']
negative_columns = ['V1pa08', 'V1pa09', 'V1pa10', 'V1pa11', 'V1pa12', 'V1pa13', 'V1pa14']
general_columns = ['V1pa15', 'V1pa16', 'V1pa17', 'V1pa18', 'V1pa19', 'V1pa20', 'V1pa21', 'V1pa22', 'V1pa23', 'V1pa24', 'V1pa25', 'V1pa26', 'V1pa27', 'V1pa28', 'V1pa29', 'V1pa30']

# Add new columns for the sum of positive, negative, and general scores
df_panss['V1ptotal'] = df_panss[positive_columns].sum(axis=1)
df_panss['V1ntotal'] = df_panss[negative_columns].sum(axis=1)
df_panss['V1gtotal'] = df_panss[general_columns].sum(axis=1)

# Set the sum values to NaN if all individual values are NaN
df_panss.loc[df_panss[positive_columns].isna().all(axis=1), 'V1ptotal'] = np.nan
df_panss.loc[df_panss[negative_columns].isna().all(axis=1), 'V1ntotal'] = np.nan
df_panss.loc[df_panss[general_columns].isna().all(axis=1), 'V1gtotal'] = np.nan

#Calculate total score. 
df_panss['V1total'] = df_panss[['V1ptotal', 'V1ntotal', 'V1gtotal']].sum(axis = 1)

# Set the total score to NaN if all individual values are NaN
df_panss.loc[df_panss[['V1ptotal', 'V1ntotal', 'V1gtotal']].isna().all(axis=1), 'V1total'] = np.nan


#------------- DSM Psychosis ---------------------------------------------------
# OUTPUT dataframe: df_pd
df_pd = pd.read_excel(dir_input+'PD_070727.xlsx', sheet_name = 'Data').filter(regex='^(code|crfnr)')
#Convert NaNs to 0 if any diagnose is given (mask = True), otherwise leave as NaN (mask = False).
code_list = ['code1', 'code2', 'code3', 'code4', 'code5', 'code6', 'code7']
mask = df_pd[code_list].eq(1.0).any(axis=1)
df_pd.loc[mask, code_list] = df_pd.loc[mask, code_list].fillna(0)

#------------- Protocol Violators ----------------------------------------------
# OUTPUT dataframe: df_violators
# Load just the protocol violators.
df_violators = pd.read_excel(dir_input+'protocol violators.xlsx', sheet_name = 'Data').filter(regex='^(VIOLATOR|crfnr)')

#------------- Hospitalisations ------------------------------------------------
# OUTPUT dataframe: df_hosp

#Extract first hospitalisation of patients. 
df_hosp = pd.read_excel(dir_input+'psychhospitrec_070727.xlsx', sheet_name = 'Data').filter(regex='^(R1|crfnr)')
#Extract dates of baseline visit. 
df_v1_date = pd.read_excel(dir_input+'v1_070727.xlsx', sheet_name = 'Data').filter(regex='^(rr001|crfnr)')
#Left merge. 
df_hosp  = pd.merge(df_hosp, df_v1_date, on='crfnr', how='left')
#Create "hospitalised before" variable, when date of V1 is after start of first hospitalisation.
df_hosp['HOSP_BEFORE'] = (df_hosp['rr001_dat'] >= df_hosp['R1_phr01_dat']).astype(int)
# Remove dates. 
df_hosp = df_hosp.drop(columns = ['rr001_dat', 'R1_phr01_dat', 'R1_phr02_dat'])
## IMPORTANT: I visually inspected that if doing the same analysis for R2 (second hospitalisation), 
#there are no cases of these happening before V1, so the only hospitalisation that can happen before V1 is R1.

#IMPORTANT NEW ADDITION: If there is no record of hospitalisations, then set HOSP_BEFORE to 0.
df_v1 = pd.read_excel(dir_input+'v1_070727.xlsx', sheet_name = 'Data').filter(regex='^(crfnr)')
# Get entries of patients not in df_hosp and set all variables to 0.
df_no_hosp = df_v1[~df_v1['crfnr'].isin(df_hosp['crfnr'])].copy()
df_no_hosp['HOSP_BEFORE'] = 0
# Concatenate entries in the register and not in the register.
df_hosp = pd.concat([df_hosp, df_no_hosp]).sort_values(by='crfnr')

## NOTE that NaN entries in this dataset involve patients where there is a register of hospitalisation, but the dates are 
#not clear and therefore not known whether it was before, during or after baseline.


#------------- Interventions ---------------------------------------------------
# OUTPUT dataframe: df_interv
#Extract first intervention. 
df_interv = pd.read_excel(dir_input+'psychintervrec_070727.xlsx', sheet_name = 'Data').filter(regex='^(R1|crfnr)(?!.*_text$)')
#Extract dates of baseline visit. 
df_v1_date = pd.read_excel(dir_input+'v1_070727.xlsx', sheet_name = 'Data').filter(regex='^(rr001|crfnr)')
#Left merge. 
df_interv  = pd.merge(df_interv, df_v1_date, on='crfnr', how='left')
# Create "intervention before" variable, when date of V1 is after start of first intervention.
df_interv['INTERV_BEFORE'] = (df_interv['rr001_dat'] >= df_interv['R1_pi02_dat']).astype(int) 
# Set INTERV_BEFORE to NaN where R1_pi02_dat is NaN or NaT.
df_interv.loc[df_interv['R1_pi02_dat'].isna(), 'INTERV_BEFORE'] = np.nan
#Drop all columns except for INTERV_BEFORE and ID. 
df_interv = df_interv[['crfnr','INTERV_BEFORE']]
#IMPORTANT: Visually inspected that in all cases where R2 (second intervention) happens before V1, there is a R1 (first intervention) that also happens before V1. 
## IF WE WANTED NUMBER OF INTERVENTIONS AND TYPES OF INTERVENTIONS BEFORE AND DURING V1:
# Define the list of interventions (R1, R2, R3, R4...)
interventions = ['R1', 'R2', 'R3', 'R4', 'R5', 'R6', 'R7', 'R8', 'R9']

names_of_interventions = ['INTERV_T1', 'INTERV_T2', 'INTERV_T3', 'INTERV_T4', 'INTERV_T5', 'INTERV_T6', 'INTERV_T7', 'INTERV_T8', 'INTERV_T9']

list_of_TYPE = []

for intervention in interventions:
    # Read intervention data
    df_interv_each = pd.read_excel(dir_input+'psychintervrec_070727.xlsx', sheet_name='Data').filter(regex=f'^({intervention}|crfnr)(?!.*_text$)')

    # Left merge
    df_interv_each = pd.merge(df_interv_each, df_v1_date, on='crfnr', how='left')

    # Create "intervention before" variable
    df_interv_each[f'INTERV_BEFORE_{intervention}'] = (df_interv_each['rr001_dat'] >= df_interv_each[f'{intervention}_pi02_dat']).astype(int)

    # If the intervention is not before V1, set the intervention type to 0
    df_interv_each.loc[df_interv_each[f'INTERV_BEFORE_{intervention}'] != 1, [f'{intervention}_pi01_1', f'{intervention}_pi01_2', f'{intervention}_pi01_3', f'{intervention}_pi01_4', f'{intervention}_pi01_5', f'{intervention}_pi01_6', f'{intervention}_pi01_7', f'{intervention}_pi01_8', f'{intervention}_pi01_9']] = 0
    #Append the type of interventions. 
    list_of_TYPE.append(df_interv_each[[f'{intervention}_pi01_1', f'{intervention}_pi01_2', f'{intervention}_pi01_3', f'{intervention}_pi01_4', f'{intervention}_pi01_5', f'{intervention}_pi01_6', f'{intervention}_pi01_7', f'{intervention}_pi01_8', f'{intervention}_pi01_9']])

#Record number of interventions and overlap of recorded interventions in all visits. 
df_interv[names_of_interventions] = np.where(np.sum(list_of_TYPE, axis=0)>= 1, 1, 0)

# If INTERV_BEFORE is n.nan, then all interventions are nan (since it is not clear if v1 was before or after).
df_interv.loc[df_interv['INTERV_BEFORE'].isna(), names_of_interventions] = np.nan

#IMPORTANT NEW ADDITION: If there is no record of interventions, then set INTERV_BEFORE and individual interventions to 0.
df_v1 = pd.read_excel(dir_input+'v1_070727.xlsx', sheet_name = 'Data').filter(regex='^(crfnr)')
# Get entries of patients not in df_interv and set all variables to 0.
df_no_interv = df_v1[~df_v1['crfnr'].isin(df_interv['crfnr'])].copy()
df_no_interv[['INTERV_BEFORE', names_of_interventions]] = 0
# Concatenate entries in the register and not in the register.
df_interv = pd.concat([df_interv, df_no_interv]).sort_values(by='crfnr')

## NOTE that NaN entries in this dataset involve patients where there is a register of intervention, but the dates are 
#not clear and therefore not known whether it was before, during or after baseline.


# ------------ St. Hans Rating Scale -------------------------------------------
# OUTPUT dataframe: df_shrs
df_shrs_scores = pd.read_excel(dir_input+'shrs_070727.xlsx', sheet_name = 'Data').filter(regex='^(V1|crfnr)')
#Load the diagnoses from StHans.xlsx
df_shrs_diag = pd.read_excel(dir_input+'StHans.xlsx', sheet_name = 'Data').filter(regex='^(V1|crfnr)')
#Merge it with the raw scores of symptoms. 
df_shrs = df_shrs_scores.merge(df_shrs_diag, how = 'outer')

#------------- Substance Abuse -------------------------------------------------
# OUTPUT dataframe: df_sub_abuse
# Remove columns with "specify" info, columns with "_s".
df_sub_abuse = pd.read_excel(dir_input+'sub_abuse_070727.xlsx', sheet_name = 'Data').filter(regex='^(V1|crfnr)').filter(regex='[^s]$')
# Standardise frequency columns (day/week/month) to day frequency for specific substances.  
time_cols = ['V1SAL04', 'V1SAL09', 'V1SAL12', 'V1SAL15', 'V1SAL18', 'V1SAL21', 'V1SAL24']
freq_cols = ['V1SAL05', 'V1SAL10', 'V1SAL13', 'V1SAL16', 'V1SAL19', 'V1SAL22', 'V1SAL25']
# 1 means day, 2 means week, 3 means month.
freq_divisor_mapping = {1.0: 1, 2.0: 7, 3.0: 30}
#Normalise "times" columns
for time_col, freq_col in zip(time_cols, freq_cols):
    df_sub_abuse[time_col] = df_sub_abuse[time_col] / df_sub_abuse[freq_col].map(freq_divisor_mapping)
    # Drop the original frequency column, not needed any more. 
    df_sub_abuse.drop(columns=[freq_col], inplace=True)

#------------- Side Effects ----------------------------------------------------
# OUTPUT dataframe: df_uku
df_uku = pd.read_excel(dir_input+'uku_070727.xlsx', sheet_name = 'Data').filter(regex='^(V1|crfnr)')
# Remove "0" -> Not assessed from some variables. 
list_of_variables = ['V1UKU08', 'V1UKU09', 'V1UKU10', 'V1UKU11', 'V1UKU12', 'V1UKU13', 'V1UKU21', 'V1UKU22', 'V1UKU23', 'V1UKU24', 'V1UKU25', 'V1UKU26', 'V1UKU27']
df_uku[list_of_variables] = df_uku[list_of_variables].replace(0, np.nan)

#------------- Visit 1, demographics (and others) and covariants -------------------------------------------
# OUTPUT dataframe: df_prognosis, df_covariates, df_demographics
df_v1 = pd.read_excel(dir_input+'v1_070727.xlsx', sheet_name = 'Data')
df_age = pd.read_excel(dir_input+'age.xlsx', sheet_name = 'Data')

#Define the selected columns from the dataset. 
cols_for_demographics = ['crfnr','a003', 'a015_t', 'a022','a016', 'a017', 'a023', 'a024', 'a025',
                        'a027', 'a030','a031', 'a032', 'a033', 'a034', 'a035', 'a036',
                        'a037', 'a038', 'a039', 'a040', 'a041', 'a042', 'a043', 'a044', 
                         'a045', 'a046', 'a047', 'a048', 'a049', 'a050', 'a050_2', 'a052', 
                         'a053', 'a054', 'a055', 'a056', 'a057', 'a057_2','a059', 'a062', 'a063',
                         'e02', 'e03', 'ph01', 'ph03', 'ph02', 'ph05', 'ph04', 'ph06', 
                        'a060_1', 'a060_2', 'a060_3', 'a060_4', 'a060_5']

cols_for_dummy_coding_demographics = ['a015_t', 'a022', 'a031', 'a033', 'a034', 'a035', 'a037', 'a038', 'a039', 'a040', 'a059']


#Extract the clinitian prognosis variable. 
df_prognosis = df_v1[['crfnr', 'p01']]

#Extract site as covariate. 
df_covariates = df_v1[['crfnr', 'sitecode']]
#Dummy code the covariates and add them to the dataframe without droping original.
df_covariates = pd.concat([df_covariates, pd.get_dummies(df_covariates['sitecode'], columns=['sitecode'], prefix = 'sitecode').astype(int)], axis = 1)


#Extraction of selected demographic variables; raw. 
df_demographics = df_v1[cols_for_demographics].copy()

#Case "a031" has nan values where it should be 8 -> 'unemployed' because they have answered "a030" of current occupation as "no". 
#So changing nan for 8 when the other column is 0. 
df_demographics.loc[df_demographics['a030'] == 0, 'a031'] = 8
#Same applies to columns a032 and a033. 
df_demographics.loc[df_demographics['a032'] == 0, 'a033'] = 8

# Normalisation of income amount per landcode (country) based on the max and min of eah individual country. 
df_demographics['a062'] = pd.to_numeric(df_demographics['a062'], errors = 'coerce')
df_demographics['a062_norm'] = df_demographics['a062']
for landcode_num in df_v1['landcode'].unique():
    max_value = df_demographics[df_v1['landcode'] == landcode_num]['a062'].max()
    min_value = df_demographics[df_v1['landcode'] == landcode_num]['a062'].min()
    df_demographics.loc[df_v1['landcode'] == landcode_num, 'a062_norm'] = (df_demographics[df_v1['landcode'] == landcode_num]['a062'] - min_value) / (max_value - min_value)

#Drop the original variable.
df_demographics['a062'] = df_demographics['a062_norm']
#Retain the location of the "a062" variable. 
df_demographics.drop('a062_norm', axis = 1, inplace = True)
df_demographics.rename(columns={'a062': 'a062_norm'}, inplace=True)

#Calculate BMI from weight and height (metric system).
df_demographics['phbmi'] = df_demographics['ph02'] / (df_demographics['ph01']**2)

# Dummy coding of selected variables. 
for col in cols_for_dummy_coding_demographics:
    df_dummies = pd.get_dummies(df_demographics[col], prefix=col).astype(int)
    #If the rows are all 0's (meaning the original var was NaN) set the dummy variables to NaN.
    mask_all_zeros = (df_dummies.sum(axis=1) == 0)
    df_dummies[df_dummies.sum(axis=1) == 0] = np.nan
    #Add to dataframe and remove original.
    # Insert dummy columns at the same location as the original variable.
    col_index = df_demographics.columns.get_loc(col)
    df_demographics = pd.concat([df_demographics.iloc[:, :col_index], df_dummies, df_demographics.iloc[:, col_index+1:]], axis=1)

# Merge age to df_demographics. 
df_demographics = df_demographics.merge(df_age, how = 'left', on = 'crfnr')
```

In [12]:

```
################################################################################
#Storing Data and Merging
################################################################################
# Sergio Mena Ortega, 2023


#Writing individual datasets into excel.
## IMPORTANT: violators not removed, bear in mind they will have to be removed if using these.
df_cam.to_excel(dir_output+'Individual Datasets/can.xlsx', index = None) #
df_cdss.to_excel(dir_output+'Individual Datasets/cdss.xlsx', index = None) #
df_cgi.to_excel(dir_output+'Individual Datasets/cgi.xlsx', index = None) #
df_concommed.to_excel(dir_output+'Individual Datasets/concommed.xlsx', index = None) #
df_ehi.to_excel(dir_output+'Individual Datasets/ehi.xlsx', index = None) #
df_gaf.to_excel(dir_output+'Individual Datasets/gaf.xlsx', index = None) #
df_labdata.to_excel(dir_output+'Individual Datasets/labdata.xlsx', index = None) #
df_mansa.to_excel(dir_output+'Individual Datasets/mansa.xlsx', index = None) #
df_antipsychotic.to_excel(dir_output+'Individual Datasets/antipsychotics.xlsx', index = None) #
df_moa_mapping.to_excel(dir_output+'Individual Datasets/moa_mapping.xlsx', index = None)
df_miniplus.to_excel(dir_output+'Individual Datasets/miniplus.xlsx', index = None) #
df_neuro.to_excel(dir_output+'Individual Datasets/neuro.xlsx', index = None) #
df_otherantip.to_excel(dir_output+'Individual Datasets/otherantipsychotic.xlsx', index = None) #
df_panss.to_excel(dir_output+'Individual Datasets/panss.xlsx', index = None) #
df_violators.to_excel(dir_output+'Individual Datasets/protocolviolators.xlsx', index = None)
df_hosp.to_excel(dir_output+'Individual Datasets/hospitalisations.xlsx', index = None) #
df_interv.to_excel(dir_output+'Individual Datasets/interventions.xlsx', index = None) #
df_shrs.to_excel(dir_output+'Individual Datasets/shrs.xlsx', index = None) #
df_sub_abuse.to_excel(dir_output+'Individual Datasets/sub_abuse.xlsx', index = None) #
df_uku.to_excel(dir_output+'Individual Datasets/uku.xlsx', index = None) #
df_prognosis.to_excel(dir_output+'Individual Datasets/prognosis.xlsx', index = None) #
df_covariates.to_excel(dir_output+'Individual Datasets/covariates.xlsx', index = None) #
df_demographics.to_excel(dir_output+'Individual Datasets/demographics.xlsx', index = None) #

#Merging data into 4 categories: clinical+sociodemographic, cognitive, QoL and lab data (df_cdss, df_concommed, df_ehi, df_gaf, df_antipsychotic,
#df_miniplus, df_otherantip, df_panss, df_hosp, df_interv, df_shrs, df_sub_abuse, df_uku, df_demographics).

#Get violators IDs.
violators = df_violators.loc[df_violators['VIOLATOR'] == 1, 'crfnr']

#Get the original ids. 
df_id = pd.read_excel(dir_input+'v1_070727.xlsx', sheet_name = 'Data', usecols = ['crfnr'])

#Clinical and sociodemographic data category.
df_clin_and_socio = df_id.copy()
list_of_dataframes = [df_cdss, df_cgi, df_concommed, df_ehi, df_gaf, df_antipsychotic, df_miniplus, df_otherantip, df_panss, df_hosp, df_interv, df_shrs, df_sub_abuse, df_uku, df_demographics, df_pd] 
for data in list_of_dataframes:
    df_clin_and_socio = pd.merge(df_clin_and_socio, data, on='crfnr', how='left')
#Remove protocol violator entries. 
df_clin_and_socio = df_clin_and_socio[~df_clin_and_socio['crfnr'].isin(violators)]
#Save to excel.    
df_clin_and_socio.to_excel(dir_output+'Merged Datasets/clinical_and_sociodemographic.xlsx', index = False)

#MOA mapping category (df_moa_mapping). 
df_moa_mapping_data = pd.merge(df_id, df_moa_mapping, on='crfnr', how='left')
#Remove protocol violator entries. 
df_moa_mapping_data = df_moa_mapping_data[~df_moa_mapping_data['crfnr'].isin(violators)]
#Save to excel.    
df_moa_mapping_data.to_excel(dir_output+'Merged Datasets/moa_mapping.xlsx', index = False)

#Blood data category (df_labdata). 
df_blood_data = pd.merge(df_id, df_labdata, on='crfnr', how='left')
#Remove protocol violator entries. 
df_blood_data = df_blood_data[~df_blood_data['crfnr'].isin(violators)]
#Save to excel.    
df_blood_data.to_excel(dir_output+'Merged Datasets/lab_data.xlsx', index = False)


#Cognitive category (df_neuro). 
df_cognitive = pd.merge(df_id, df_neuro, on='crfnr', how='left')
#Remove protocol violator entries. 
df_cognitive = df_cognitive[~df_cognitive['crfnr'].isin(violators)]
#Save to excel.    
df_cognitive.to_excel(dir_output+'Merged Datasets/cognitive.xlsx', index = False)


##Quality of life category (df_mansa, df_cam). 
df_quality_of_life = pd.merge(df_id, df_cam, on='crfnr', how='left')
df_quality_of_life = pd.merge(df_quality_of_life, df_mansa, on='crfnr', how='left')
#Remove protocol violator entries. 
df_quality_of_life = df_quality_of_life[~df_quality_of_life['crfnr'].isin(violators)]
#Save to excel.    
df_quality_of_life.to_excel(dir_output+'Merged Datasets/quality_of_life.xlsx', index = False)


##Clinicians prognosis (df_prognosis). 
df_prognosis = pd.merge(df_id, df_prognosis, on='crfnr', how='left')
#Remove protocol violator entries. 
df_prognosis = df_prognosis[~df_prognosis['crfnr'].isin(violators)]
#Save to excel.    
df_prognosis.to_excel(dir_output+'Merged Datasets/prognosis.xlsx', index = False)

##Covariates (df_covariates).
df_covariates = pd.merge(df_id, df_covariates, on='crfnr', how='left')
#Remove protocol violator entries. 
df_covariates = df_covariates[~df_covariates['crfnr'].isin(violators)]
#Save to excel.    
df_covariates.to_excel(dir_output+'Merged Datasets/covariates.xlsx', index = False)
```
