# Supplementary material for: AI-based prediction of depression symptomatology in first-episode psychosis patients: insights from the EUFEST and RAISE-ETP clinical trials
Source: Psychol Med. 2025 Jul 30;55:e221. doi: 10.1017/S0033291725100950 (PMC12341035; doi:10.1017/S0033291725100950)
Supplement: Mena et al. supplementary material 2 — Mena et al. supplementary material [file S0033291725100950sup002.zip › supporting files/baseline_extraction_raiseetp.html]

baseline\_extraction


In [20]:

```
import pandas as pd
import numpy as np

pd.set_option('display.max_rows', 5)
pd.set_option('display.max_columns', None)

#Folder name with original input data.
dir_input = 'Raw data/'

#Folder name for output data.
dir_output = 'Baseline/'
```

In [15]:

```
################################################################################
#Data Extraction
################################################################################
# Sergio Mena Ortega, 2024


#------------- PANSS ---------------------------------------------
# OUTPUT dataframe: df_panss
df_panss = pd.read_excel(dir_input + 'panss01.xlsx', skiprows=[1]).filter(regex = "^(src_subject_id$|panss_total|pos_|neg_|gps_|visit$)")

# Filter the visit, just baseline, and drop the visit column.  
df_panss = df_panss[df_panss['visit'] == 'B'].drop(columns = 'visit')

# Sum positive scores, negative scores, and general scores (gps) separately
df_panss['panss_ptotal'] = df_panss.filter(like='pos_').sum(axis=1)
df_panss['panss_ntotal'] = df_panss.filter(like='neg_').sum(axis=1)
df_panss['panss_gtotal'] = df_panss.filter(like='gps_').sum(axis=1)
df_panss
#-----------------------------------------------------------------

#------------- Clinical Global impression (CGI) ------------------
# OUTPUT dataframe: df_cgi
df_cgi = pd.read_excel(dir_input + 'cgis01.xlsx', skiprows=[1]).filter(regex = "^(src_subject_id$|cs16$|visit$)")

# Filter the visit, just baseline, and drop the visit column.  
df_cgi = df_cgi[df_cgi['visit'] == 'B'].drop(columns = 'visit')

#-----------------------------------------------------------------

#------------- Calgary Depression Scale for Schizophrenia --------
# OUTPUT dataframe: df_cdss
df_cdss = pd.read_excel(dir_input + 'clgry01.xlsx', skiprows=[1]).filter(regex = "^(src_subject_id$|calg|visit$)")

# Filter the visit, just baseline, and drop the visit column.  
df_cdss = df_cdss[df_cdss['visit'] == 'B'].drop(columns = 'visit')

##Remove irrelevant cols. 
df_cdss.drop(columns = ['calg_s1', 'calg10', 'calg_s2'], inplace = True)


##IMPORTANT: calgary is from 0 to 3, here it seems to start from 1, so we remove 1 to each col.
calg_columns = ['calg1', 'calg2', 'calg3', 'calg4', 'calg5', 'calg6', 'calg7', 'calg8', 'calg9']
df_cdss[calg_columns] = df_cdss[calg_columns].sub(1)

# Recalculate the total in the calg_ts column
df_cdss['calg_ts'] = df_cdss[calg_columns].sum(axis=1)

#-----------------------------------------------------------------

#------------- Quality of Life  ----------------------------------
# OUTPUT dataframe: df_qol
df_qol = pd.read_excel(dir_input + 'qol01.xlsx', skiprows=[1])

# Filter selected columns
cols_to_use = ['src_subject_id', 'visit', "qol02", "qol03", "qol04", "qol05", "qol06", "qol07", "qol08", "qol09", "qol10", "qol11",
    "qol12", "qol13", "qol14", "qol15", "qol16", "qol17", "qol18", "qol19", "qol20", "qol21", "qol22", "intr_rel", "inst_rol", "intr_fou", 
    "com_obj", "qol_tot", "d1reltsx", "d2roltsx","d3foutsx", "d4comtsx", "qlstsx", "a1reltsx", "a2roltsx", "a3foutsx", "a1relts", "a2rolts",
    "a3fouts"]


df_qol = df_qol[cols_to_use].copy()

# Filter the visit, just baseline, and drop the visit column.  
df_qol = df_qol[df_qol['visit'] == 'B'].drop(columns = 'visit')

## set 9 -> NaN in selected columns.
for col in ["qol02", "qol03", "qol04", "qol05", "qol06", "qol07", "qol08", "qol09", "qol10", "qol11", "qol12", "qol13", "qol14", "qol15", "qol16", "qol17", "qol18", "qol19", "qol20", "qol21", "qol22"]:
    df_qol.loc[df_qol["qol02"] == 9, col] = np.nan

#-----------------------------------------------------------------

#------------- Study compliance  ---------------------------------
# OUTPUT dataframe: df_compliance

df_compliance = pd.read_excel(dir_input + 'Intent_to_Attend.xlsx')

df_compliance = df_compliance[['src_Subject_ID','Visit', 'How likely is it that you will complete the study?', 'How likely is it that you will attend the next in-person assessment?']].copy()

# Define the new column names
new_column_names = {
    'src_Subject_ID': 'src_subject_id',
    'Visit': 'visit',
    'How likely is it that you will complete the study?': 'completion_likeliness', 
    'How likely is it that you will attend the next in-person assessment?': 'next_assessment_likeliness'
}

# Rename the columns
df_compliance = df_compliance.rename(columns=new_column_names)

# Filter the visit, just baseline, and drop the visit column.  
df_compliance = df_compliance[df_compliance['visit'] == 'B'].drop(columns = 'visit')

#Dummy code categorical cols. 
dummy_coded_df_compliance = pd.get_dummies(df_compliance, columns=['completion_likeliness', 'next_assessment_likeliness'])
df_compliance.drop(columns = ['completion_likeliness', 'next_assessment_likeliness'], inplace = True)
df_compliance = df_compliance.merge(dummy_coded_df_compliance, on = 'src_subject_id', how= 'left')

#-----------------------------------------------------------------


#------------- SF-12 Survey (health) -------------------------------
# OUTPUT dataframe: df_sf12

df_sf12 = pd.read_excel(dir_input + 'sf1201.xlsx', skiprows=[1])

# Filter the visit, just baseline, and drop the visit column.  
df_sf12 = df_sf12[df_sf12['visit'] == 'B'].drop(columns = 'visit')

sf12_list = ['src_subject_id', 'sf01', 'sf02', 'sf03', 'sf04', 'sf05', 'sf06', 'sf07', 'sf08', 'sf09', 'surm029', 'surm030', 'surm031',
    'physical', 'mental', 'rawpcs12', 'rawmcs12']

#Get selected columns.
df_sf12 = df_sf12[sf12_list].copy()

#-----------------------------------------------------------------


#------------- Vitals --------------------------------------------
# OUTPUT dataframe: df_vitals

df_vitals = pd.read_excel(dir_input + 'vitals01.xlsx', skiprows=[1])

# Filter the visit, just baseline, and drop the visit column.  
df_vitals = df_vitals[df_vitals['visit'] == 'B'].drop(columns = 'visit')

selected_variables = ['src_subject_id', 'vital_sysbp', 'vital_diabp', 'vital_pulse', 'bmi', 'waist', 'height_std', 
                      'weight_std', 'vtl004a', 'vtl004b', 'vtl004c', 'arith_temperature_ct', 'fatmass', 'fatpct']

#Select specific columns
df_vitals = df_vitals[selected_variables].copy()

df_vitals

#-----------------------------------------------------------------


#------------- Lab blood tests -----------------------------------
# OUTPUT dataframe: df_lab

df_lab = pd.read_excel(dir_input + 'clinlabtests01.xlsx', skiprows=[1])

# Filter the visit, just baseline, and drop the visit column.  
df_lab = df_lab[df_lab['visit'] == 'B'].drop(columns = 'visit')

selected_variables = ["src_subject_id", "ls_sodium", "ls_potassium", "ls_chloride", "ls_co2", "ls_glucose", 
         "ls_creatinine", "ls_ureanitrogen", "ls_totprotein", "ls_albumin", "ls_bilirubin", "ls_alkaline", 
         "ls_ast", "ls_alt", "ls_calcium", "rsptc_no", "rsphdl_no", "rspldl_no", "rsptrig_no", "laba4", 
         "laba9a", "ch_ratio", "anion"]

#Select specific columns
df_lab = df_lab[selected_variables].copy()

#-----------------------------------------------------------------


#------------- Medical history -----------------------------------
# OUTPUT dataframe: df_medhist

df_medhist = pd.read_excel(dir_input + 'coinsmedhistory01.xlsx', skiprows=[1])

# Filter the visit, just baseline, and drop the visit column.  
df_medhist = df_medhist[df_medhist['visit'] == 'B'].drop(columns = 'visit')

selected_variables_1 = ["midihist_001", "midihist_002", "midihist_004", "midihist_006", "midihist_007", "midihist_008", 
         "midihist_009", "midihist_010", "midihist_011", "midihist_012", "midihist_013", "midihist_014", 
         "midihist_015", "midihist_016", "midihist_017", "midihist_018", "midihist_019", "midihist_020", 
         "mhendoce", "midihist_025", "midihist_026", "midihist_036", "midihist_040", "mhx026a", "mhx002a", "mhxa1e", "psqb02"]

#Remove 9 -> np.nan from the first selection of variables.
for variable in selected_variables_1:
    df_medhist.loc[df_medhist[variable] == 9, variable] == np.nan

#Change Yes - > 1 and No -> 0 for second selection of variables.
selected_variable_2 = "rev_endodiabetes"
df_medhist.loc[df_medhist[selected_variable_2] == 'Yes' ,selected_variable_2] = 1
df_medhist.loc[df_medhist[selected_variable_2] == 'No' ,selected_variable_2] = 0

selected_variables_3 = ["mhx028d", "bima_28", "bima_29", "bima_30", "bima_31"]

selected_variables = ['src_subject_id'] + selected_variables_1 + [selected_variable_2] + selected_variables_3

df_medhist = df_medhist[selected_variables].copy()
#-----------------------------------------------------------------

#------------- Substance use  -----------------------------------
# OUTPUT dataframe: df_subuse

df_subuse = pd.read_excel(dir_input + 'subusmf01.xlsx', skiprows=[1])

# Filter the visit, just baseline, and drop the visit column.  
df_subuse = df_subuse[df_subuse['visit'] == 'B'].drop(columns = 'visit')

selected_variables = ['src_subject_id', 'subus25', 'subus26', 'subus27', 'ca824', 'subus29', 'subus30', 'scid21', 'subus33', 'subtr4', 'night', 'subtr50', 'outvisit', 'subtr50b', 'subus66', 'surq_1e', 'surq_3a', 'frquency_other', 'subus70']

#subus 29 only filled when ca824 is 0. Add them to also have previous use as current + previous use.
df_subuse.loc[df_subuse['ca824'] == 1, 'subus29'] = 1


df_subuse = df_subuse[selected_variables].copy()

#-----------------------------------------------------------------


#------------- Movement side eff.  -------------------------------
# OUTPUT dataframe: df_move
df_move = pd.read_excel(dir_input + 'aims01.xlsx', skiprows=[1])

# Filter the visit, just baseline, and drop the visit column.  
df_move = df_move[df_move['visit'] == 'B'].drop(columns = 'visit')

selected_variables = ["src_subject_id", "aims_facial_score_date1", "aims_extrem_score_date1", "aims_trunk_score_date1", "aims_global8_date1", "eps1", "eps2", "eps3", "eps4", "eps5", "epsglob", "epsmean"]

df_move = df_move[selected_variables].copy()

#Remove 9 - >np.nan
remove_list = ["eps1", "eps2", "eps3", "eps4", "eps5"]
for variable in remove_list:
    df_move.loc[df_move[variable] == 9, variable] == np.nan

#-----------------------------------------------------------------

#------------- Side effects scale.  -------------------------------
# OUTPUT dataframe: df_sideff
df_sideff = pd.read_excel(dir_input + 'prise01.xlsx', skiprows=[1])

# Filter the visit, just baseline, and drop the visit column.  
df_sideff = df_sideff[df_sideff['visit'] == 'B'].drop(columns = 'visit')

#Select relevant variables. 
selected_variables = ["src_subject_id", "gcnst", "gdmth", "gnsea", "nvdzy", "eyvsn", "urmns", "sldif", "slmch", "sxls", "orsls", "oftge", "saldr", "appf", "weig", "weil", "shak", "stiff", "drodd", "osex", "pbrea", "imsp", "sedation", "eps", "anticholse", "incappwt", "probwsex"]
df_sideff = df_sideff[selected_variables].copy()

#-----------------------------------------------------------------


#------------- Well being scale  ---------------------------------
# OUTPUT dataframe: df_wellbeing
df_wellbeing = pd.read_excel(dir_input + 'pwb01.xlsx', skiprows=[1])

# Filter the visit, just baseline, and drop the visit column.  
df_wellbeing = df_wellbeing[df_wellbeing['visit'] == 'B'].drop(columns = 'visit')

#Select columns.
src_subject_id = ['src_subject_id', 'pwb_3', 'pwb_6', 'pwb_8', 'pwb_13', 'pwb_14', 'pwb_21', 'pwb_29', 'pwb_34', 'pwb_36', 'pwb_41', 'pwb_43', 'pwb_47', 'pwb_51', 'pwb_55', 'pwb_64', 'pwb_72', 'pwb_76', 'pwb_80', 'pw_autonomy', 'pw_envmast', 'pw_persgrowth', 'pw_posrel', 'pw_purp', 'pw_selfa', 'wbtotx']
df_wellbeing = df_wellbeing[src_subject_id].copy()

#-----------------------------------------------------------------

#------------- Mental health recovery measure  -------------------
# OUTPUT dataframe: df_mhrm
df_mhrm = pd.read_excel(dir_input + 'mhrm01.xlsx', skiprows=[1])


# Filter the visit, just baseline, and drop the visit column.  
df_mhrm = df_mhrm[df_mhrm['visit'] == 'B'].drop(columns = 'visit')

selected_variables = ['src_subject_id', 'srf019', 'srf020', 'srf021', 'srf022', 'srf023', 'srf024', 'srf025', 'srf026',
                      'srf027', 'srf028', 'srf029', 'srf030', 'srf031', 'srf032', 'srf033']

# Select variables.
df_mhrm = df_mhrm[selected_variables].copy()

#remove entries where all selected variables are nan. 
df_mhrm.dropna(subset=['srf019', 'srf020', 'srf021', 'srf022', 'srf023', 'srf024', 'srf025', 'srf026','srf027', 
                       'srf028', 'srf029', 'srf030', 'srf031', 'srf032', 'srf033'], how='all', inplace=True)

#-----------------------------------------------------------------

#------------- Autonomy support scale -------- -------------------
# OUTPUT dataframe: df_autsup
df_autsup = pd.read_excel(dir_input + 'autsup01.xlsx', skiprows=[1]).filter(regex  = "^(src_subject_id$|srf|autsupx|visit)")

# Filter the visit, just baseline, and drop the visit column.  
df_autsup = df_autsup[df_autsup['visit'] == 'B'].drop(columns = 'visit')

#-----------------------------------------------------------------


#------------- Medication influences and beliefs ----------------
# OUTPUT dataframe: df_bemib
df_bemib = pd.read_excel(dir_input + 'bam01.xlsx', skiprows=[1]).filter(regex  = "^(src_subject_id$|srf|bemibx|visit)")

# Filter the visit, just baseline, and drop the visit column.  
df_bemib = df_bemib[df_bemib['visit'] == 'B'].drop(columns = 'visit')

#-----------------------------------------------------------------


#------------- SRSS Survey (stigma) ------------------------------
# OUTPUT dataframe: df_srss

df_srss = pd.read_excel(dir_input + 'srss01.xlsx', skiprows=[1]).filter(regex="^(src_subject_id$|srf|stigmax|visit$)")

# Filter the visit, just baseline, and drop the visit column.  
df_srss = df_srss[df_srss['visit'] == 'B'].drop(columns = 'visit')

#-----------------------------------------------------------------

#------------- Patient self rated globals   -------------------
# OUTPUT dataframe: df_selfglob
# IMPORTANT: this data was appended at the end of 'mhrm01.xlsx', which is the mental health recovery data. 

df_selfglob = pd.read_excel(dir_input + 'mhrm01.xlsx', skiprows=[1])


# Filter the visit, just baseline, and drop the visit column.  
df_selfglob = df_selfglob[df_selfglob['visit'] == 'B'].drop(columns = 'visit')

selected_variables = ['src_subject_id', 'srf072', 'srf073']

# Select variables.
df_selfglob = df_selfglob[selected_variables].copy()

#remove entries where all selected variables are nan. IMPORTANT: this dataset is a merge of 
df_selfglob.dropna(subset=['srf072', 'srf073'], how='all', inplace=True)

df_selfglob
#-----------------------------------------------------------------

#------------- Neurocognitive assessment (BACS) ------------------
# OUTPUT dataframe: df_neurocog
df_neurocog = pd.read_excel(dir_input + 'bac01.xlsx', skiprows=[1])

# Filter the visit, just baseline, and drop the visit column.  
df_neurocog = df_neurocog[df_neurocog['visit'] == 'B'].drop(columns = 'visit')


selected_variables = ["src_subject_id", "bacs_dstotalscore", "bacs_sc_total", "bacs_tl_total", "bacs_vmttot", "bacs_fltot", "bacvm1", "bacvm2", "bacvm3",
           "bacvm4", "bacvm5", "bactmts", "bacflua", "bacfluf", "bacflus"]

df_neurocog = df_neurocog[selected_variables].copy()

#Rows are repeated, so we group by. The max does not matter since the numbers are repeated. 
df_neurocog = df_neurocog.groupby('src_subject_id').agg('max').reset_index()
#-----------------------------------------------------------------

#------------- Family assessment scale --------------------------
# OUTPUT dataframe: df_famburd

df_famburd = pd.read_excel(dir_input + 'famburd01.xlsx', skiprows=[1]).filter(regex="^(src_subject_id$|fas|f1d|f2p|f3g|f4t|f5w|fbur|visit$)")

# Filter the visit, just baseline, and drop the visit column.  
df_famburd = df_famburd[df_famburd['visit'] == 'B'].drop(columns = 'visit')

# Replace values of 9 with np.nan in columns starting with 'fas'
fas_columns = [col for col in df_famburd.columns if col.startswith('fas')]
df_famburd[fas_columns] = df_famburd[fas_columns].replace(9, np.nan)

#-----------------------------------------------------------------

#------------- Recovery outcomes assessment ------------------------
# OUTPUT dataframe: df_famrecov

df_famrecov = pd.read_excel(dir_input + 'famrecov01.xlsx', skiprows=[1]).filter(regex="^(src_subject_id$|roa004|roa005|roa006|roa007|roa008|roa010|roa011|roa012|roa013|spous|roa015|roa016|visit$)")

# Filter the visit, just baseline, and drop the visit column.  
df_famrecov = df_famrecov[df_famrecov['visit'] == 'B'].drop(columns = 'visit')

# replace 99 and 9 with nan.
roa_columns = [col for col in df_famburd.columns if (col.startswith('roa') or col.startswith('spous'))]
df_famrecov[roa_columns] = df_famrecov[roa_columns].replace(9, np.nan)
df_famrecov[roa_columns] = df_famrecov[roa_columns].replace(99, np.nan)

#Dummy coding.
dummy_coded = pd.get_dummies(df_famrecov['roa004'], prefix = 'roa004')
df_famrecov.drop(columns = 'roa004', inplace = True)
df_famrecov = pd.concat([df_famrecov, dummy_coded], axis = 1)

#-----------------------------------------------------------------


#------------- Demographics, diagnoses, and other participant descriptors ------------------------
# OUTPUT dataframe: df_demo, df_covars

#Read the covars: site. 
df_covars = pd.read_excel(dir_input + 'ptchart01.xlsx', skiprows=[1], nrows=404).filter(regex="^(src_subject_id$|site$|visit)")

# Filter the visit, just baseline, and drop the visit column.  
df_covars = df_covars[df_covars['visit'] == 'B'].drop(columns = 'visit')

# Read the childcare variables. IMPORTANT: this are variables merged at the end of the raw file which are empty in the rows where df_demo is not empty. 
df_childcare = pd.read_excel(dir_input + 'ptchart01.xlsx', skiprows=[1]).filter(regex="^(src_subject_id$|chldyn$|numchild$|children_household$|chldresp$|chldlvyn$|visit)")

# Filter the visit, just baseline, and drop the visit column.  
df_childcare = df_childcare[df_childcare['visit'] == 'B'].drop(columns = 'visit')

# Remove rows where all selected variables are NaN
df_childcare = df_childcare.dropna(subset=['chldyn', 'numchild', 'children_household', 'chldresp', 'chldlvyn'], how='all')
df_childcare = df_childcare[['src_subject_id', 'chldyn', 'numchild', 'children_household', 'chldresp', 'chldlvyn']].copy()

#Dummy code chldlvyn
dummy_coded = pd.get_dummies(df_childcare['chldlvyn'], prefix = 'chldlvyn')
df_childcare.drop(columns = 'chldlvyn', inplace = True)
df_childcare = pd.concat([df_childcare, dummy_coded], axis = 1)


df_demo = pd.read_excel(dir_input + 'ptchart01.xlsx', skiprows=[1], nrows=404)

# Filter the visit, just baseline, and drop the visit column.  
df_demo = df_demo[df_demo['visit'] == 'B'].drop(columns = 'visit')

#Variables that need converting into binary. 
selected_variable_00 = 'sex'
selected_variable_01 = 'treatgrp'
selected_variable_02 = 'refothers'
selected_variable_03 = 'ethnicity'

#Converting sex into binary.
df_demo.loc[df_demo[selected_variable_00] == 'F', selected_variable_00] = 2
df_demo.loc[df_demo[selected_variable_00] == 'M', selected_variable_00] = 1

#Converting treatment into binary.
df_demo.loc[df_demo[selected_variable_01] == 'N', selected_variable_01] = 2
df_demo.loc[df_demo[selected_variable_01] == 'CC', selected_variable_01] = 1

#Converting referal source into binary.
df_demo.loc[df_demo[selected_variable_02] == 'In treatment at the site or usual referral source', selected_variable_02] = 2
df_demo.loc[df_demo[selected_variable_02] == 'Community outreach', selected_variable_02] = 1

#Converting ethnicity source into binary.
df_demo.loc[df_demo[selected_variable_03] == 'Not Hispanic or Latino', selected_variable_03] = 2
df_demo.loc[df_demo[selected_variable_03] == 'Hispanic or Latino', selected_variable_03] = 1

#Variables that do not need modification.
selected_variables_1 = ['src_subject_id', 'sex', 'treatgrp', 'refothers', 'ethnicity', 'dup', 'interview_age', 'studntyn', 'workyn', 'job1yn', 'job1age', 
             'longest', 'lnghrs', 'job_pthi', 'job_ptcu', 'dem_09a', 'dem_09b', 'ageill', 'hpl_02', 'psyhspyn', 'age_1st_hosp', 'numhosp1', 'tdayshosp', 'wkslsthp',
             'wksset', 'lfapcon', 'lfapbl', 'curapbl', 'sgafgayn', 'psych_subonset']

#Variables that need np.nan -> 0 and dummy coding. 
selected_variables_2 = ['alcoh30', 'sedat30', 'cannab30', 'stimu30', 'opioid30', 'coca30', 'pcp30', 'poly30', 'other30']

for var in selected_variables_2:
    df_demo.loc[df_demo[var] == np.nan, var] = 0


#Variables that need dummy coding.
selected_variables_3 = ['race', 'das1ms', 'curres', 'educpt', 'educmo', 
                        'educfa', 'trt_set', 'dxbase', 'alcohlf', 'sedatlf', 'cannablf', 'stimulf', 'opioidlf', 
                        'cocalf', 'pcplf', 'polylf', 'otherlf']

#Get all relevant variables
selected_variables = selected_variables_1 + selected_variables_2 + selected_variables_3
df_demo = df_demo[selected_variables].copy()

#Remove 99 -> np.nan
for var in selected_variables:
    df_demo.loc[df_demo[var] == 99, var] = np.nan

#Dummy code the variables.
for var in selected_variables_2 + selected_variables_3:
    #Dummy code the variable and replace with dummy coding.
    dummy_coded = pd.get_dummies(df_demo[var], prefix = var)
    df_demo.drop(columns = var, inplace = True)
    df_demo = pd.concat([df_demo, dummy_coded], axis = 1)

#Merge df_demo and df_childcare on src_subject_id
df_demo = df_demo.merge(df_childcare, on='src_subject_id', how = 'left')
#-------------------------------------------------------------------------------------------------

#------------- Smoking instrument ---------------------------------
# OUTPUT dataframe: df_fager

df_fager = pd.read_excel(dir_input + 'fagerstrom01.xlsx', skiprows=[1])

# Filter the visit, just baseline, and drop the visit column.  
df_fager = df_fager[df_fager['visit'] == 'B'].drop(columns = 'visit')

selected_variables = ['src_subject_id', 'ftnd_7', 'q01_first_cig', 'q02_forbidden', 'q03_give_up', 'q04_per_day', 
                      'q05_frequency', 'q06_ill', 'ftnd_score_total', 'mhx052', 'mhx053', 'mhx054', 
                      'mhx055', 'mhx056', 'mhx057', 'mhx058', 'chwscore']

#Select the variables.
df_fager = df_fager[selected_variables].copy()


#-------------------------------------------------------------------

#------------- Service utilisation (monhly) ------------------------
# OUTPUT dataframe: df_servm
# IMPORTANT: variables included in this dataset have very specific questions. 
#It is not clear how these might be helpful in ML analyses.
df_servm = pd.read_excel(dir_input + 'surf01.xlsx', skiprows=[1])

# Filter the visit, just baseline, and drop the visit column.  
df_servm = df_servm[df_servm['visit'] == 'B'].drop(columns = 'visit')

selected_variables = ['src_subject_id', 'inpat1a', 'inpat1b', 'inpat1c', 'inpat1d', 'surfm31', 'night2', 'surfm41', 'night3', 
         'srfa5a1', 'srfa5a2', 'srfa5a3', 'srfa5c1', 'srfa5c2', 'srfa5c3', 'srfa5d1', 'srfa5d2', 
         'srfa5d3', 'srfa5e1', 'srfa5e2', 'srfa5e3', 'srfa5f1', 'srfa5f2', 'srfa5f3', 'srfa5g1', 
         'srfa5g2', 'srfa5g3', 'srfa5h1', 'srfa5j1', 'srfa5j2', 'srfa5j3', 'srfa5k1', 'srfa5k2', 
         'srfa5k3', 'srfa5l1', 'srfa5l2', 'srfa5l3', 'srfa5o1', 'srfa5o2', 'srfa5o3', 'srfa6a', 
         'srfa6b', 'srfa6c', 'srfa6d', 'srfa6e', 'srfa6f', 'srfa6g', 'srfa6h', 'srf1', 'srf2', 
         'srf3', 'srf4', 'surm007', 'surm007b', 'surm008', 'surm008b', 'surm011', 
         'surm013', 'dworked', 'hworked', 'earnweek', 'surfq2', 'surfq3', 'surfq3a', 'surfq3b', 
         'surm019', 'surm041', 'surm041a', 'surm041b', 'surm042', 'surm042a', 'surm042b', 'surm044c', 
         'surm045', 'outpprof', 'surm045b', 'surm046', 'surm046a', 'surm046b', 'surm048c', 'surm049', 
         'surm049a', 'surm049b', 'surm052', 'surm052a', 'surm059', 'surm060', 'surm061', 'surm062', 
         'surm063', 'surm064', 'surm065', 'surm071', 'surm071a', 'surm072', 'surm072a', 'surm073', 
         'surm073a', 'surm074', 'surm074a', 'wrkfrpay', 'surm009', 'surm012', 'surfq2a', 'surm043']
#Get only selected variables.
df_servm = df_servm[selected_variables].copy()

#Dummy code the categorical variables.
for var in ['surm009', 'surm012', 'surm043']:
    #Dummy code the variable and replace with dummy coding.
    dummy_coded = pd.get_dummies(df_servm[var], prefix = var)
    df_servm.drop(columns = var, inplace = True)
    df_servm = pd.concat([df_servm, dummy_coded], axis = 1)

#-----------------------------------------------------------------


#------------- Service utilisation (quarter) ------------------------
# OUTPUT dataframe: df_servq
# IMPORTANT: variables included in this dataset have very specific questions. 
#It is not clear how these might be helpful in ML analyses.

df_servq = pd.read_excel(dir_input + 'surfq01.xlsx', skiprows=[1])


# Filter the visit, just baseline, and drop the visit column.  
df_servq = df_servq[df_servq['visit'] == 'B'].drop(columns = 'visit')

# Variables to extract.
selected_variables = ['src_subject_id', 'surfqa', 'surfqaa', 'surfqb', 'surfqba', 'surfqc', 'surfqca', 'surfqd', 'surfqda', 'surfqe', 'surfqea', 
         'surfqh', 'surfqha', 'surfqi', 'surfqia', 'surfqk', 'surfqka', 'surfql', 'surfqla', 'surfqm', 'priv1a', 
         'publica', 'medcaid', 'insur2g', 'surq018', 'surq019', 'surq020', 'surq021', 'surq026', 'surq027', 
         'instype', 'arrest', 'police', 'court', 'nitejail', 'parole', 'officer', 'timeoff', 'surq011', 'surq012']

df_servq = df_servq[selected_variables].copy()

# Variables to convert 3 -> np.nan
selected_variables_2 =  ['surfqa', 'surfqb', 'surfqc', 'surfqd', 'surfqe', 'surfqh', 'surfqi', 'surfqk', 'surfql', 
                       'surfqm', 'priv1a', 'publica', 'medcaid']

#Convert 3 to nan in selected variables.
for var in selected_variables_2:
    df_servq.loc[df_servq[var] == 3,var] = np.nan

#Dummy code the variable and replace with dummy coding.
dummy_coded = pd.get_dummies(df_servq['insur2g'], prefix = var)
df_servq.drop(columns = 'insur2g', inplace = True)
df_servq = pd.concat([df_servq, dummy_coded], axis = 1)

#-----------------------------------------------------------------


#-------------- Medication at consent ----------------------------
# OUTPUT dataframe: df_medcon
df_medcon_v1 = pd.read_excel(dir_input + 'medcon01.xlsx', skiprows=[1])

# Filter the visit, just baseline, and drop the visit column.  
df_medcon_v1 = df_medcon_v1[df_medcon_v1['visit'] == 'B'].drop(columns = 'visit')

selected_variables = ['src_subject_id', 'adhetpc1']

df_medcon_v1 = df_medcon_v1[selected_variables].copy()

# Dummy code the drug.
dummy_coded = pd.get_dummies(df_medcon_v1['adhetpc1'], prefix = 'adhetpc1')
df_medcon_v1.drop(columns = 'adhetpc1', inplace = True)
df_medcon_v1 = pd.concat([df_medcon_v1, dummy_coded], axis = 1)

#Agreggate all columns. 
df_medcon = df_medcon_v1.groupby('src_subject_id').agg('max').reset_index()

#-----------------------------------------------------------------

#-------------- Medication adherence -----------------------------
# OUTPUT dataframe: df_medadh

df_medadh = pd.read_excel(dir_input + 'adh01.xlsx', skiprows=[1])

# Filter the visit, just baseline, and drop the visit column.  
df_medadh = df_medadh[df_medadh['visit'] == 'B'].drop(columns = 'visit')

selected_variables = ['src_subject_id', "nbrmeds", "nbrpill1", "daysnot1", "daysles1", "nbrpill2", "daysnot2", "daysles2", "nbrpilex", "nbrpilms"]
df_medadh = df_medadh[selected_variables].copy()

#Create a variable of ratio of pills missed. 
df_medadh['nbrpilratio'] = df_medadh['nbrpilms']/df_medadh['nbrpilex']
#-----------------------------------------------------------------
```

In [16]:

```
################################################################################
#Storing Data and Merging
################################################################################
# Sergio Mena Ortega, 2024

#Writing individual datasets into excel.
df_panss.to_excel(dir_output + 'Individual Datasets/panss.xlsx', index=None) #
df_cgi.to_excel(dir_output + 'Individual Datasets/cgi.xlsx', index=None) #
df_cdss.to_excel(dir_output + 'Individual Datasets/cdss.xlsx', index=None) #
df_qol.to_excel(dir_output + 'Individual Datasets/qol.xlsx', index=None) #
df_compliance.to_excel(dir_output + 'Individual Datasets/comp.xlsx', index=None) #
df_sf12.to_excel(dir_output + 'Individual Datasets/sf12.xlsx', index=None) #
df_vitals.to_excel(dir_output + 'Individual Datasets/vitals.xlsx', index=None) #
df_lab.to_excel(dir_output + 'Individual Datasets/lab.xlsx', index=None) #
df_medhist.to_excel(dir_output + 'Individual Datasets/medhist.xlsx', index=None) #
df_subuse.to_excel(dir_output + 'Individual Datasets/subuse.xlsx', index=None) #
df_move.to_excel(dir_output + 'Individual Datasets/move.xlsx', index=None) # 
df_sideff.to_excel(dir_output + 'Individual Datasets/sideff.xlsx', index=None) #
df_wellbeing.to_excel(dir_output + 'Individual Datasets/wellbeing.xlsx', index=None) #
df_mhrm.to_excel(dir_output + 'Individual Datasets/mhrm.xlsx', index=None) #
df_autsup.to_excel(dir_output + 'Individual Datasets/autsup.xlsx', index=None) #
df_bemib.to_excel(dir_output + 'Individual Datasets/bemib.xlsx', index=None) # 
df_srss.to_excel(dir_output + 'Individual Datasets/srss.xlsx', index=None) #
df_selfglob.to_excel(dir_output + 'Individual Datasets/selfglob.xlsx', index=None) #
df_neurocog.to_excel(dir_output + 'Individual Datasets/neurocog.xlsx', index=None) #
df_famburd.to_excel(dir_output + 'Individual Datasets/famburd.xlsx', index=None) #
df_famrecov.to_excel(dir_output + 'Individual Datasets/famrecov.xlsx', index=None) #
df_demo.to_excel(dir_output + 'Individual Datasets/demo.xlsx', index=None) #
df_covars.to_excel(dir_output + 'Individual Datasets/covars.xlsx', index=None) #
df_fager.to_excel(dir_output + 'Individual Datasets/fager.xlsx', index=None) #
df_servm.to_excel(dir_output + 'Individual Datasets/servm.xlsx', index=None) #
df_servq.to_excel(dir_output + 'Individual Datasets/servq.xlsx', index=None) #
df_medcon.to_excel(dir_output + 'Individual Datasets/medcon.xlsx', index=None) #
df_medadh.to_excel(dir_output + 'Individual Datasets/medadh.xlsx', index=None) #

#Merging data into 4 categories: clinical+sociodemographic, cognitive, QoL and lab data.

#Get the original ids. 
df_id =  pd.read_excel(dir_input + 'ptchart01.xlsx', skiprows=[1], nrows=404).filter(regex="^(src_subject_id$|visit$)")

# Filter the visit, just baseline, and drop the visit column.  
df_id = df_id[df_id['visit'] == 'B'].drop(columns = 'visit')


#Clinical and sociodemographic data category.
df_clin_and_socio = df_id.copy()
list_of_dataframes = [df_demo, df_panss, df_cdss, df_cgi, df_famburd, df_famrecov, 
                      df_compliance, df_servm, df_servq, df_fager, df_medcon, df_medadh,
                      df_selfglob, df_srss, df_bemib, df_autsup, df_mhrm, df_sideff, df_move, df_subuse, df_medhist]

for data in list_of_dataframes:
    df_clin_and_socio = pd.merge(df_clin_and_socio, data, on='src_subject_id', how='left')
    
#Save to excel.    
df_clin_and_socio.to_excel(dir_output+'Merged Datasets/clinical_and_sociodemographic.xlsx', index = False)


#Health category. 
df_health = pd.merge(df_id, df_lab, on='src_subject_id', how='left')
df_health = pd.merge(df_health, df_sf12, on='src_subject_id', how='left')
df_health = pd.merge(df_health, df_vitals, on='src_subject_id', how='left')
#Save to excel.    
df_health.to_excel(dir_output+'Merged Datasets/health.xlsx', index = False)


#Cognitive category. 
df_cognitive = pd.merge(df_id, df_neurocog, on='src_subject_id', how='left')
#Save to excel.    
df_cognitive.to_excel(dir_output+'Merged Datasets/cognitive.xlsx', index = False)

##Quality of life category . 
df_quality_of_life = pd.merge(df_id, df_qol, on='src_subject_id', how='left')
df_quality_of_life = pd.merge(df_quality_of_life, df_wellbeing, on='src_subject_id', how='left')
#Save to excel.    
df_quality_of_life.to_excel(dir_output+'Merged Datasets/quality_of_life.xlsx', index = False)

##Covariates (df_covariates).
df_covariates = pd.merge(df_id, df_covars, on='src_subject_id', how='left')
#Save to excel.    
df_covariates.to_excel(dir_output+'Merged Datasets/covariates.xlsx', index = False)
```

In [18]:

```
#-------------- Medication at consent ----------------------------
# OUTPUT dataframe: df_medcon
df_medcon_v1 = pd.read_excel(dir_input + 'medcon01.xlsx', skiprows=[1])

# Filter the visit, just baseline, and drop the visit column.  
df_medcon_v1 = df_medcon_v1[df_medcon_v1['visit'] == 'B'].drop(columns = 'visit')

selected_variables = ['src_subject_id', 'adhetpc1']

df_medcon_v1 = df_medcon_v1[selected_variables].copy()

# Dummy code the drug.
dummy_coded = pd.get_dummies(df_medcon_v1['adhetpc1'], prefix = 'adhetpc1')
df_medcon_v1.drop(columns = 'adhetpc1', inplace = True)
df_medcon_v1 = pd.concat([df_medcon_v1, dummy_coded], axis = 1)

#Agreggate all columns. 
df_medcon = df_medcon_v1.groupby('src_subject_id').agg('max').reset_index()

#-----------------------------------------------------------------
```

Out[18]:

|  | src\_subject\_id | adhetpc1\_1.0 | adhetpc1\_2.0 | adhetpc1\_3.0 | adhetpc1\_4.0 | adhetpc1\_5.0 | adhetpc1\_6.0 | adhetpc1\_7.0 | adhetpc1\_8.0 | adhetpc1\_9.0 | adhetpc1\_11.0 | adhetpc1\_12.0 | adhetpc1\_13.0 | adhetpc1\_14.0 | adhetpc1\_16.0 | adhetpc1\_17.0 | adhetpc1\_19.0 | adhetpc1\_21.0 | adhetpc1\_22.0 | adhetpc1\_23.0 | adhetpc1\_24.0 | adhetpc1\_25.0 | adhetpc1\_30.0 | adhetpc1\_31.0 | adhetpc1\_32.0 | adhetpc1\_34.0 | adhetpc1\_35.0 | adhetpc1\_36.0 | adhetpc1\_37.0 | adhetpc1\_38.0 | adhetpc1\_39.0 | adhetpc1\_41.0 | adhetpc1\_42.0 | adhetpc1\_43.0 | adhetpc1\_44.0 | adhetpc1\_50.0 | adhetpc1\_70.0 | adhetpc1\_80.0 | adhetpc1\_90.0 | adhetpc1\_100.0 | adhetpc1\_101.0 | adhetpc1\_110.0 | adhetpc1\_120.0 | adhetpc1\_140.0 | adhetpc1\_160.0 | adhetpc1\_170.0 | adhetpc1\_180.0 | adhetpc1\_190.0 | adhetpc1\_200.0 | adhetpc1\_205.0 | adhetpc1\_210.0 | adhetpc1\_220.0 |
| --- | --- | --- | --- | --- | --- | --- | --- | --- | --- | --- | --- | --- | --- | --- | --- | --- | --- | --- | --- | --- | --- | --- | --- | --- | --- | --- | --- | --- | --- | --- | --- | --- | --- | --- | --- | --- | --- | --- | --- | --- | --- | --- | --- | --- | --- | --- | --- | --- | --- | --- | --- | --- |
| 0 | 47 | False | False | False | False | False | False | False | False | False | False | False | False | False | False | False | False | False | False | False | False | False | False | False | False | False | False | False | False | False | False | False | False | False | False | True | False | False | False | False | False | False | False | False | False | False | False | False | False | False | False | False |
| 1 | 47 | False | False | False | False | False | False | False | False | False | False | False | False | False | False | False | False | False | False | False | False | False | False | False | False | False | False | False | False | False | False | False | False | False | False | False | True | False | False | False | False | False | False | False | False | False | False | False | False | False | False | False |
| ... | ... | ... | ... | ... | ... | ... | ... | ... | ... | ... | ... | ... | ... | ... | ... | ... | ... | ... | ... | ... | ... | ... | ... | ... | ... | ... | ... | ... | ... | ... | ... | ... | ... | ... | ... | ... | ... | ... | ... | ... | ... | ... | ... | ... | ... | ... | ... | ... | ... | ... | ... | ... |
| 14123 | 560 | False | False | False | False | False | False | False | False | False | False | False | True | False | False | False | False | False | False | False | False | False | False | False | False | False | False | False | False | False | False | False | False | False | False | False | False | False | False | False | False | False | False | False | False | False | False | False | False | False | False | False |
| 14124 | 560 | False | False | False | False | False | False | False | False | False | False | False | False | False | False | False | False | False | False | False | False | False | False | False | False | False | False | False | False | False | False | False | False | False | False | False | False | False | False | True | False | False | False | False | False | False | False | False | False | False | False | False |

1785 rows × 52 columns

In [ ]:

```
df_medcon
```
