# Supplementary material for: AI-based prediction of depression symptomatology in first-episode psychosis patients: insights from the EUFEST and RAISE-ETP clinical trials
Source: Psychol Med. 2025 Jul 30;55:e221. doi: 10.1017/S0033291725100950 (PMC12341035; doi:10.1017/S0033291725100950)
Supplement: Mena et al. supplementary material 2 — Mena et al. supplementary material [file S0033291725100950sup002.zip › supporting files/prediction_set_extraction_eufest.html]

prediction\_set\_extraction


In [30]:

```
import pandas as pd
import numpy as np

pd.set_option('display.max_rows', None)
pd.set_option('display.max_columns', None)

#Folder name with original input data.
dir_input = 'Raw data/'

#Folder name for output data.
dir_output = 'Prediction set/'
```

In [31]:

```
################################################################################
#Data Extraction
################################################################################
# Sergio Mena Ortega, 2024


#------------- Global Assessment of Functioning (GAF) ---------------------------
# OUTPUT dataframe: df_gaf

df_gaf = pd.read_excel(dir_input+f'gaf_070904.xlsx', sheet_name='Data').filter(regex=f'^(crfnr)')
visit_columns = ['V3', 'V5', 'V6', 'V7', 'V8', 'V9']

# Iterate through each visit
for visit in visit_columns:
    # Read the excel file for the specific visit
    df_gaf_v = pd.read_excel(dir_input+f'gaf_070904.xlsx', sheet_name='Data').filter(regex=f'^(V1|{visit}|crfnr)')

    #Define good and bad treatment outcome as: ([GAF] score ≥65 vs GAF <65, respectively). 
    df_gaf_v.loc[df_gaf_v[f'{visit}gaf01'] >= 65, f'{visit}GAF_ABS_CLASS'] = 1
    df_gaf_v.loc[df_gaf_v[f'{visit}gaf01'] < 65, f'{visit}GAF_ABS_CLASS'] = 0
    df_gaf_v.loc[df_gaf_v[f'{visit}gaf01'] == np.nan, f'{visit}GAF_ABS_CLASS'] = np.nan

    #Calculate the absolute change in GAF.
    df_gaf_v[f'{visit}GAF_ABSDIFF'] = (df_gaf_v[f'{visit}gaf01'] - df_gaf_v['V1gaf01'])
    
    #Calculate the percentage of increase of GAF. 
    #Note that GAF can be 0, so to calculate the ratio (and avoid getting infinite), we displace the values 1 position.
    df_gaf_v[f'{visit}GAF_DIFF'] = (df_gaf_v[f'{visit}gaf01'] - df_gaf_v['V1gaf01']) / (100 - 0)
    

    #Define good and bad treatment outcome as:  ([GAF] increase > + 50%)
    df_gaf_v.loc[df_gaf_v[f'{visit}GAF_DIFF'] >= 0.2, f'{visit}GAF_DIFF_CLASS'] = 1
    df_gaf_v.loc[df_gaf_v[f'{visit}GAF_DIFF'] < 0.2, f'{visit}GAF_DIFF_CLASS'] = 0
    df_gaf_v.loc[df_gaf_v[f'{visit}GAF_DIFF'] == np.nan, f'{visit}GAF_DIFF_CLASS'] = np.nan
    
    #Add data to df_gaf.
    df_gaf = pd.concat([df_gaf, df_gaf_v[[f'{visit}gaf01', f'{visit}GAF_ABS_CLASS', f'{visit}GAF_ABSDIFF', f'{visit}GAF_DIFF', f'{visit}GAF_DIFF_CLASS']]], axis = 1)

# ------------ PANSS -----------------------------------------------------------
# OUTPUT dataframe: df_panss
df_panss = pd.read_excel(dir_input+f'pansa_070727.xlsx', sheet_name='Data').filter(regex=f'^(crfnr)')

visit_columns = ['V3' ,'V6', 'V7', 'V8', 'V9']

# Iterate through each visit
for visit in visit_columns:
    # Read the excel file for the specific visit
    df_panss_v = pd.read_excel(dir_input+f'pansa_070727.xlsx', sheet_name='Data').filter(regex=f'^(V1|{visit}|crfnr)')
    
    # List of PANSS score columns for visit 1.
    positive_columns_v1 = ['V1pa01', 'V1pa02', 'V1pa03', 'V1pa04', 'V1pa05', 'V1pa06', 'V1pa07']
    negative_columns_v1 = ['V1pa08', 'V1pa09', 'V1pa10', 'V1pa11', 'V1pa12', 'V1pa13', 'V1pa14']
    general_columns_v1 = ['V1pa15', 'V1pa16', 'V1pa17', 'V1pa18', 'V1pa19', 'V1pa20', 'V1pa21', 'V1pa22', 'V1pa23', 'V1pa24', 'V1pa25', 'V1pa26', 'V1pa27', 'V1pa28', 'V1pa29', 'V1pa30']
    # Add new columns for the sum of positive, negative, and general scores for visit 1. 
    df_panss_v['V1ptotal'] = df_panss_v[positive_columns_v1].sum(axis=1)
    df_panss_v['V1ntotal'] = df_panss_v[negative_columns_v1].sum(axis=1)
    df_panss_v['V1gtotal'] = df_panss_v[general_columns_v1].sum(axis=1)

    # Set the sum values to NaN if all individual values are NaN
    df_panss_v.loc[df_panss_v[positive_columns_v1].isna().all(axis=1), 'V1ptotal'] = np.nan
    df_panss_v.loc[df_panss_v[negative_columns_v1].isna().all(axis=1), 'V1ntotal'] = np.nan
    df_panss_v.loc[df_panss_v[general_columns_v1].isna().all(axis=1), 'V1gtotal'] = np.nan

    #Calculate total score. 
    df_panss_v['V1total'] = df_panss_v[['V1ptotal', 'V1ntotal', 'V1gtotal']].sum(axis = 1)

    # Set the total score to NaN if all individual values are NaN
    df_panss_v.loc[df_panss_v[['V1ptotal', 'V1ntotal', 'V1gtotal']].isna().all(axis=1), 'V1total'] = np.nan
    
    
    
    # List of PANSS score columns for the current visit
    positive_columns = [f'{visit}pa01', f'{visit}pa02', f'{visit}pa03', f'{visit}pa04',
                        f'{visit}pa05', f'{visit}pa06', f'{visit}pa07']
    negative_columns = [f'{visit}pa08', f'{visit}pa09', f'{visit}pa10', f'{visit}pa11',
                        f'{visit}pa12', f'{visit}pa13', f'{visit}pa14']
    general_columns = [f'{visit}pa15', f'{visit}pa16', f'{visit}pa17', f'{visit}pa18',
                       f'{visit}pa19', f'{visit}pa20', f'{visit}pa21', f'{visit}pa22',
                       f'{visit}pa23', f'{visit}pa24', f'{visit}pa25', f'{visit}pa26',
                       f'{visit}pa27', f'{visit}pa28', f'{visit}pa29', f'{visit}pa30']

    # Add new columns for the sum of positive, negative, and general scores for the current visit
    df_panss_v[f'{visit}ptotal'] = df_panss_v[positive_columns].sum(axis=1)
    df_panss_v[f'{visit}ntotal'] = df_panss_v[negative_columns].sum(axis=1)
    df_panss_v[f'{visit}gtotal'] = df_panss_v[general_columns].sum(axis=1)

    # Set the sum values to NaN if all individual values are NaN
    df_panss_v.loc[df_panss_v[positive_columns].isna().all(axis=1), f'{visit}ptotal'] = np.nan
    df_panss_v.loc[df_panss_v[negative_columns].isna().all(axis=1), f'{visit}ntotal'] = np.nan
    df_panss_v.loc[df_panss_v[general_columns].isna().all(axis=1), f'{visit}gtotal'] = np.nan

    # Calculate total score
    df_panss_v[f'{visit}total'] = df_panss_v[[f'{visit}ptotal', f'{visit}ntotal', f'{visit}gtotal']].sum(axis=1)

    # Set the total score to NaN if all individual values are NaN
    df_panss_v.loc[df_panss_v[[f'{visit}ptotal', f'{visit}ntotal', f'{visit}gtotal']].isna().all(axis=1), f'{visit}total'] = np.nan

    # Add a new column with 1's and 0's based on Andreasen criteria
    andresen_columns = [f'{visit}pa01', f'{visit}pa23', f'{visit}pa03', f'{visit}pa02',
                        f'{visit}pa19', f'{visit}pa08', f'{visit}pa11', f'{visit}pa13']

    # Add column if meeting ANDREASEN criteria of remission
    df_panss_v[f'{visit}ANDREASEN_ABS_CLASS'] = (df_panss_v[andresen_columns] <= 3).all(axis=1).astype(int)

    # Set the value to NaN if either of Andreasen columns are nan
    df_panss_v.loc[df_panss_v[andresen_columns].isna().any(axis=1), f'{visit}ANDREASEN_ABS_CLASS'] = np.nan

    
    # Absolute change of total sum symptoms
    df_panss_v[f'{visit}PANSS_ABSDIFF'] = (df_panss_v[f'{visit}total'] - df_panss_v['V1total'])
    
    # Percentage change of total sum symptoms
    #Note this is the Percentage of Maximum Possible Change, divided by the total possible change of the total PANSS score. 
    df_panss_v[f'{visit}PANSS_DIFF'] = (df_panss_v[f'{visit}total'] - df_panss_v['V1total']) / (210 - 30)
    
    # Define good and bad treatment outcome as: ([PANSS total] decrease > + 20%)
    df_panss_v.loc[df_panss_v[f'{visit}PANSS_DIFF'] <= -0.2, f'{visit}PANSS_DIFF_CLASS20'] = 1
    df_panss_v.loc[df_panss_v[f'{visit}PANSS_DIFF'] > -0.2, f'{visit}PANSS_DIFF_CLASS20'] = 0
    df_panss_v.loc[df_panss_v[f'{visit}PANSS_DIFF'] == np.nan, f'{visit}PANSS_DIFF_CLASS20'] = np.nan

    # Define good and bad treatment outcome as: ([PANSS total] decrease > + 20%)
    df_panss_v.loc[df_panss_v[f'{visit}PANSS_DIFF'] < 0, f'{visit}PANSS_DIFF_CLASS'] = 1
    df_panss_v.loc[df_panss_v[f'{visit}PANSS_DIFF'] >= 0, f'{visit}PANSS_DIFF_CLASS'] = 0
    df_panss_v.loc[df_panss_v[f'{visit}PANSS_DIFF'] == np.nan, f'{visit}PANSS_DIFF_CLASS'] = np.nan

    # Absolute change of total sum of positive symptoms
    df_panss_v[f'{visit}PANSS_POS_ABSDIFF'] = (df_panss_v[f'{visit}ptotal'] - df_panss_v['V1ptotal'])

    # Percentage change of total sum of positive symptoms
    #Note this is the Percentage of Maximum Possible Change, divided by the total possible change of the total PANSS score. 
    df_panss_v[f'{visit}PANSS_POS_DIFF'] = (df_panss_v[f'{visit}ptotal'] - df_panss_v['V1ptotal']) / (49 - 7)
    
    # Define good and bad treatment outcome for positive symptoms as: ([PANSS positive] increase > + 20%)
    df_panss_v.loc[df_panss_v[f'{visit}PANSS_POS_DIFF'] <= -0.2, f'{visit}PANSS_POS_DIFF_CLASS20'] = 1
    df_panss_v.loc[df_panss_v[f'{visit}PANSS_POS_DIFF'] > -0.2, f'{visit}PANSS_POS_DIFF_CLASS20'] = 0
    df_panss_v.loc[df_panss_v[f'{visit}PANSS_POS_DIFF'].isnull(), f'{visit}PANSS_POS_DIFF_CLASS20'] = np.nan

    # Define good and bad treatment outcome for positive symptoms as: (increase or decrease)
    df_panss_v.loc[df_panss_v[f'{visit}PANSS_POS_DIFF'] < 0, f'{visit}PANSS_POS_DIFF_CLASS'] = 1
    df_panss_v.loc[df_panss_v[f'{visit}PANSS_POS_DIFF'] >= 0, f'{visit}PANSS_POS_DIFF_CLASS'] = 0
    df_panss_v.loc[df_panss_v[f'{visit}PANSS_POS_DIFF']== np.nan, f'{visit}PANSS_POS_DIFF_CLASS'] = np.nan


    # Absolute change of total sum of negative symptoms
    df_panss_v[f'{visit}PANSS_NEG_ABSDIFF'] = (df_panss_v[f'{visit}ntotal'] - df_panss_v['V1ntotal'])
    
    # Percentage change of total sum of negative symptoms
    df_panss_v[f'{visit}PANSS_NEG_DIFF'] = (df_panss_v[f'{visit}ntotal'] - df_panss_v['V1ntotal']) / (49 - 7)
    
    # Define good and bad treatment outcome for negative symptoms as: ([PANSS negative] increase > + 20%)
    df_panss_v.loc[df_panss_v[f'{visit}PANSS_NEG_DIFF'] <= -0.2, f'{visit}PANSS_NEG_DIFF_CLASS20'] = 1
    df_panss_v.loc[df_panss_v[f'{visit}PANSS_NEG_DIFF'] > -0.2, f'{visit}PANSS_NEG_DIFF_CLASS20'] = 0
    df_panss_v.loc[df_panss_v[f'{visit}PANSS_NEG_DIFF'].isnull(), f'{visit}PANSS_NEG_DIFF_CLASS20'] = np.nan

    # Define good and bad treatment outcome for negative symptoms as: ([PANSS negative] increase > + 20%)
    df_panss_v.loc[df_panss_v[f'{visit}PANSS_NEG_DIFF'] < 0, f'{visit}PANSS_NEG_DIFF_CLASS'] = 1
    df_panss_v.loc[df_panss_v[f'{visit}PANSS_NEG_DIFF'] >= 0, f'{visit}PANSS_NEG_DIFF_CLASS'] = 0
    df_panss_v.loc[df_panss_v[f'{visit}PANSS_NEG_DIFF'] == np.nan, f'{visit}PANSS_NEG_DIFF_CLASS'] = np.nan

    # Create a new column that checks if all negative symptoms are ≤ 3
    df_panss_v[f'{visit}PANSS_NEG_REMISSION_ANDREASEN'] = (df_panss_v[negative_columns] <= 3).all(axis=1).astype(int)

    # Set the value to NaN if any of the negative columns are NaN
    df_panss_v.loc[df_panss_v[negative_columns].isna().any(axis=1), f'{visit}PANSS_NEG_REMISSION_ANDREASEN'] = np.nan

    # Absolute change of total sum of general symptoms
    df_panss_v[f'{visit}PANSS_GEN_ABSDIFF'] = (df_panss_v[f'{visit}gtotal'] - df_panss_v['V1gtotal'])
    
    # Percentage change of total sum of negative symptoms
    df_panss_v[f'{visit}PANSS_GEN_DIFF'] = (df_panss_v[f'{visit}gtotal'] - df_panss_v['V1gtotal']) / (112 - 16)

    # Define good and bad treatment outcome for general symptoms as: ([PANSS general] increase > + 20%)
    df_panss_v.loc[df_panss_v[f'{visit}PANSS_GEN_DIFF'] <= -0.2, f'{visit}PANSS_GEN_DIFF_CLASS20'] = 1
    df_panss_v.loc[df_panss_v[f'{visit}PANSS_GEN_DIFF'] > -0.2, f'{visit}PANSS_GEN_DIFF_CLASS20'] = 0
    df_panss_v.loc[df_panss_v[f'{visit}PANSS_GEN_DIFF'].isnull(), f'{visit}PANSS_GEN_DIFF_CLASS20'] = np.nan

        # Define good and bad treatment outcome for general symptoms (increase or decrease)
    df_panss_v.loc[df_panss_v[f'{visit}PANSS_GEN_DIFF'] < 0, f'{visit}PANSS_GEN_DIFF_CLASS'] = 1
    df_panss_v.loc[df_panss_v[f'{visit}PANSS_GEN_DIFF'] >= 0, f'{visit}PANSS_GEN_DIFF_CLASS'] = 0
    df_panss_v.loc[df_panss_v[f'{visit}PANSS_GEN_DIFF'] == np.nan, f'{visit}PANSS_GEN_DIFF_CLASS'] = np.nan
    
    
    # Keep only final prediction scores
    df_panss = pd.concat([df_panss, df_panss_v[[f'{visit}total', f'{visit}ptotal', f'{visit}ntotal', f'{visit}gtotal', f'{visit}ANDREASEN_ABS_CLASS', f'{visit}PANSS_ABSDIFF', f'{visit}PANSS_DIFF', f'{visit}PANSS_DIFF_CLASS',
                                                f'{visit}PANSS_POS_ABSDIFF', f'{visit}PANSS_POS_DIFF', f'{visit}PANSS_POS_DIFF_CLASS', f'{visit}PANSS_NEG_ABSDIFF', 
                                                f'{visit}PANSS_NEG_DIFF', f'{visit}PANSS_NEG_DIFF_CLASS', f'{visit}PANSS_NEG_REMISSION_ANDREASEN',
                                                f'{visit}PANSS_GEN_ABSDIFF', f'{visit}PANSS_GEN_DIFF', f'{visit}PANSS_GEN_DIFF_CLASS']]], axis=1)

#Create equivalent of PSD for negative symptoms higher than remission up to 6 months.
df_panss['V367PANSS_NEG_ANDREASEN_CLASS'] = df_panss.apply(lambda row: np.nan if (pd.isnull(row['V3PANSS_NEG_REMISSION_ANDREASEN']) and 
                                                                   pd.isnull(row['V6PANSS_NEG_REMISSION_ANDREASEN']) and 
                                                                   pd.isnull(row['V7PANSS_NEG_REMISSION_ANDREASEN'])) else 
                                            (0 if (row['V3PANSS_NEG_REMISSION_ANDREASEN'] == 0 or row['V6PANSS_NEG_REMISSION_ANDREASEN'] == 0 or row['V7PANSS_NEG_REMISSION_ANDREASEN'] == 0) else 1), axis=1)
    
#------------- Clinical global impression.--------------------------------
# OUTPUT dataframe: df_cgi
# - Replacement of 0's (not assessed) with NaN.
df_cgi = pd.read_excel(dir_input+'cgi_070727.xlsx', sheet_name = 'Data').filter(regex='^(crfnr)').replace(0, np.nan)    
# List of visit columns
visit_columns = ['V2', 'V3', 'V4', 'V5', 'V6', 'V7', 'V8', 'V9']

# Iterate through each visit
for visit in visit_columns:
    # Read the excel file for the specific visit and replace 0's with NaN
    df_cgi_v = pd.read_excel(dir_input+f'cgi_070727.xlsx', sheet_name='Data').filter(regex=f'^({visit}|crfnr)').replace(0, np.nan)

    #Define improvement (1) if CGI<4 and not improvement (0) if >= 4.
    df_cgi_v.loc[df_cgi_v[f'{visit}CGI2'] >= 4, f'{visit}CGI_CLASS'] = 0
    df_cgi_v.loc[df_cgi_v[f'{visit}CGI2'] < 4, f'{visit}CGI_CLASS'] = 1
    df_cgi_v.loc[df_cgi_v[f'{visit}CGI2'] == np.nan, f'{visit}CGI_CLASS'] = np.nan
    
    #Append to df_cgi
    df_cgi = pd.concat([df_cgi, df_cgi_v[[f'{visit}CGI2', f'{visit}CGI_CLASS']]], axis = 1)
    

#------------- Calgary Depression scale for Schizophrenia ----------------
# OUTPUT dataframe: df_cdss

visit_columns = ['V3', 'V6', 'V7', 'V9']

df_cdss = pd.read_excel(dir_input+f'cdss_070904.xlsx', sheet_name='Data').filter(regex=f'^(crfnr)').replace(9, np.nan)

# Iterate through each visit
for visit in visit_columns:
    # Read the excel file for the specific visit
    df_cdss_v = pd.read_excel(dir_input+f'cdss_070904.xlsx', sheet_name='Data').filter(regex=f'^(V1|{visit}|crfnr)').replace(9, np.nan)
    
    
    list_of_scores_v1 = ['V1CD01', 'V1CD02', 'V1CD03', 'V1CD04', 'V1CD05', 'V1CD06', 'V1CD07', 'V1CD08', 'V1CD09']

    #Add a total score variable.
    df_cdss_v['V1CDTOTAL'] = df_cdss_v[list_of_scores_v1].sum(axis = 1)

    #Assign nan when all values are nan. 
    df_cdss_v.loc[df_cdss_v[list_of_scores_v1].isna().all(axis = 1), 'V1CDTOTAL'] = np.nan

    #Define absolute presense (0) and absence of depression (1) based on CDSS criteria 
    #https://cumming.ucalgary.ca/research/calgary-depression-scale-schizophrenia/about-scale
    df_cdss_v.loc[df_cdss_v['V1CDTOTAL']>=7,'V1CD_ABS_CLASS'] = 0
    df_cdss_v.loc[df_cdss_v['V1CDTOTAL']<7,'V1CD_ABS_CLASS'] = 1
    df_cdss_v.loc[df_cdss_v['V1CDTOTAL'] == np.nan,'V1CD_ABS_CLASS'] = np.nan

    
    # List of CDSS score columns for the current visit
    list_of_scores = [f'{visit}CD01', f'{visit}CD02', f'{visit}CD03', f'{visit}CD04',
                      f'{visit}CD05', f'{visit}CD06', f'{visit}CD07', f'{visit}CD08',
                      f'{visit}CD09']
    
    # Add a total score variable
    df_cdss_v[f'{visit}CDTOTAL'] = df_cdss_v[list_of_scores].sum(axis=1)
    
    # Assign NaN when all values are NaN
    df_cdss_v.loc[df_cdss_v[list_of_scores].isna().all(axis=1), f'{visit}CDTOTAL'] = np.nan

    #Define absolute presence (0) and absence of depression (1) based on CDSS criteria
    #NOTE that label 0 is presence of depression because we are labelling 1 as the good outcome for all other labels. 
    #https://cumming.ucalgary.ca/research/calgary-depression-scale-schizophrenia/about-scale
    df_cdss_v.loc[df_cdss_v[f'{visit}CDTOTAL'] >= 7, f'{visit}CD_ABS_CLASS'] = 0
    df_cdss_v.loc[df_cdss_v[f'{visit}CDTOTAL'] < 7, f'{visit}CD_ABS_CLASS'] = 1
    df_cdss_v.loc[df_cdss_v[f'{visit}CDTOTAL'] == np.nan, f'{visit}CD_ABS_CLASS'] = np.nan
    
    #Define the absolute difference between visits. 
    df_cdss_v[f'{visit}CD_ABSDIFF'] = (df_cdss_v[f'{visit}CDTOTAL'] - df_cdss_v['V1CDTOTAL'])
    
    #Define the difference between visits. 
    #NOTE CDSS starts from 0.
    df_cdss_v[f'{visit}CD_DIFF'] = (df_cdss_v[f'{visit}CDTOTAL'] - df_cdss_v['V1CDTOTAL']) / (27 - 0)

    # Define good and bad treatment outcome as: ([CDSS total] decrease). 
    df_cdss_v.loc[df_cdss_v[f'{visit}CD_DIFF'] < 0, f'{visit}CD_DIFF_CLASS'] = 1
    df_cdss_v.loc[df_cdss_v[f'{visit}CD_DIFF'] >= 0, f'{visit}CD_DIFF_CLASS'] = 0
    df_cdss_v.loc[df_cdss_v[f'{visit}CD_DIFF'] == np.nan, f'{visit}CD_DIFF_CLASS'] = np.nan
    
    # Adding label for post-schizophrenic depression aprearance: ([CDSS total] increase > + 20%)
    #NOTE PSD is labelled as 0 to match the good outcome -> 1 and bad outcome -> 0 nomenclature. 
    df_cdss_v.loc[df_cdss_v[f'{visit}CD_DIFF'] <= 0.2, f'{visit}CD_PSD_CLASS'] = 1
    df_cdss_v.loc[df_cdss_v[f'{visit}CD_DIFF'] > 0.2, f'{visit}CD_PSD_CLASS'] = 0
    df_cdss_v.loc[df_cdss_v[f'{visit}CD_DIFF'] == np.nan, f'{visit}CD_PSD_CLASS'] = np.nan
    

    # Keep only relevant variables
    df_cdss = pd.concat([df_cdss, df_cdss_v[[f'{visit}CDTOTAL', f'{visit}CD_ABS_CLASS', f'{visit}CD_ABSDIFF', f'{visit}CD_DIFF', f'{visit}CD_DIFF_CLASS', f'{visit}CD_PSD_CLASS']]], axis=1)
    

## Addying the union post schizophrenic depression v1.
df_cdss['V3679CD_PSD_CLASS'] = df_cdss.apply(lambda row: np.nan if (pd.isnull(row['V3CD_ABS_CLASS']) and 
                                                                   pd.isnull(row['V6CD_ABS_CLASS']) and 
                                                                   pd.isnull(row['V7CD_ABS_CLASS']) and 
                                                                   pd.isnull(row['V9CD_ABS_CLASS'])) else 
                                            (0 if (row['V3CD_ABS_CLASS'] == 0 or row['V6CD_ABS_CLASS'] == 0 or row['V7CD_ABS_CLASS'] == 0 or row['V9CD_ABS_CLASS'] == 0) else 1), axis=1)

df_cdss['V367CD_PSD_CLASS'] = df_cdss.apply(lambda row: np.nan if (pd.isnull(row['V3CD_ABS_CLASS']) and 
                                                                   pd.isnull(row['V6CD_ABS_CLASS']) and 
                                                                   pd.isnull(row['V7CD_ABS_CLASS'])) else 
                                            (0 if (row['V3CD_ABS_CLASS'] == 0 or row['V6CD_ABS_CLASS'] == 0 or row['V7CD_ABS_CLASS'] == 0) else 1), axis=1)
```

In [32]:

```
################################################################################
#Storing Data and Merging
################################################################################
# Sergio Mena Ortega, 2024

#Writing individual datasets into excel.
## IMPORTANT: violators not removed, bear in mind they will have to be removed if using these.
df_gaf.to_excel(dir_output+'Individual Datasets/gaf_pred.xlsx', index = None)
df_cdss.to_excel(dir_output+'Individual Datasets/cdss_pred.xlsx', index = None)
df_cgi.to_excel(dir_output+'Individual Datasets/cgi_pred.xlsx', index = None)
df_panss.to_excel(dir_output+'Individual Datasets/panss_pred.xlsx', index = None)

## Merging treatment efficacy labels.

#Get violators IDs.
df_violators = pd.read_excel(dir_input+'protocol violators.xlsx', sheet_name = 'Data').filter(regex='^(VIOLATOR|crfnr)')
violators = df_violators.loc[df_violators['VIOLATOR'] == 1, 'crfnr']

#Get the original ids. 
df_id = pd.read_excel(dir_input+'v1_070727.xlsx', sheet_name = 'Data', usecols = ['crfnr'])

df_labels = df_id.copy()
list_of_dataframes = [df_gaf, df_cdss, df_cgi, df_panss] 
#Merge all labels into a dataframe.
for data in list_of_dataframes:
    df_labels = pd.merge(df_labels, data, on='crfnr', how='left')

#Remove protocol violator entries. 
df_labels = df_labels[~df_labels['crfnr'].isin(violators)]

#Save to excel.    
df_labels.to_excel(dir_output+'Merged Datasets/efficacy_labels.xlsx', index = False)
```
