# Supplementary material for: AI-based prediction of depression symptomatology in first-episode psychosis patients: insights from the EUFEST and RAISE-ETP clinical trials
Source: Psychol Med. 2025 Jul 30;55:e221. doi: 10.1017/S0033291725100950 (PMC12341035; doi:10.1017/S0033291725100950)
Supplement: Mena et al. supplementary material 2 — Mena et al. supplementary material [file S0033291725100950sup002.zip › supporting files/prediction_set_extraction_raiseetp.html]

prediction\_set\_extraction


In [27]:

```
import pandas as pd
import numpy as np

pd.set_option('display.max_rows', 5)
pd.set_option('display.max_columns', None)

#Folder name with original input data.
dir_input = 'Raw data/'

#Folder name for output data.
dir_output = 'Prediction set/'
```

In [28]:

```
################################################################################
#Data Extraction
################################################################################
# Sergio Mena Ortega, 2024


#------------- PANSS ---------------------------------------------
# OUTPUT dataframe: df_panss
df_panss = pd.read_excel(dir_input + 'panss01.xlsx', skiprows=[1]).filter(regex = "^(src_subject_id$|visit$)")
# Filter the visit, just baseline, and drop the visit column.  
df_panss = df_panss[df_panss['visit'] == 'B'].drop(columns = 'visit')

list_of_visits = ['B', 'M06', 'M12', 'M18', 'M24']

for visit in list_of_visits:
    # Filter the visit, just baseline, and drop the visit column.  
    df_panss_v = pd.read_excel(dir_input + 'panss01.xlsx', skiprows=[1]).filter(regex = "^(src_subject_id$|panss_total|pos_|neg_|gps_|visit$)")
    df_panss_v = df_panss_v[df_panss_v['visit'] == visit].drop(columns = 'visit')
    
    # Sum positive scores, negative scores, and general scores (gps) separately
    df_panss_v['panss_ptotal'] = df_panss_v.filter(like='pos_').sum(axis=1)
    df_panss_v['panss_ntotal'] = df_panss_v.filter(like='neg_').sum(axis=1)
    df_panss_v['panss_gtotal'] = df_panss_v.filter(like='gps_').sum(axis=1)
    
    df_panss = df_panss.merge(df_panss_v.rename(columns=lambda x: f'{visit}' + x if x != 'src_subject_id' else x), on='src_subject_id', how='left')

#Ge prediction variables for PANSS. 
for visit in list_of_visits[1:]:
    # Add a new column with 1's and 0's based on Andreasen criteria
    andresen_columns = [f'{visit}pos_p1', f'{visit}pos_p2', f'{visit}pos_p3', f'{visit}neg_n1', f'{visit}neg_n4', f'{visit}neg_n6', f'{visit}gps_g5', f'{visit}gps_g9']

    negative_columns = [f'{visit}neg_n1', f'{visit}neg_n2', f'{visit}neg_n3', f'{visit}neg_n4',
                        f'{visit}neg_n5', f'{visit}neg_n6', f'{visit}neg_n7']
    
    # Add column if meeting ANDREASEN criteria of remission
    df_panss[f'{visit}ANDREASEN_ABS_CLASS'] = (df_panss[andresen_columns] <= 3).all(axis=1).astype(int)
        
    # Set the value to NaN if either of Andreasen columns are nan
    df_panss.loc[df_panss[andresen_columns].isna().any(axis=1), f'{visit}ANDREASEN_ABS_CLASS'] = np.nan
    
    # Absolute change of total sum symptoms
    df_panss[f'{visit}PANSS_ABSDIFF'] = (df_panss[f'{visit}panss_total'] - df_panss['Bpanss_total'])
    
    # Percentage change of total sum symptoms
    #Note this is the Percentage of Maximum Possible Change, divided by the total possible change of the total PANSS score. 
    df_panss[f'{visit}PANSS_DIFF'] = (df_panss[f'{visit}panss_total'] - df_panss['Bpanss_total']) / (210 - 30)
    
    # Define good and bad treatment outcome as: ([PANSS total] decrease > + 20%)
    df_panss.loc[df_panss[f'{visit}PANSS_DIFF'] <= -0.2, f'{visit}PANSS_DIFF_CLASS20'] = 1
    df_panss.loc[df_panss[f'{visit}PANSS_DIFF'] > -0.2, f'{visit}PANSS_DIFF_CLASS20'] = 0
    df_panss.loc[df_panss[f'{visit}PANSS_DIFF'] == np.nan, f'{visit}PANSS_DIFF_CLASS20'] = np.nan

    # Define good and bad treatment outcome as: (increase or decrease)
    df_panss.loc[df_panss[f'{visit}PANSS_DIFF'] < 0, f'{visit}PANSS_DIFF_CLASS'] = 1
    df_panss.loc[df_panss[f'{visit}PANSS_DIFF'] >= 0, f'{visit}PANSS_DIFF_CLASS'] = 0
    df_panss.loc[df_panss[f'{visit}PANSS_DIFF'] == np.nan, f'{visit}PANSS_DIFF_CLASS'] = np.nan

    # Absolute change of total sum of positive symptoms
    df_panss[f'{visit}PANSS_POS_ABSDIFF'] = (df_panss[f'{visit}panss_ptotal'] - df_panss['Bpanss_ptotal'])
    
    # Percentage change of total sum of positive symptoms
    #Note this is the Percentage of Maximum Possible Change, divided by the total possible change of the total PANSS score. 
    df_panss[f'{visit}PANSS_POS_DIFF'] = (df_panss[f'{visit}panss_ptotal'] - df_panss['Bpanss_ptotal']) / (210 - 30)
    
    # Define good and bad treatment outcome as: ([PANSS total] decrease > + 20%)
    df_panss.loc[df_panss[f'{visit}PANSS_POS_DIFF'] <= -0.2, f'{visit}PANSS_POS_DIFF_CLASS20'] = 1
    df_panss.loc[df_panss[f'{visit}PANSS_POS_DIFF'] > -0.2, f'{visit}PANSS_POS_DIFF_CLASS20'] = 0
    df_panss.loc[df_panss[f'{visit}PANSS_POS_DIFF'] == np.nan, f'{visit}PANSS_POS_DIFF_CLASS20'] = np.nan

    # Define good and bad treatment outcome as: (increase or decrease)
    df_panss.loc[df_panss[f'{visit}PANSS_POS_DIFF'] < 0, f'{visit}PANSS_POS_DIFF_CLASS'] = 1
    df_panss.loc[df_panss[f'{visit}PANSS_POS_DIFF'] >= 0, f'{visit}PANSS_POS_DIFF_CLASS'] = 0
    df_panss.loc[df_panss[f'{visit}PANSS_POS_DIFF'] == np.nan, f'{visit}PANSS_POS_DIFF_CLASS'] = np.nan
    
    # Absolute change of total sum of negative symptoms
    df_panss[f'{visit}PANSS_NEG_ABSDIFF'] = (df_panss[f'{visit}panss_ntotal'] - df_panss['Bpanss_ntotal'])
    
    # Percentage change of total sum of negative symptoms
    #Note this is the Percentage of Maximum Possible Change, divided by the total possible change of the total PANSS score. 
    df_panss[f'{visit}PANSS_NEG_DIFF'] = (df_panss[f'{visit}panss_ntotal'] - df_panss['Bpanss_ntotal']) / (210 - 30)
    
    # Define good and bad treatment outcome as: ([PANSS total] decrease > + 20%)
    df_panss.loc[df_panss[f'{visit}PANSS_NEG_DIFF'] <= -0.2, f'{visit}PANSS_NEG_DIFF_CLASS20'] = 1
    df_panss.loc[df_panss[f'{visit}PANSS_NEG_DIFF'] > -0.2, f'{visit}PANSS_NEG_DIFF_CLASS20'] = 0
    df_panss.loc[df_panss[f'{visit}PANSS_NEG_DIFF'] == np.nan, f'{visit}PANSS_NEG_DIFF_CLASS20'] = np.nan

        # Define good and bad treatment outcome as: (increase or decrease)
    df_panss.loc[df_panss[f'{visit}PANSS_NEG_DIFF'] < 0, f'{visit}PANSS_NEG_DIFF_CLASS'] = 1
    df_panss.loc[df_panss[f'{visit}PANSS_NEG_DIFF'] >= 0, f'{visit}PANSS_NEG_DIFF_CLASS'] = 0
    df_panss.loc[df_panss[f'{visit}PANSS_NEG_DIFF'] == np.nan, f'{visit}PANSS_NEG_DIFF_CLASS'] = np.nan

    # Create a new column that checks if all negative symptoms are ≤ 3
    df_panss[f'{visit}PANSS_NEG_REMISSION_ANDREASEN'] = (df_panss[negative_columns] <= 3).all(axis=1).astype(int)

    # Set the value to NaN if any of the negative columns are NaN
    df_panss.loc[df_panss[negative_columns].isna().any(axis=1), f'{visit}PANSS_NEG_REMISSION_ANDREASEN'] = np.nan
    
    # Absolute change of total sum of general symptoms
    df_panss[f'{visit}PANSS_GEN_ABSDIFF'] = (df_panss[f'{visit}panss_gtotal'] - df_panss['Bpanss_gtotal'])
    
    # Percentage change of total sum of negative symptoms
    #Note this is the Percentage of Maximum Possible Change, divided by the total possible change of the total PANSS score. 
    df_panss[f'{visit}PANSS_GEN_DIFF'] = (df_panss[f'{visit}panss_gtotal'] - df_panss['Bpanss_gtotal']) / (210 - 30)
    
    # Define good and bad treatment outcome as: ([PANSS total] decrease > + 20%)
    df_panss.loc[df_panss[f'{visit}PANSS_GEN_DIFF'] <= -0.2, f'{visit}PANSS_GEN_DIFF_CLASS20'] = 1
    df_panss.loc[df_panss[f'{visit}PANSS_GEN_DIFF'] > -0.2, f'{visit}PANSS_GEN_DIFF_CLASS20'] = 0
    df_panss.loc[df_panss[f'{visit}PANSS_GEN_DIFF'] == np.nan, f'{visit}PANSS_GEN_DIFF_CLASS20'] = np.nan

    # Define good and bad treatment outcome as: (increase or decrease)
    df_panss.loc[df_panss[f'{visit}PANSS_GEN_DIFF'] < 0, f'{visit}PANSS_GEN_DIFF_CLASS'] = 1
    df_panss.loc[df_panss[f'{visit}PANSS_GEN_DIFF'] >= 0, f'{visit}PANSS_GEN_DIFF_CLASS'] = 0
    df_panss.loc[df_panss[f'{visit}PANSS_GEN_DIFF'] == np.nan, f'{visit}PANSS_GEN_DIFF_CLASS'] = np.nan

df_panss['MJust6PANSS_NEG_ANDREASEN_CLASS'] = df_panss.apply(lambda row: np.nan if (pd.isnull(row['M06PANSS_NEG_REMISSION_ANDREASEN'])) else 
                                            (0 if (row['M06PANSS_NEG_REMISSION_ANDREASEN'] == 0) else 1), axis=1)

## Filter out unwanted columns.
df_panss = df_panss.filter(regex = "DIFF|CLASS|total|src_subject_id")

#Remove baseline variables.
df_panss.drop(columns = ['Bpanss_total','Bpanss_ptotal', 'Bpanss_ntotal', 'Bpanss_gtotal'], inplace = True)

#-----------------------------------------------------------------


#------------- Calgary Depression scale for Schizophrenia ----------------
# OUTPUT dataframe: df_cdss
df_cdss = pd.read_excel(dir_input + 'clgry01.xlsx', skiprows=[1]).filter(regex = "^(src_subject_id$|visit$)")

# Filter the visit, just baseline, and drop the visit column.  
df_cdss = df_cdss[df_cdss['visit'] == 'B'].drop(columns = 'visit')


list_of_visits = ['B', 'M06', 'M12', 'M18', 'M24']

for visit in list_of_visits:
    df_cdss_v = pd.read_excel(dir_input + 'clgry01.xlsx', skiprows=[1]).filter(regex = "^(src_subject_id$|calg|visit$)")
    df_cdss_v = df_cdss_v[df_cdss_v['visit'] == visit].drop(columns = 'visit')
    ##Remove irrelevant cols. 
    df_cdss_v.drop(columns = ['calg_s1', 'calg10', 'calg_s2'], inplace = True)


    ##IMPORTANT: calgary is from 0 to 3, here it seems to start from 1, so we remove 1 to each col.
    calg_columns = ['calg1', 'calg2', 'calg3', 'calg4', 'calg5', 'calg6', 'calg7', 'calg8', 'calg9']
    df_cdss_v[calg_columns] = df_cdss_v[calg_columns].sub(1)
    
    # Recalculate the total in the calg_ts column
    df_cdss_v['calg_ts'] = df_cdss_v[calg_columns].sum(axis=1)
    
    #Add to main df. 
    df_cdss = df_cdss.merge(df_cdss_v.rename(columns=lambda x: f'{visit}' + x if x != 'src_subject_id' else x), on='src_subject_id', how='left')

#Ge prediction variables for PANSS. 
for visit in list_of_visits[1:]:
    #Define absolute presence (0) and absence of depression (1) based on CDSS criteria
    #NOTE that label 0 is presence of depression because we are labelling 1 as the good outcome for all other labels. 
    #https://cumming.ucalgary.ca/research/calgary-depression-scale-schizophrenia/about-scale
    df_cdss.loc[df_cdss[f'{visit}calg_ts'] >= 7, f'{visit}CD_ABS_CLASS'] = 0
    df_cdss.loc[df_cdss[f'{visit}calg_ts'] < 7, f'{visit}CD_ABS_CLASS'] = 1
    df_cdss.loc[df_cdss[f'{visit}calg_ts'] == np.nan, f'{visit}CD_ABS_CLASS'] = np.nan
    
    #Define the absolute difference between visits. 
    df_cdss[f'{visit}CD_ABSDIFF'] = (df_cdss[f'{visit}calg_ts'] - df_cdss['Bcalg_ts'])
    
    #Define the difference between visits. 
    #NOTE CDSS starts from 0.
    df_cdss[f'{visit}CD_DIFF'] = (df_cdss[f'{visit}calg_ts'] - df_cdss['Bcalg_ts']) / (27 - 0)
    
    # Define good and bad treatment outcome as: ([CDSS total] decrease). 
    df_cdss.loc[df_cdss[f'{visit}CD_DIFF'] < 0, f'{visit}CD_DIFF_CLASS'] = 1
    df_cdss.loc[df_cdss[f'{visit}CD_DIFF'] >= 0, f'{visit}CD_DIFF_CLASS'] = 0
    df_cdss.loc[df_cdss[f'{visit}CD_DIFF'] == np.nan, f'{visit}CD_DIFF_CLASS'] = np.nan
    
    # Adding label for post-schizophrenic depression aprearance: ([CDSS total] increase > + 20%)
    #NOTE PSD is labelled as 0 to match the good outcome -> 1 and bad outcome -> 0 nomenclature. 
    df_cdss.loc[df_cdss[f'{visit}CD_DIFF'] <= 0.2, f'{visit}CD_PSD_CLASS'] = 1
    df_cdss.loc[df_cdss[f'{visit}CD_DIFF'] > 0.2, f'{visit}CD_PSD_CLASS'] = 0
    df_cdss.loc[df_cdss[f'{visit}CD_DIFF'] == np.nan, f'{visit}CD_PSD_CLASS'] = np.nan

## Addying the union post schizophrenic depression.
df_cdss['MAllCD_PSD_CLASS'] = df_cdss.apply(lambda row: np.nan if (pd.isnull(row['M06CD_ABS_CLASS']) and 
                                                                   pd.isnull(row['M12CD_ABS_CLASS']) and pd.isnull(row['M18CD_ABS_CLASS']) and pd.isnull(row['M24CD_ABS_CLASS'])) else 
                                            (0 if (row['M06CD_ABS_CLASS'] == 0 or row['M12CD_ABS_CLASS'] == 0 or row['M18CD_ABS_CLASS'] == 0 or row['M24CD_ABS_CLASS'] == 0) else 1), axis=1)

df_cdss['MJust6CD_PSD_CLASS'] = df_cdss.apply(lambda row: np.nan if (pd.isnull(row['M06CD_ABS_CLASS'])) else 
                                            (0 if (row['M06CD_ABS_CLASS'] == 0) else 1), axis=1)
## Filter out unwanted columns.
df_cdss = df_cdss.filter(regex = "DIFF|CLASS|calg_ts|src_subject_id")
df_cdss.drop(columns = ['Bcalg_ts'], inplace = True)

#-----------------------------------------------------------------
```

In [29]:

```
################################################################################
#Storing Data and Merging
################################################################################
# Sergio Mena Ortega, 2024

#Writing individual datasets into excel.
df_cdss.to_excel(dir_output+'Individual Datasets/cdss_pred.xlsx', index = None)
df_panss.to_excel(dir_output+'Individual Datasets/panss_pred.xlsx', index = None)

## Merging treatment efficacy labels.

#Get the original ids. 
df_id =  pd.read_excel(dir_input + 'ptchart01.xlsx', skiprows=[1], nrows=404).filter(regex="^(src_subject_id$|visit$)")

# Filter the visit, just baseline, and drop the visit column.  
df_id = df_id[df_id['visit'] == 'B'].drop(columns = 'visit')


df_labels = df_id.copy()
list_of_dataframes = [df_cdss, df_panss] 
#Merge all labels into a dataframe.
for data in list_of_dataframes:
    df_labels = pd.merge(df_labels, data, on='src_subject_id', how='left')

#Save to excel.    
df_labels.to_excel(dir_output+'Merged Datasets/efficacy_labels.xlsx', index = False)
```
